# Supplementary material for: Efficacy of Management and Monitoring Methods to Prevent Post-Harvest Losses Caused by Rodents
Source: Animals (Basel). 2020 Sep 9;10(9):1612. doi: 10.3390/ani10091612 (PMC7552224; doi:10.3390/ani10091612)
Supplement: Supplementary file 1 [file animals-10-01612-s001.zip › animals-916308-supplementary.pdf]

# Mixed model analysis of rodent management in Bangladesh

*Gerrit Gort and Inge Krijger*

*19-05-2020*

## Analyze data on rice loss per day

### Overview plot of data on rice loss per day

```
setwd("C:/MyData/OneDrive - WageningenUR/Data/Inge Krijger/final/Rice")

R <- read.csv("Overview Total_GrainLossAssessment_Villages-2016+2017.csv")

R <- R[R$loss > 0,]  # remove first observations, which contain starting weight

R$hh <- factor(R$hh)
R$year <- factor(R$year)
R$intervalc <- factor(R$interval2)

sd.iv <- sd(R$intervaldays)
R$iv1 <- (R$intervaldays)/sd(R$intervaldays)
R$iv2 <- R$iv1*R$iv1
R$iv3 <- R$iv1*R$iv1*R$iv1

R$treatment <- relevel(R$treatment, ref="control")

R$treatment2 <- R$treatment
R$treatment2[R$interval2<0] <- "control"

R$v.hh <- factor(paste(R$village, R$hh, sep="."))
R$v.hh.y <- factor(paste(R$village, R$hh, substr(as.character(R$year),4,4), sep="."))

R$YS <- factor(paste(R$year, R$season, sep="."), levels=c("2016.wet","2016.dry","2017.wet","2017.dry"))
# Note: order of levels of R$YS is now: "2016.wet" "2016.dry" "2017.wet" "2017.dry"

R$y2 <- log10(R$loss_pd+1)

man.col <- c("#F8766D", "#7CAE00", "#C77CFF", "#00BFC4")

# plot with logarithmic axis and different theme
ggplot(data=R, aes(x=intervaldays, y=y2, colour=treatment2, group=v.hh)) +
  theme_bw()+ theme(panel.grid.minor = element_blank()) +
  geom_line(size=0.3, alpha=0.35) + facet_wrap(~YS) +
  scale_x_continuous(breaks=c(-28,-14,0,14,28,42,56,70), labels=c("-28","-14","0","14","28","42","56","70")) +
  labs(x = "days since start treatment", y = "rice loss per day (g)") +
  scale_y_continuous(breaks=c(0.0414,0.3010,0.7782,1.0414,1.7076,2.0043), labels=c("0.1","1","5","10","50","10
  guides(colour = guide_legend(override.aes = list(alpha = 1), title="treatment")) +
  scale_color_manual(values=man.col)
```

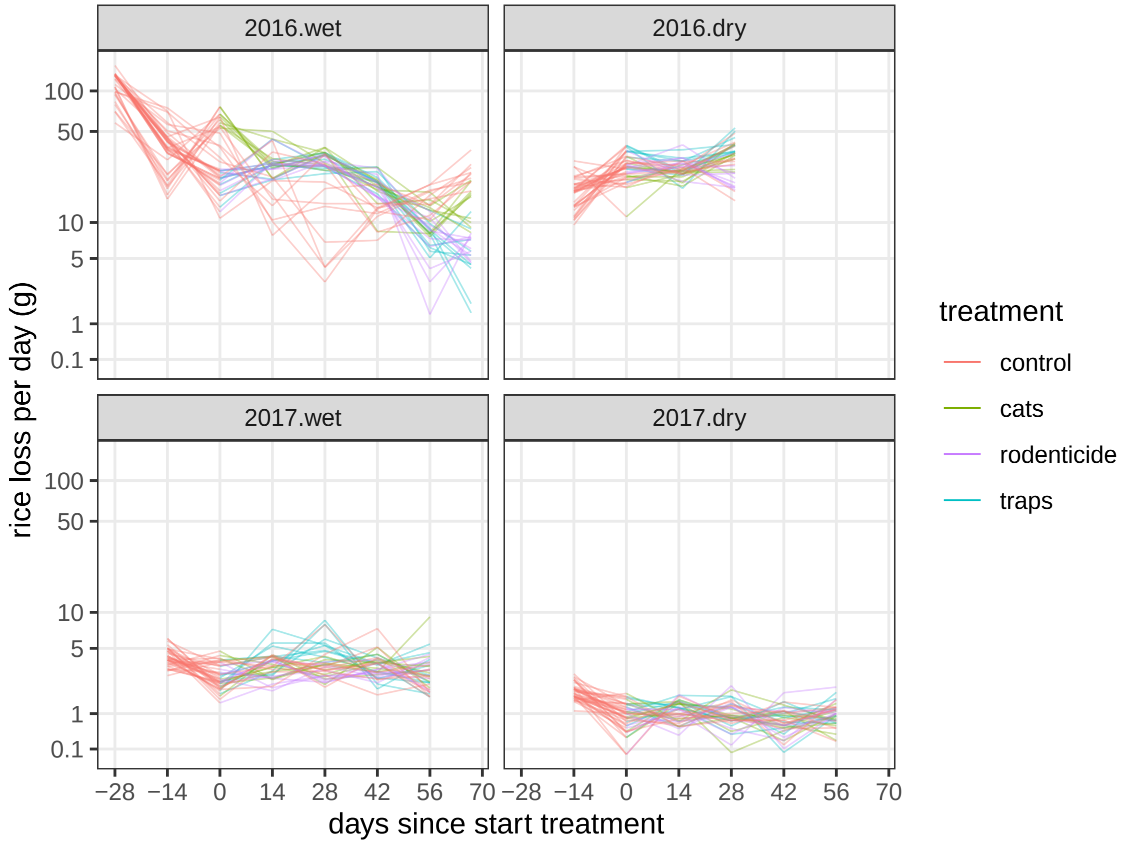


## Data on rice loss per day: mixed model analysis

1) We fit a mixed model for log-transformed daily rice loss. The fixed part of the model contains a common quadratic time trend up until timepoint 0, and thereafter treatment specific quadratic time trends, all year-season specific. The random part allows for random household specific quadratic time trends, random village by year effects and random village by year by time effects.
2) To check whether higher order trends are needed we fit a cubic version of the model mentioned above, and compare model fits using an F-test.
3) We compare the year-season specific time trends, first by comparing all four year-season combinations simultaneously, and next pairwise, using F-tests.
4) Within each year-season combination we compare the time trends among the four treatments, using F-tests.

```
Rlmero <- lmer(y2 ~ -1 + year:season + year:season:treatment2:iv1 + year:season:treatment2:iv2
                + (1 | village:year) + (1 | village:year:intervalc)
                + (1 + iv1 + iv2 | year:v.hh),
                data=R, REML=TRUE,
              control = lmerControl(optimizer="bobyqa", optCtrl = list(maxfun = 100000)))
```

```
## boundary (singular) fit: see ?isSingular
```

```
Rlmero.iv3 <- lmer(y2 ~
                  year:season + year:season:treatment2:iv1 + year:season:treatment2:iv2
                + year:season:treatment2:iv3
                + (1 | village:year) + (1 | village:year:intervalc)
                + (1 + iv1 + iv2 + iv3 | year:v.hh),
                data=R, REML=TRUE,
                control = lmerControl(optimizer="bobyqa", optCtrl = list(maxfun = 100000)))
```

```
## fixed-effect model matrix is rank deficient so dropping 4 columns / coefficients
## boundary (singular) fit: see ?isSingular
```

```
## fixed-effect model matrix is rank deficient so dropping 4 columns / coefficients
```

```r
KRmodcomp(Rlmero, Rlmero.iv3)
```

```
## F-test with Kenward-Roger approximation; time: 5.99 sec
## large : y2 ~ year:season + year:season:treatment2:iv1 + year:season:treatment2:iv2 +
##     year:season:treatment2:iv3 + (1 | village:year) + (1 | village:year:intervalc) +
##     (1 + iv1 + iv2 + iv3 | year:v.hh)
## small : y2 ~ -1 + year:season + year:season:treatment2:iv1 + year:season:treatment2:iv2 +
##     (1 | village:year) + (1 | village:year:intervalc) + (1 +
##     iv1 + iv2 | year:v.hh)
##          stat     ndf     ddf F.scaling p.value
## Ftest  0.9219 13.0000 39.9043   0.99977  0.5398
```

```r
# Model with cubic terms (both in fixed and random part) not better

# We continue with the quadratic model
summary(Rlmero)
```

```
## Linear mixed model fit by REML. t-tests use Satterthwaite's method [
## lmerModLmerTest]
## Formula:
## y2 ~ -1 + year:season + year:season:treatment2:iv1 + year:season:treatment2:iv2 +
##     (1 | village:year) + (1 | village:year:intervalc) + (1 +
##     iv1 + iv2 | year:v.hh)
##    Data: R
## Control: lmerControl(optimizer = "bobyqa", optCtrl = list(maxfun = 1e+05))
##
## REML criterion at convergence: -1298.6
##
## Scaled residuals:
##     Min      1Q  Median      3Q     Max
## -4.8979 -0.4858  0.0384  0.5486  4.2695
##
## Random effects:
##  Groups                 Name        Variance  Std.Dev. Corr
##  year:v.hh              (Intercept) 3.216e-05 0.005671
##                         iv1         5.869e-05 0.007661  1.00
##                         iv2         8.094e-05 0.008996 -1.00 -1.00
##  village:year:intervalc (Intercept) 8.208e-03 0.090597
##  village:year           (Intercept) 0.000e+00 0.000000
##  Residual                           1.073e-02 0.103569
## Number of obs: 959, groups:
## year:v.hh, 160; village:year:intervalc, 96; village:year, 16
##
## Fixed effects:
##                                          Estimate Std. Error
## year2016:seasondry                       1.426050   0.043673
## year2017:seasondry                       0.328492   0.034120
## year2016:seasonwet                       1.419402   0.031705
## year2017:seasonwet                       0.634841   0.034119
## year2016:seasondry:treatment2control:iv1      0.228316   0.072810
## year2017:seasondry:treatment2control:iv1     -0.169079   0.067196
## year2016:seasonwet:treatment2control:iv1     -0.408402   0.032026
## year2017:seasonwet:treatment2control:iv1     -0.069649   0.067196
## year2016:seasondry:treatment2cats:iv1       -0.255652   0.378778
## year2017:seasondry:treatment2cats:iv1       -0.074843   0.147261
## year2016:seasonwet:treatment2cats:iv1        0.090313   0.114920
## year2017:seasonwet:treatment2cats:iv1        0.001757   0.147152
## year2016:seasondry:treatment2rodenticide:iv1  0.218319   0.378778
## year2017:seasondry:treatment2rodenticide:iv1 -0.128578   0.147152
## year2016:seasonwet:treatment2rodenticide:iv1  0.178463   0.114920
```

```
## year2017:seasonwet:treatment2rodenticide:iv1 -0.074230   0.147152
## year2016:seasondry:treatment2traps:iv1       -0.119591   0.378778
## year2017:seasondry:treatment2traps:iv1       -0.035730   0.147152
## year2016:seasonwet:treatment2traps:iv1        0.256458   0.114920
## year2017:seasonwet:treatment2traps:iv1        0.235485   0.147152
## year2016:seasondry:treatment2control:iv2     -0.164960   0.108252
## year2017:seasondry:treatment2control:iv2      0.075274   0.043125
## year2016:seasonwet:treatment2control:iv2      0.151234   0.020166
## year2017:seasonwet:treatment2control:iv2      0.022734   0.043125
## year2016:seasondry:treatment2cats:iv2         0.315788   0.360840
## year2017:seasondry:treatment2cats:iv2         0.014975   0.076717
## year2016:seasonwet:treatment2cats:iv2        -0.086919   0.050475
## year2017:seasonwet:treatment2cats:iv2         0.002787   0.076692
## year2016:seasondry:treatment2rodenticide:iv2 -0.244060   0.360840
## year2017:seasondry:treatment2rodenticide:iv2  0.057190   0.076692
## year2016:seasonwet:treatment2rodenticide:iv2 -0.168369   0.050475
## year2017:seasonwet:treatment2rodenticide:iv2  0.030569   0.076692
## year2016:seasondry:treatment2traps:iv2        0.264215   0.360840
## year2017:seasondry:treatment2traps:iv2        0.007499   0.076692
## year2016:seasonwet:treatment2traps:iv2       -0.205721   0.050475
## year2017:seasonwet:treatment2traps:iv2       -0.125087   0.076692
##                                                     df t value Pr(>|t|)
## year2016:seasondry                           59.992744  32.653  < 2e-16
## year2017:seasondry                           60.077483   9.627 8.70e-14
## year2016:seasonwet                           60.104472  44.769  < 2e-16
## year2017:seasonwet                           60.072514  18.606  < 2e-16
## year2016:seasondry:treatment2control:iv1     59.986304   3.136 0.002654
## year2017:seasondry:treatment2control:iv1     60.017724  -2.516 0.014554
## year2016:seasonwet:treatment2control:iv1     60.104544 -12.752  < 2e-16
## year2017:seasonwet:treatment2control:iv1     60.017083  -1.037 0.304122
## year2016:seasondry:treatment2cats:iv1        59.974456  -0.675 0.502308
## year2017:seasondry:treatment2cats:iv1        60.269791  -0.508 0.613143
## year2016:seasonwet:treatment2cats:iv1        60.150088   0.786 0.435025
## year2017:seasonwet:treatment2cats:iv1        60.091916   0.012 0.990511
## year2016:seasondry:treatment2rodenticide:iv1 59.974456   0.576 0.566517
## year2017:seasondry:treatment2rodenticide:iv1 60.092624  -0.874 0.385719
## year2016:seasonwet:treatment2rodenticide:iv1 60.150088   1.553 0.125684
## year2017:seasonwet:treatment2rodenticide:iv1 60.091916  -0.504 0.615794
## year2016:seasondry:treatment2traps:iv1       59.974456  -0.316 0.753306
## year2017:seasondry:treatment2traps:iv1       60.092624  -0.243 0.808982
## year2016:seasonwet:treatment2traps:iv1       60.150088   2.232 0.029377
## year2017:seasonwet:treatment2traps:iv1       60.091916   1.600 0.114779
## year2016:seasondry:treatment2control:iv2     59.957956  -1.524 0.132806
## year2017:seasondry:treatment2control:iv2     60.252001   1.745 0.085999
## year2016:seasonwet:treatment2control:iv2     60.778815   7.499 3.27e-10
## year2017:seasonwet:treatment2control:iv2     60.250519   0.527 0.600014
## year2016:seasondry:treatment2cats:iv2        59.962813   0.875 0.384987
## year2017:seasondry:treatment2cats:iv2        60.298080   0.195 0.845898
## year2016:seasonwet:treatment2cats:iv2        60.456781  -1.722 0.090177
## year2017:seasonwet:treatment2cats:iv2        60.219250   0.036 0.971134
## year2016:seasondry:treatment2rodenticide:iv2 59.962813  -0.676 0.501409
## year2017:seasondry:treatment2rodenticide:iv2 60.219562   0.746 0.458746
## year2016:seasonwet:treatment2rodenticide:iv2 60.456781  -3.336 0.001458
## year2017:seasonwet:treatment2rodenticide:iv2 60.219250   0.399 0.691603
## year2016:seasondry:treatment2traps:iv2       59.962813   0.732 0.466887
## year2017:seasondry:treatment2traps:iv2       60.219562   0.098 0.922433
## year2016:seasonwet:treatment2traps:iv2       60.456781  -4.076 0.000136
## year2017:seasonwet:treatment2traps:iv2       60.219250  -1.631 0.108103

##
```

```
## Correlation matrix not shown by default, as p = 36 > 12.
## Use print(x, correlation=TRUE)  or
##     vcov(x)        if you need it

## convergence code: 0
## boundary (singular) fit: see ?isSingular
```

```
# Residual analysis using DHARMa
simulationOutput <- simulateResiduals(fittedModel = Rlmero, n=5000, plot=TRUE)
```

```
## Model family was recognized or set as continuous, but duplicate values were detected in the response. Consi
```

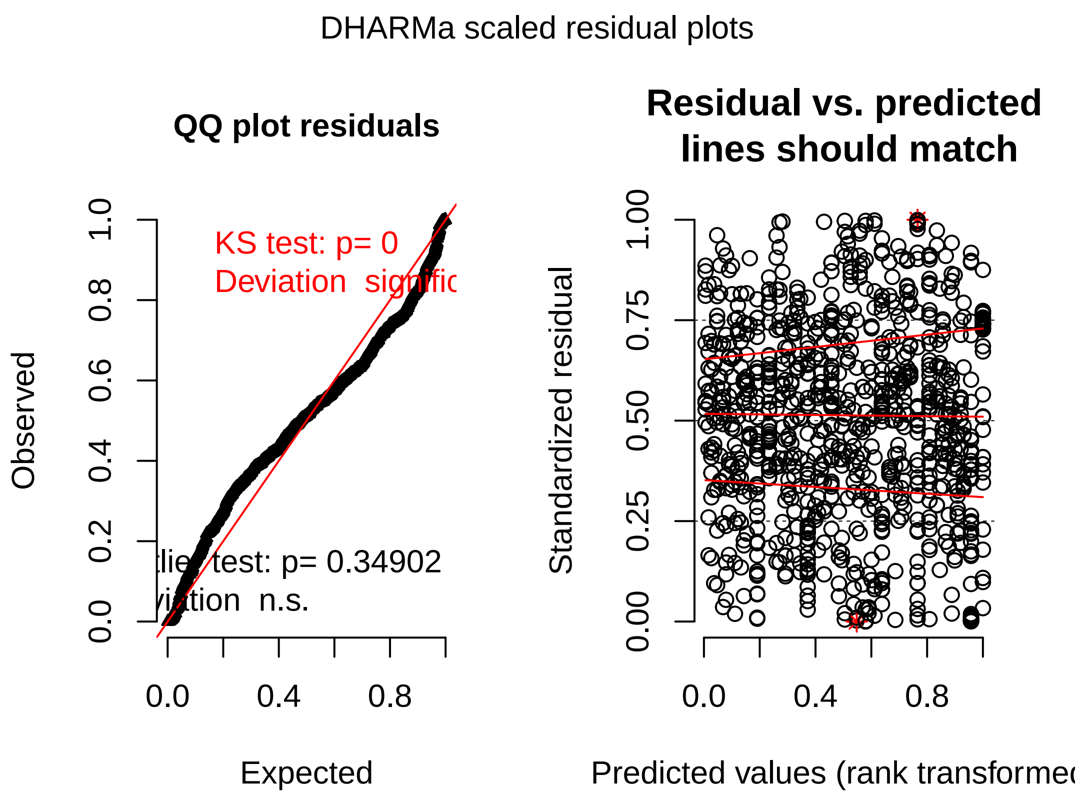


```
# We find slight deviations in the residual patterns.
# The small P-value for the KS test is not too disturbing, given the size of the dataset (n=959)


# Compare treatment time trends between year-season combinations
Rlmero.YS <- lmer(y2 ~ treatment2:iv1 + treatment2:iv2
                  + (1 | village:year) + (1 | village:year:intervalc)
                  + (1 + iv1 + iv2 | year:v.hh),
                  data=R, REML=TRUE,
                  control = lmerControl(optimizer="bobyqa", optCtrl = list(maxfun = 100000)))
```

```
## boundary (singular) fit: see ?isSingular
```

```
KRmodcomp(Rlmero, Rlmero.YS)
```

```
## F-test with Kenward-Roger approximation; time: 2.43 sec
## large : y2 ~ -1 + year:season + year:season:treatment2:iv1 + year:season:treatment2:iv2 +
##     (1 | village:year) + (1 | village:year:intervalc) + (1 +
##     iv1 + iv2 | year:v.hh)
## small : y2 ~ treatment2:iv1 + treatment2:iv2 + (1 | village:year) + (1 |
##     village:year:intervalc) + (1 + iv1 + iv2 | year:v.hh)
##          stat     ndf     ddf F.scaling   p.value
## Ftest 83.9402 27.0000  9.6754   0.98332 1.938e-08
```

```r
# Treatment time trends are different among year-season combinations

# Order of R$YS is: "2016.wet" "2016.dry" "2017.wet" "2017.dry"

# 2016-wet versus 2016-dry:
R$YS12 <- R$YS; levels(R$YS12) <- c(1,1,3,4)
Rlmero.12 <- lmer(y2 ~ -1 + YS12 + YS12:treatment2:iv1 + YS12:treatment2:iv2
                + (1 | village:year) + (1 | village:year:intervalc)
                + (1 + iv1 + iv2 | year:v.hh),
                data=R, REML=TRUE,
                control = lmerControl(optimizer="bobyqa", optCtrl = list(maxfun = 100000)))
```

```
## boundary (singular) fit: see ?isSingular
```

```r
KRmodcomp(Rlmero, Rlmero.12)
```

```
## F-test with Kenward-Roger approximation; time: 2.39 sec
## large : y2 ~ -1 + year:season + year:season:treatment2:iv1 + year:season:treatment2:iv2 +
##     (1 | village:year) + (1 | village:year:intervalc) + (1 +
##     iv1 + iv2 | year:v.hh)
## small : y2 ~ -1 + YS12 + YS12:treatment2:iv1 + YS12:treatment2:iv2 +
##     (1 | village:year) + (1 | village:year:intervalc) + (1 +
##     iv1 + iv2 | year:v.hh)
##          stat     ndf     ddf F.scaling   p.value
## Ftest  8.6497  9.0000 28.7807   0.95069 3.801e-06
```

```r
# Time trends in 2016-wet and 2016-dry are significantly different

# 2016-wet versus 2017-wet:
R$YS13 <- R$YS; levels(R$YS13) <- c(1,2,1,4)
Rlmero.13 <- lmer(y2 ~ -1 + YS13 + YS13:treatment2:iv1 + YS13:treatment2:iv2
                  + (1 | village:year) + (1 | village:year:intervalc)
                  + (1 + iv1 + iv2 | year:v.hh),
                  data=R, REML=TRUE,
                  control = lmerControl(optimizer="bobyqa", optCtrl = list(maxfun = 100000)))
```

```
## boundary (singular) fit: see ?isSingular
```

```r
KRmodcomp(Rlmero, Rlmero.13)
```

```
## F-test with Kenward-Roger approximation; time: 2.36 sec
## large : y2 ~ -1 + year:season + year:season:treatment2:iv1 + year:season:treatment2:iv2 +
##     (1 | village:year) + (1 | village:year:intervalc) + (1 +
##     iv1 + iv2 | year:v.hh)
## small : y2 ~ -1 + YS13 + YS13:treatment2:iv1 + YS13:treatment2:iv2 +
##     (1 | village:year) + (1 | village:year:intervalc) + (1 +
##     iv1 + iv2 | year:v.hh)
##         stat    ndf    ddf F.scaling   p.value
## Ftest 79.151  9.000 10.408   0.89429 2.356e-08
```

```r
# Time trends in 2016-wet and 2017-wet are significantly different

# 2016-wet versus 2017-dry:
R$YS14 <- R$YS; levels(R$YS14) <- c(1,2,3,1)
Rlmero.14 <- lmer(y2 ~ -1 + YS14 + YS14:treatment2:iv1 + YS14:treatment2:iv2
                  + (1 | village:year) + (1 | village:year:intervalc)
                  + (1 + iv1 + iv2 | year:v.hh),
                  data=R, REML=TRUE,
                  control = lmerControl(optimizer="bobyqa", optCtrl = list(maxfun = 100000)))
```

```
## boundary (singular) fit: see ?isSingular
```

```r
KRmodcomp(Rlmero, Rlmero.14)
```

```
## F-test with Kenward-Roger approximation; time: 2.20 sec
## large : y2 ~ -1 + year:season + year:season:treatment2:iv1 + year:season:treatment2:iv2 +
##     (1 | village:year) + (1 | village:year:intervalc) + (1 +
##     iv1 + iv2 | year:v.hh)
## small : y2 ~ -1 + YS14 + YS14:treatment2:iv1 + YS14:treatment2:iv2 +
##     (1 | village:year) + (1 | village:year:intervalc) + (1 +
##     iv1 + iv2 | year:v.hh)
##          stat     ndf     ddf F.scaling   p.value
## Ftest 156.364   9.000  10.418   0.89432 7.106e-10
```

```r
# Time trends in 2016-wet and 2017-dry are significantly different

# 2016-dry versus 2017-wet:
R$YS23 <- R$YS; levels(R$YS23) <- c(1,2,2,4)
Rlmero.23 <- lmer(y2 ~ -1 + YS23 + YS23:treatment2:iv1 + YS23:treatment2:iv2
                  + (1 | village:year) + (1 | village:year:intervalc)
                  + (1 + iv1 + iv2 | year:v.hh),
                  data=R, REML=TRUE,
                  control = lmerControl(optimizer="bobyqa", optCtrl = list(maxfun = 100000)))
```

```
## boundary (singular) fit: see ?isSingular
```

```r
KRmodcomp(Rlmero, Rlmero.23)
```

```
## F-test with Kenward-Roger approximation; time: 2.18 sec
## large : y2 ~ -1 + year:season + year:season:treatment2:iv1 + year:season:treatment2:iv2 +
##     (1 | village:year) + (1 | village:year:intervalc) + (1 +
##     iv1 + iv2 | year:v.hh)
## small : y2 ~ -1 + YS23 + YS23:treatment2:iv1 + YS23:treatment2:iv2 +
##     (1 | village:year) + (1 | village:year:intervalc) + (1 +
##     iv1 + iv2 | year:v.hh)
##         stat    ndf    ddf F.scaling   p.value
## Ftest 56.876  9.000 30.174    0.9542 < 2.2e-16
```

```r
# Time trends in 2016-dry and 2017-wet are significantly different

# 2016-dry versus 2017-dry:
R$YS24 <- R$YS; levels(R$YS24) <- c(1,2,3,2)
Rlmero.24 <- lmer(y2 ~ -1 + YS24 + YS24:treatment2:iv1 + YS24:treatment2:iv2
                  + (1 | village:year) + (1 | village:year:intervalc)
                  + (1 + iv1 + iv2 | year:v.hh),
                  data=R, REML=TRUE,
                  control = lmerControl(optimizer="bobyqa", optCtrl = list(maxfun = 100000)))
```

```
## boundary (singular) fit: see ?isSingular
```

```r
KRmodcomp(Rlmero, Rlmero.24)
```

```
## F-test with Kenward-Roger approximation; time: 2.11 sec
## large : y2 ~ -1 + year:season + year:season:treatment2:iv1 + year:season:treatment2:iv2 +
##     (1 | village:year) + (1 | village:year:intervalc) + (1 +
##     iv1 + iv2 | year:v.hh)
## small : y2 ~ -1 + YS24 + YS24:treatment2:iv1 + YS24:treatment2:iv2 +
##     (1 | village:year) + (1 | village:year:intervalc) + (1 +
##     iv1 + iv2 | year:v.hh)
##          stat     ndf     ddf F.scaling   p.value
## Ftest 115.004   9.000  30.182   0.95421 < 2.2e-16
```

```r
# Time trends in 2016-dry and 2017-dry are significantly different

# 2017-wet versus 2017-dry:
```

```r
R$YS34 <- R$YS; levels(R$YS34) <- c(1,2,3,3)
Rlmero.34 <- lmer(y2 ~ -1 + YS34 + YS34:treatment2:iv1 + YS34:treatment2:iv2
                  + (1 | village:year) + (1 | village:year:intervalc)
                  + (1 + iv1 + iv2 | year:v.hh),
                  data=R, REML=TRUE,
                  control = lmerControl(optimizer="bobyqa", optCtrl = list(maxfun = 100000)))
```

```
## boundary (singular) fit: see ?isSingular
```

```r
KRmodcomp(Rlmero, Rlmero.34)
```

```
## F-test with Kenward-Roger approximation; time: 2.20 sec
## large : y2 ~ -1 + year:season + year:season:treatment2:iv1 + year:season:treatment2:iv2 +
##     (1 | village:year) + (1 | village:year:intervalc) + (1 +
##     iv1 + iv2 | year:v.hh)
## small : y2 ~ -1 + YS34 + YS34:treatment2:iv1 + YS34:treatment2:iv2 +
##     (1 | village:year) + (1 | village:year:intervalc) + (1 +
##     iv1 + iv2 | year:v.hh)
##         stat    ndf    ddf F.scaling   p.value
## Ftest 14.193  9.000 11.669   0.89921 5.041e-05
```

```r
# Time trends in 2017-wet and 2017-dry are significantly different

# All year-season combinations are different w.r.t. the time trends per treatment.


# Compare time trends between treatments per year-season combination:
# Within year 2016-dry:
C1 <- rbind(c(0,0,0,0, 1,0,0,0, -1,0,0,0, 0,0,0,0, 0,0,0,0,  0,0,0,0, 0,0,0,0, 0,0,0,0, 0,0,0,0),
            c(0,0,0,0, 1,0,0,0, 0,0,0,0, -1,0,0,0, 0,0,0,0,  0,0,0,0, 0,0,0,0, 0,0,0,0, 0,0,0,0),
            c(0,0,0,0, 1,0,0,0, 0,0,0,0, 0,0,0,0, -1,0,0,0,  0,0,0,0, 0,0,0,0, 0,0,0,0, 0,0,0,0),

            c(0,0,0,0, 0,0,0,0, 0,0,0,0, 0,0,0,0, 0,0,0,0,  1,0,0,0, -1,0,0,0, 0,0,0,0, 0,0,0,0),
            c(0,0,0,0, 0,0,0,0, 0,0,0,0, 0,0,0,0, 0,0,0,0,  1,0,0,0, 0,0,0,0, -1,0,0,0, 0,0,0,0),
            c(0,0,0,0, 0,0,0,0, 0,0,0,0, 0,0,0,0, 0,0,0,0,  1,0,0,0, 0,0,0,0, 0,0,0,0, -1,0,0,0))
linearHypothesis(Rlmero, C1, test="F")
```

```
## Linear hypothesis test
##
## Hypothesis:
## year2016:seasondry:treatment2control:iv1 - year2016:seasondry:treatment2cats:iv1 = 0
## year2016:seasondry:treatment2control:iv1 - year2016:seasondry:treatment2rodenticide:iv1 = 0
## year2016:seasondry:treatment2control:iv1 - year2016:seasondry:treatment2traps:iv1 = 0
## year2016:seasondry:treatment2control:iv2 - year2016:seasondry:treatment2cats:iv2 = 0
## year2016:seasondry:treatment2control:iv2 - year2016:seasondry:treatment2rodenticide:iv2 = 0
## year2016:seasondry:treatment2control:iv2 - year2016:seasondry:treatment2traps:iv2 = 0
##
## Model 1: restricted model
## Model 2: y2 ~ -1 + year:season + year:season:treatment2:iv1 + year:season:treatment2:iv2 +
##     (1 | village:year) + (1 | village:year:intervalc) + (1 +
##     iv1 + iv2 | year:v.hh)
##
##   Res.Df Df      F Pr(>F)
## 1 46.104
## 2 40.104  6 0.7733 0.5955
```

```r
# Time trend differences between treatments in 2016-dry

# Within year 2017-dry:
C2 <- rbind(c(0,0,0,0, 0,1,0,0, 0,-1,0,0, 0,0,0,0, 0,0,0,0,  0,0,0,0, 0,0,0,0, 0,0,0,0, 0,0,0,0),
            c(0,0,0,0, 0,1,0,0, 0,0,0,0, 0,-1,0,0, 0,0,0,0,  0,0,0,0, 0,0,0,0, 0,0,0,0, 0,0,0,0),
            c(0,0,0,0, 0,1,0,0, 0,0,0,0, 0,0,0,0, 0,-1,0,0,  0,0,0,0, 0,0,0,0, 0,0,0,0, 0,0,0,0),
```

```
           c(0,0,0,0, 0,0,0,0, 0,0,0,0, 0,0,0,0, 0,0,0,0,  0,1,0,0, 0,-1,0,0, 0,0,0,0, 0,0,0,0),
           c(0,0,0,0, 0,0,0,0, 0,0,0,0, 0,0,0,0, 0,0,0,0,  0,1,0,0, 0,0,0,0, 0,-1,0,0, 0,0,0,0),
           c(0,0,0,0, 0,0,0,0, 0,0,0,0, 0,0,0,0, 0,0,0,0,  0,1,0,0, 0,0,0,0, 0,0,0,0, 0,-1,0,0))
linearHypothesis(Rlmero, C2, test="F")
```

```
## Linear hypothesis test
##
## Hypothesis:
## year2017:seasondry:treatment2control:iv1 - year2017:seasondry:treatment2cats:iv1 = 0
## year2017:seasondry:treatment2control:iv1 - year2017:seasondry:treatment2rodenticide:iv1 = 0
## year2017:seasondry:treatment2control:iv1 - year2017:seasondry:treatment2traps:iv1 = 0
## year2017:seasondry:treatment2control:iv2 - year2017:seasondry:treatment2cats:iv2 = 0
## year2017:seasondry:treatment2control:iv2 - year2017:seasondry:treatment2rodenticide:iv2 = 0
## year2017:seasondry:treatment2control:iv2 - year2017:seasondry:treatment2traps:iv2 = 0
##
## Model 1: restricted model
## Model 2: y2 ~ -1 + year:season + year:season:treatment2:iv1 + year:season:treatment2:iv2 +
##     (1 | village:year) + (1 | village:year:intervalc) + (1 +
##     iv1 + iv2 | year:v.hh)
##
##   Res.Df Df      F Pr(>F)
## 1 20.141
## 2 14.141  6 0.1506 0.9858
```

```
# No time trend differences between treatments in 2017-dry

# Within year 2016-wet:
C3 <- rbind(c(0,0,0,0, 0,0,1,0, 0,0,-1,0, 0,0,0,0, 0,0,0,0,  0,0,0,0, 0,0,0,0, 0,0,0,0, 0,0,0,0),
            c(0,0,0,0, 0,0,1,0, 0,0,0,0, 0,0,-1,0, 0,0,0,0,  0,0,0,0, 0,0,0,0, 0,0,0,0, 0,0,0,0),
            c(0,0,0,0, 0,0,1,0, 0,0,0,0, 0,0,0,0, 0,0,-1,0,  0,0,0,0, 0,0,0,0, 0,0,0,0, 0,0,0,0),

            c(0,0,0,0, 0,0,0,0, 0,0,0,0, 0,0,0,0, 0,0,0,0,  0,0,1,0, 0,0,-1,0, 0,0,0,0, 0,0,0,0),
            c(0,0,0,0, 0,0,0,0, 0,0,0,0, 0,0,0,0, 0,0,0,0,  0,0,1,0, 0,0,0,0, 0,0,-1,0, 0,0,0,0),
            c(0,0,0,0, 0,0,0,0, 0,0,0,0, 0,0,0,0, 0,0,0,0,  0,0,1,0, 0,0,0,0, 0,0,0,0, 0,0,-1,0))
linearHypothesis(Rlmero, C3, test="F")
```

```
## Linear hypothesis test
##
## Hypothesis:
## year2016:seasonwet:treatment2control:iv1 - year2016:seasonwet:treatment2cats:iv1 = 0
## year2016:seasonwet:treatment2control:iv1 - year2016:seasonwet:treatment2rodenticide:iv1 = 0
## year2016:seasonwet:treatment2control:iv1 - year2016:seasonwet:treatment2traps:iv1 = 0
## year2016:seasonwet:treatment2control:iv2 - year2016:seasonwet:treatment2cats:iv2 = 0
## year2016:seasonwet:treatment2control:iv2 - year2016:seasonwet:treatment2rodenticide:iv2 = 0
## year2016:seasonwet:treatment2control:iv2 - year2016:seasonwet:treatment2traps:iv2 = 0
##
## Model 1: restricted model
## Model 2: y2 ~ -1 + year:season + year:season:treatment2:iv1 + year:season:treatment2:iv2 +
##     (1 | village:year) + (1 | village:year:intervalc) + (1 +
##     iv1 + iv2 | year:v.hh)
##
##    Res.Df Df     F   Pr(>F)
## 1 14.9444
## 2  8.9444  6 9.705 0.001707
```

```
# No time trend differences between treatments in 2016-wet

# Within year 2017-wet:
C4 <- rbind(c(0,0,0,0, 0,0,0,1, 0,0,0,-1, 0,0,0,0, 0,0,0,0,  0,0,0,0, 0,0,0,0, 0,0,0,0, 0,0,0,0),
            c(0,0,0,0, 0,0,0,1, 0,0,0,0, 0,0,0,-1, 0,0,0,0,  0,0,0,0, 0,0,0,0, 0,0,0,0, 0,0,0,0),
            c(0,0,0,0, 0,0,0,1, 0,0,0,0, 0,0,0,0, 0,0,0,-1,  0,0,0,0, 0,0,0,0, 0,0,0,0, 0,0,0,0),
```

```r
           c(0,0,0,0, 0,0,0,0, 0,0,0,0, 0,0,0,0, 0,0,0,0,  0,0,0,1, 0,0,0,-1, 0,0,0,0, 0,0,0,0),
           c(0,0,0,0, 0,0,0,0, 0,0,0,0, 0,0,0,0, 0,0,0,0,  0,0,0,1, 0,0,0,0, 0,0,0,-1, 0,0,0,0),
           c(0,0,0,0, 0,0,0,0, 0,0,0,0, 0,0,0,0, 0,0,0,0,  0,0,0,1, 0,0,0,0, 0,0,0,0, 0,0,0,-1))
linearHypothesis(Rlmero, C4, test="F")
```

```
## Linear hypothesis test
##
## Hypothesis:
## year2017:seasonwet:treatment2control:iv1 - year2017:seasonwet:treatment2cats:iv1 = 0
## year2017:seasonwet:treatment2control:iv1 - year2017:seasonwet:treatment2rodenticide:iv1 = 0
## year2017:seasonwet:treatment2control:iv1 - year2017:seasonwet:treatment2traps:iv1 = 0
## year2017:seasonwet:treatment2control:iv2 - year2017:seasonwet:treatment2cats:iv2 = 0
## year2017:seasonwet:treatment2control:iv2 - year2017:seasonwet:treatment2rodenticide:iv2 = 0
## year2017:seasonwet:treatment2control:iv2 - year2017:seasonwet:treatment2traps:iv2 = 0
##
## Model 1: restricted model
## Model 2: y2 ~ -1 + year:season + year:season:treatment2:iv1 + year:season:treatment2:iv2 +
##     (1 | village:year) + (1 | village:year:intervalc) + (1 +
##     iv1 + iv2 | year:v.hh)
##
##   Res.Df Df      F Pr(>F)
## 1 20.116
## 2 14.116  6 0.5361 0.7724
```

```r
# No time trend differences between treatments in 2017-wet

# Only within year 2016-wet do we find significant differences among treatment trends.
```

## Data on rice loss per day: Testing treatment effects at different time points

Selecting only the year-season combinations where overall differences among treatment time trends were observed, we continue by comparing individual treatments pairwise at the specific time points where observations were taken. The Tukey method for pairwise comparison of four treatments is applied.

```r
# 14 days after start of treatment:
RG1 <- ref_grid(Rlmero, at=list(year="2016", season="wet", iv1=(14/sd.iv), iv2=(14/sd.iv)^2))
pairs(RG1)
```

```
##  contrast
##  2016,wet,control,0.534616027269727,0.285814296613665 - 2016,wet,cats,0.534616027269727,0.285814296613665
##  2016,wet,control,0.534616027269727,0.285814296613665 - 2016,wet,rodenticide,0.534616027269727,0.2858142966
##  2016,wet,control,0.534616027269727,0.285814296613665 - 2016,wet,traps,0.534616027269727,0.285814296613665
##  2016,wet,cats,0.534616027269727,0.285814296613665 - 2016,wet,rodenticide,0.534616027269727,0.2858142966136
##  2016,wet,cats,0.534616027269727,0.285814296613665 - 2016,wet,traps,0.534616027269727,0.285814296613665
##  2016,wet,rodenticide,0.534616027269727,0.285814296613665 - 2016,wet,traps,0.534616027269727,0.285814296613
##  estimate     SE   df t.ratio p.value
##   -0.1986 0.0599 25.3 -3.315  0.0138
##   -0.2224 0.0599 25.3 -3.713  0.0053
##   -0.2534 0.0599 25.3 -4.231  0.0014
##   -0.0238 0.0723 19.7 -0.330  0.9873
##   -0.0549 0.0723 19.7 -0.759  0.8719
##   -0.0310 0.0723 19.7 -0.429  0.9728
##
## P value adjustment: tukey method for comparing a family of 4 estimates
```

```r
CLD(RG1, Letters=letters)
```

```
##  year season treatment2    iv1   iv2 prediction     SE   df .group
##  2016 wet    control     0.535 0.286       1.24 0.0415 13.6  a
##  2016 wet    cats        0.535 0.286       1.44 0.0503 13.2   b
##  2016 wet    rodenticide 0.535 0.286       1.47 0.0503 13.2   b
```

```
##  2016 wet    traps       0.535 0.286       1.50 0.0503 13.2   b
##
## Degrees-of-freedom method: kenward-roger
## P value adjustment: tukey method for comparing a family of 4 estimates
## significance level used: alpha = 0.05
```

```r
# 28 days after start of treatment:
RG2 <- ref_grid(Rlmero, at=list(year="2016", season="wet", iv1=(28/sd.iv), iv2=(28/sd.iv)^2))
pairs(RG2)
```

```
##  contrast
##  2016,wet,control,1.06923205453945,1.14325718645466 - 2016,wet,cats,1.06923205453945,1.14325718645466
##  2016,wet,control,1.06923205453945,1.14325718645466 - 2016,wet,rodenticide,1.06923205453945,1.14325718645466
##  2016,wet,control,1.06923205453945,1.14325718645466 - 2016,wet,traps,1.06923205453945,1.14325718645466
##  2016,wet,cats,1.06923205453945,1.14325718645466 - 2016,wet,rodenticide,1.06923205453945,1.14325718645466
##  2016,wet,cats,1.06923205453945,1.14325718645466 - 2016,wet,traps,1.06923205453945,1.14325718645466
##  2016,wet,rodenticide,1.06923205453945,1.14325718645466 - 2016,wet,traps,1.06923205453945,1.14325718645466
##  estimate     SE   df t.ratio p.value
##  -0.26097 0.0899 13.4 -2.901  0.0521
##  -0.26211 0.0899 13.4 -2.914  0.0509
##  -0.30280 0.0899 13.4 -3.366  0.0223
##  -0.00113 0.1062 11.2 -0.011  1.0000
##  -0.04183 0.1062 11.2 -0.394  0.9783
##  -0.04069 0.1062 11.2 -0.383  0.9799
##
## P value adjustment: tukey method for comparing a family of 4 estimates
```

```r
CLD(RG2, Letters=letters)
```

```
##  year season treatment2   iv1  iv2 prediction     SE   df .group
##  2016 wet    control     1.07 1.14       1.16 0.0466  8.0  a
##  2016 wet    cats        1.07 1.14       1.42 0.0720 11.4  ab
##  2016 wet    rodenticide 1.07 1.14       1.42 0.0720 11.4  ab
##  2016 wet    traps       1.07 1.14       1.46 0.0720 11.4   b
##
## Degrees-of-freedom method: kenward-roger
## P value adjustment: tukey method for comparing a family of 4 estimates
## significance level used: alpha = 0.05
```

```r
# 42 days after start of treatment:
RG3 <- ref_grid(Rlmero, at=list(year="2016", season="wet", iv1=(42/sd.iv), iv2=(42/sd.iv)^2))
pairs(RG3)
```

```
##  contrast
##  2016,wet,control,1.60384808180918,2.57232866952299 - 2016,wet,cats,1.60384808180918,2.57232866952299
##  2016,wet,control,1.60384808180918,2.57232866952299 - 2016,wet,rodenticide,1.60384808180918,2.57232866952299
##  2016,wet,control,1.60384808180918,2.57232866952299 - 2016,wet,traps,1.60384808180918,2.57232866952299
##  2016,wet,cats,1.60384808180918,2.57232866952299 - 2016,wet,rodenticide,1.60384808180918,2.57232866952299
##  2016,wet,cats,1.60384808180918,2.57232866952299 - 2016,wet,traps,1.60384808180918,2.57232866952299
##  2016,wet,rodenticide,1.60384808180918,2.57232866952299 - 2016,wet,traps,1.60384808180918,2.57232866952299
##  estimate     SE   df t.ratio p.value
##   -0.1873 0.0971 5.27 -1.929  0.3196
##   -0.1191 0.0971 5.27 -1.227  0.6369
##   -0.1481 0.0971 5.27 -1.526  0.4856
##    0.0681 0.1077 4.64  0.633  0.9168
##    0.0391 0.1077 4.64  0.363  0.9817
##   -0.0290 0.1077 4.64 -0.269  0.9923
##
## P value adjustment: tukey method for comparing a family of 4 estimates
```

```r
CLD(RG3, Letters=letters)
```

```
##  year season treatment2  iv1  iv2 prediction     SE   df .group
```

```
##  2016 wet    control     1.6 2.57       1.15 0.0524 4.24  a
##  2016 wet    rodenticide 1.6 2.57       1.27 0.0726 5.31  a
##  2016 wet    traps       1.6 2.57       1.30 0.0726 5.31  a
##  2016 wet    cats        1.6 2.57       1.34 0.0726 5.31  a
##
## Degrees-of-freedom method: kenward-roger
## P value adjustment: tukey method for comparing a family of 4 estimates
## significance level used: alpha = 0.05
```

```
# 56 days after start of treatment:
RG4 <- ref_grid(Rlmero, at=list(year="2016", season="wet", iv1=(56/sd.iv), iv2=(56/sd.iv)^2))
pairs(RG4)
```

```
##  contrast
##  2016,wet,control,2.13846410907891,4.57302874581864 - 2016,wet,cats,2.13846410907891,4.57302874581864
##  2016,wet,control,2.13846410907891,4.57302874581864 - 2016,wet,rodenticide,2.13846410907891,4.5730287458186
##  2016,wet,control,2.13846410907891,4.57302874581864 - 2016,wet,traps,2.13846410907891,4.57302874581864
##  2016,wet,cats,2.13846410907891,4.57302874581864 - 2016,wet,rodenticide,2.13846410907891,4.57302874581864
##  2016,wet,cats,2.13846410907891,4.57302874581864 - 2016,wet,traps,2.13846410907891,4.57302874581864
##  2016,wet,rodenticide,2.13846410907891,4.57302874581864 - 2016,wet,traps,2.13846410907891,4.57302874581864
##  estimate    SE   df t.ratio p.value
##   0.02260 0.103 5.25 0.220   0.9958
##   0.20657 0.103 5.25 2.011   0.2924
##   0.21059 0.103 5.25 2.051   0.2801
##   0.18397 0.101 4.91 1.820   0.3651
##   0.18799 0.101 4.91 1.860   0.3503
##   0.00402 0.101 4.91 0.040   1.0000
##
## P value adjustment: tukey method for comparing a family of 4 estimates
```

```
CLD(RG4, Letters=letters)
```

```
##  year season treatment2   iv1  iv2 prediction     SE   df .group
##  2016 wet    traps       2.14 4.57       1.03 0.0679 5.30  a
##  2016 wet    rodenticide 2.14 4.57       1.03 0.0679 5.30  a
##  2016 wet    cats        2.14 4.57       1.22 0.0679 5.30  a
##  2016 wet    control     2.14 4.57       1.24 0.0677 6.31  a
##
## Degrees-of-freedom method: kenward-roger
## P value adjustment: tukey method for comparing a family of 4 estimates
## significance level used: alpha = 0.05
```

```
# 67 days after start of treatment (final measurement day)
RG5 <- ref_grid(Rlmero, at=list(year="2016", season="wet", iv1=(67/sd.iv), iv2=(67/sd.iv)^2))
pairs(RG5)
```

```
##  contrast
##  2016,wet,control,2.55851955907655,6.54602233417726 - 2016,wet,cats,2.55851955907655,6.54602233417726
##  2016,wet,control,2.55851955907655,6.54602233417726 - 2016,wet,rodenticide,2.55851955907655,6.5460223341772
##  2016,wet,control,2.55851955907655,6.54602233417726 - 2016,wet,traps,2.55851955907655,6.54602233417726
##  2016,wet,cats,2.55851955907655,6.54602233417726 - 2016,wet,rodenticide,2.55851955907655,6.54602233417726
##  2016,wet,cats,2.55851955907655,6.54602233417726 - 2016,wet,traps,2.55851955907655,6.54602233417726
##  2016,wet,rodenticide,2.55851955907655,6.54602233417726 - 2016,wet,traps,2.55851955907655,6.54602233417726
##  estimate    SE   df t.ratio p.value
##     0.283 0.133 29.7 2.134   0.1658
##     0.591 0.133 29.7 4.454   0.0006
##     0.636 0.133 29.7 4.793   0.0002
##     0.308 0.131 49.3 2.351   0.1004
##     0.353 0.131 49.3 2.695   0.0459
##     0.045 0.131 49.3 0.344   0.9859
##
## P value adjustment: tukey method for comparing a family of 4 estimates
```

```r
CLD(RG5, Letters=letters)
```

```
##  year season treatment2   iv1  iv2 prediction     SE   df .group
##  2016 wet    traps       2.56 6.55      0.729 0.0906 46.9  a
##  2016 wet    rodenticide 2.56 6.55      0.774 0.0906 46.9  ab
##  2016 wet    cats        2.56 6.55      1.081 0.0906 46.9   bc
##  2016 wet    control     2.56 6.55      1.364 0.0903 15.7    c
##
## Degrees-of-freedom method: kenward-roger
## P value adjustment: tukey method for comparing a family of 4 estimates
## significance level used: alpha = 0.05
```

### Data on rice loss per day: 2016-wet season plot of data and fitted model

```r
R2016W <- R[R$year==2016 & R$season=="wet",]

coefs <- fixef(Rlmero)

b0    <- coefs[1:4]

b1.co <- coefs[5:8]
b2.co <- coefs[21:24]

b1.ca <- coefs[ 9:12]
b2.ca <- coefs[25:28]

b1.ro <- coefs[13:16]
b2.ro <- coefs[29:32]

b1.tr <- coefs[17:20]
b2.tr <- coefs[33:36]

iv <- seq(-28,67,1)/sd.iv
iv2 <- seq(0,67,1)/sd.iv

eta.co <- b0[3] + b1.co[3]*iv  + b2.co[3]*iv^2
eta.ca <- b0[3] + b1.ca[3]*iv2 + b2.ca[3]*iv2^2
eta.ro <- b0[3] + b1.ro[3]*iv2 + b2.ro[3]*iv2^2
eta.tr <- b0[3] + b1.tr[3]*iv2 + b2.tr[3]*iv2^2

yhat.co <- eta.co
yhat.ca <- eta.ca
yhat.ro <- eta.ro
yhat.tr <- eta.tr

V <- vcov(Rlmero)

C.co <- matrix(rep(0,length(iv)*36), ncol=36)
C.co[,c(3,7,23)] <- cbind(1,iv,iv^2)

var.yhat.co <- diag(C.co %*% V %*% t(C.co))
se.yhat.co <- sqrt(var.yhat.co)
lwr.co <- yhat.co - 1.96*se.yhat.co
upr.co <- yhat.co + 1.96*se.yhat.co

df.ctrl <- data.frame(iv=iv*sd.iv, yhat.co=yhat.co, lwr.co=lwr.co, upr.co=upr.co)

C.ca <- matrix(rep(0,length(iv2)*36), ncol=36)
C.ca[,c(3,11,27)] <- cbind(1,iv2,iv2^2)
```

```
var.yhat.ca <- diag(C.ca %*% V %*% t(C.ca))
se.yhat.ca <- sqrt(var.yhat.ca)
lwr.ca <- yhat.ca - 1.96*se.yhat.ca
upr.ca <- yhat.ca + 1.96*se.yhat.ca

C.ro <- matrix(rep(0,length(iv2)*36), ncol=36)
C.ro[,c(3,15,31)] <- cbind(1,iv2,iv2^2)
var.yhat.ro <- diag(C.ro %*% V %*% t(C.ro))
se.yhat.ro <- sqrt(var.yhat.ro)
lwr.ro <- yhat.ro - 1.96*se.yhat.ro
upr.ro <- yhat.ro + 1.96*se.yhat.ro

C.tr <- matrix(rep(0,length(iv2)*36), ncol=36)
C.tr[,c(3,19,35)] <- cbind(1,iv2,iv2^2)
var.yhat.tr <- diag(C.tr %*% V %*% t(C.tr))
se.yhat.tr <- sqrt(var.yhat.tr)
lwr.tr <- yhat.tr - 1.96*se.yhat.tr
upr.tr <- yhat.tr + 1.96*se.yhat.tr

df.other <- data.frame(iv2=iv2*sd.iv, yhat.ca=yhat.ca, lwr=lwr.ca, upr=upr.ca,
                       yhat.ro=yhat.ro, lwr=lwr.ro, upr=upr.ro,
                       yhat.tr=yhat.tr, lwr=lwr.tr, upr=upr.tr)

man.col <- c("#F8766D", "#7CAE00", "#C77CFF", "#00BFC4")


# plot 95% confidence bands and points instead of lines
p <- ggplot(data=R2016W, aes(x=intervaldays, y=y2)) +
  labs(x = "days since start treatment", y = "rice loss per day (g)") + theme_bw()+ theme(panel.grid.minor = e
  geom_point(aes(colour=treatment2, group=v.hh), size=1, alpha=0.75) +
  scale_y_continuous(breaks=c(0.0414,0.3010,0.7782,1.0414,1.7076,2.0043), labels=c("0.1","1","5","10","50","10
  scale_x_continuous(breaks=c(-28,-14,0,14,28,42,56,70), labels=c("-28","-14","0","14","28","42","56","70")) +
  guides(colour = guide_legend(override.aes = list(alpha = 1), title="treatment")) +
  scale_colour_manual(values=man.col) +
  geom_ribbon(data=df.ctrl, mapping=aes(x=iv, y=yhat.co, ymin = lwr.co, ymax = upr.co, group=1),
              fill=man.col[1], alpha = .2) +
  geom_line(data=df.ctrl,  mapping=aes(x=iv, y=yhat.co, group=1),  size=1.1, colour=man.col[1]) +

  geom_ribbon(data=df.other, mapping=aes(x=iv2, y=yhat.ca, ymin = lwr.ca, ymax = upr.ca, group=1),
              fill=man.col[2], alpha = .2) +
  geom_line(data=df.other, mapping=aes(x=iv2, y=yhat.ca, group=1), size=1.1, colour=man.col[2]) +

  geom_ribbon(data=df.other, mapping=aes(x=iv2, y=yhat.ro, ymin = lwr.ro, ymax = upr.ro, group=1),
              fill=man.col[3], alpha = .2) +
  geom_line(data=df.other, mapping=aes(x=iv2, y=yhat.ro, group=1), size=1.1, colour=man.col[3]) +

  geom_ribbon(data=df.other, mapping=aes(x=iv2, y=yhat.tr, ymin = lwr.tr, ymax = upr.tr, group=1),
              fill=man.col[4], alpha = .2) +
  geom_line(data=df.other, mapping=aes(x=iv2, y=yhat.tr, group=1), size=1.1, colour=man.col[4])
p
```

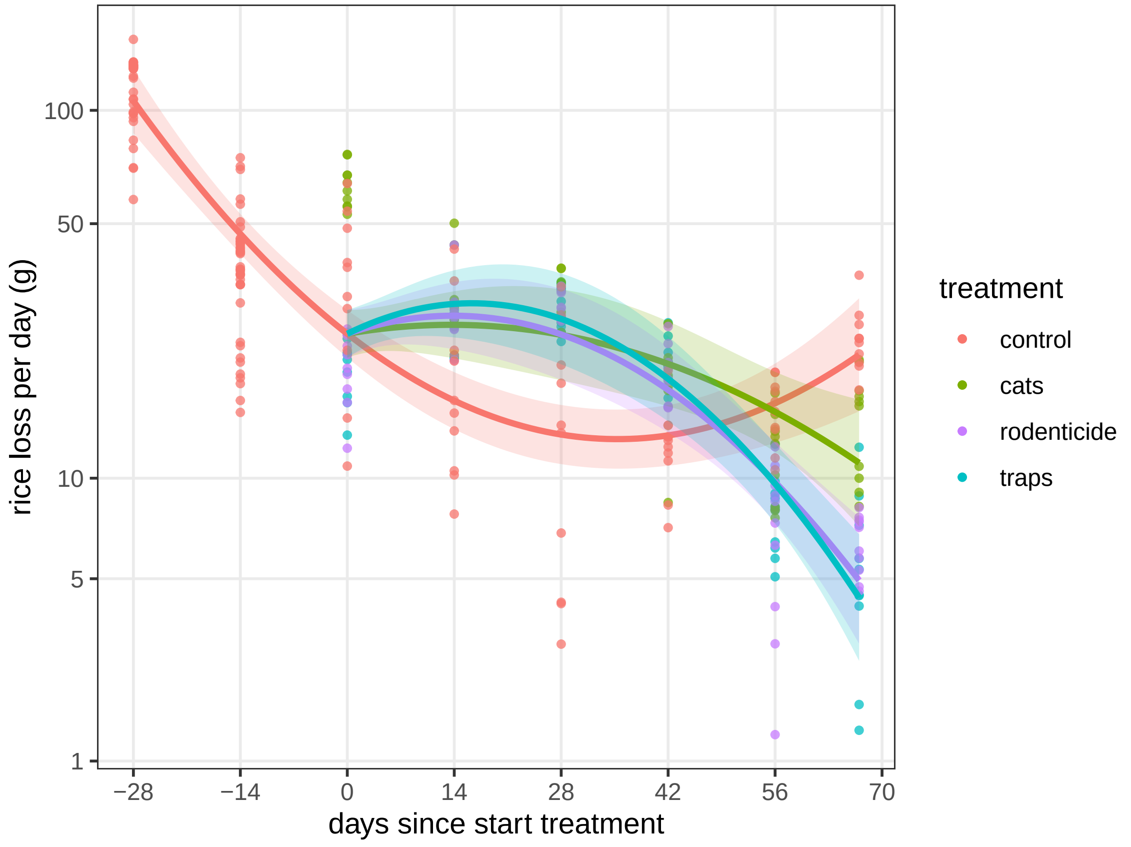


```
# plot 95% confidence bands and points instead of lines
p <- ggplot(data=R2016W, aes(x=intervaldays, y=y2)) +
  labs(x = "days since start treatment", y = "rice loss per day (g)") + theme_bw()+ theme(panel.grid.minor = e
  geom_point(aes(colour=treatment2, group=v.hh), size=1, alpha=0.75) +
  scale_y_continuous(breaks=c(0.0414,0.3010,0.7782,1.0414,1.7076,2.0043), labels=c("0.1","1","5","10","50","10
  scale_x_continuous(breaks=c(-28,-14,0,14,28,42,56,70), labels=c("-28","-14","0","14","28","42","56","70")) +
  guides(colour = guide_legend(override.aes = list(alpha = 1), title="treatment")) +
  scale_colour_manual(values=man.col) +
  geom_ribbon(data=df.ctrl, mapping=aes(x=iv, y=yhat.co, ymin = lwr.co, ymax = upr.co, group=1),
            fill=man.col[1], alpha = .2, colour=man.col[1], size=0.2) +
  geom_line(data=df.ctrl,  mapping=aes(x=iv, y=yhat.co, group=1),  size=1.1, colour=man.col[1]) +

  geom_ribbon(data=df.other, mapping=aes(x=iv2, y=yhat.ca, ymin = lwr.ca, ymax = upr.ca, group=1),
            fill=man.col[2], alpha = .2, colour=man.col[2], size=0.2) +
  geom_line(data=df.other, mapping=aes(x=iv2, y=yhat.ca, group=1), size=1.1, colour=man.col[2]) +

  geom_ribbon(data=df.other, mapping=aes(x=iv2, y=yhat.ro, ymin = lwr.ro, ymax = upr.ro, group=1),
            fill=man.col[3], alpha = .2, colour=man.col[3], size=0.2) +
  geom_line(data=df.other, mapping=aes(x=iv2, y=yhat.ro, group=1), size=1.1, colour=man.col[3]) +

  geom_ribbon(data=df.other, mapping=aes(x=iv2, y=yhat.tr, ymin = lwr.tr, ymax = upr.tr, group=1),
            fill=man.col[4], alpha = .2, colour=man.col[4], size=0.2) +
  geom_line(data=df.other, mapping=aes(x=iv2, y=yhat.tr, group=1), size=1.1, colour=man.col[4])
p
```

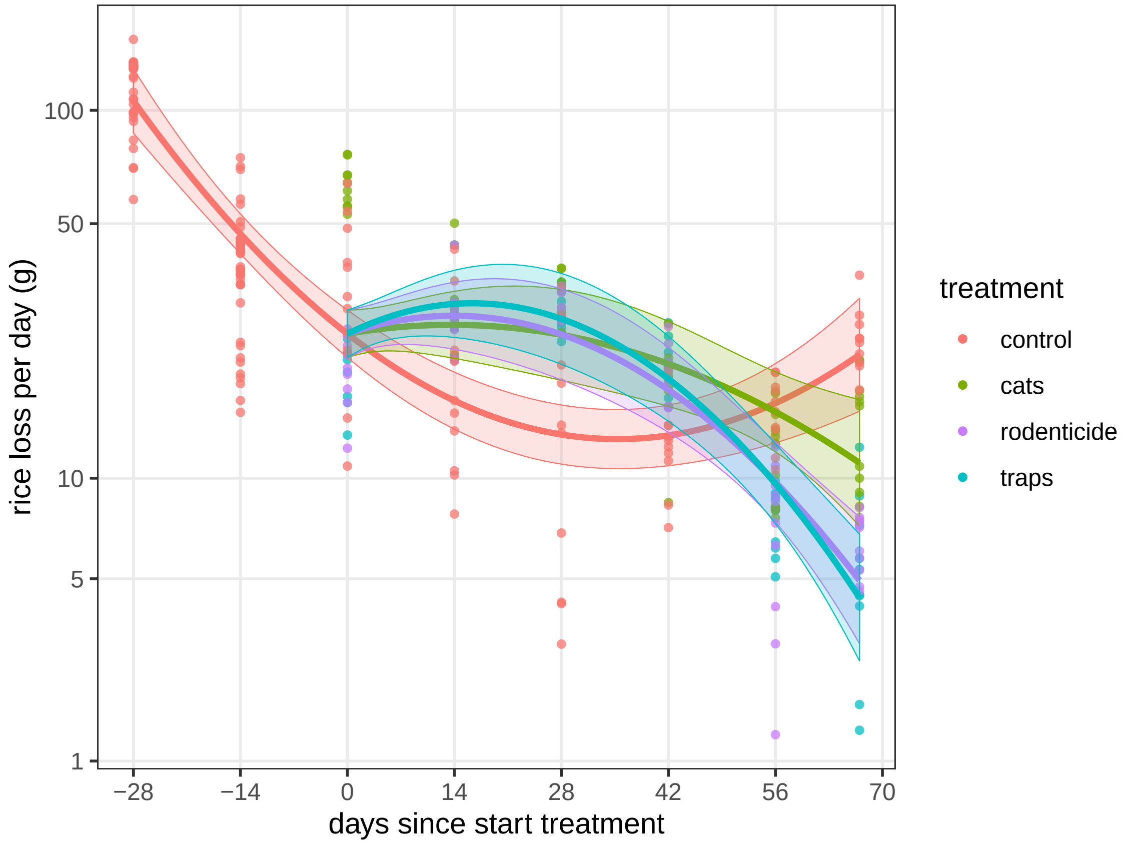
rice loss per day (g)
100
50
10
5
1
−28
−14
0
14
28
42
56
70
days since start treatment
treatment
control
cats
rodenticide
traps

```
cat("\n")
```

## Analyze data on fraction of cells of tracking tiles tripped upon per day

The response variable is the number of cells out of 16 per tracking tile, tripped upon over night. Per household two tiles were used, one at floor level, one at ceiling level. Per tile measurements were taken on two consecutive days after the days of the rice measurements.

### Data on tracking tiles: overview plot

```
setwd("C:/MyData/OneDrive - WageningenUR/Data/Inge Krijger/final/Tiles")
T <- read.csv("Tiles.csv")

T$treatment2 <- relevel(T$treatment2, ref="control")

T$year <- factor(T$year)
T$intervalc <- factor(T$interval2)

sd.iv <- sd(T$intervaldays)
T$iv1 <- (T$intervaldays)/(sd(T$intervaldays))
T$iv2 <- T$iv1*T$iv1
T$iv3 <- T$iv1*T$iv1*T$iv1

T$YS <- factor(paste(T$year, T$season, sep="."), levels=c("2016.wet","2016.dry","2017.wet","2017.dry"))
# Note: order of levels of T$YS is now: "2016.wet" "2016.dry" "2017.wet" "2017.dry"

head(T)
```

```
##   interval village year hh     date tile location kt pct.kt species
## 1        1       A 2016  1 6-6-2016    1        F 12     75   MM,RR
## 2        1       A 2016  1 6-6-2016    2        C  8     50   MM,RR
## 3        1       A 2016  2 6-6-2016    1        F 11  68.75  MM,SUN
## 4        1       A 2016  2 6-6-2016    2        C  6   37.5   MM,RR
## 5        1       A 2016  3 6-6-2016    1        F 10   62.5  RR,SUN
## 6        1       A 2016  3 6-6-2016    2        C 12     75   RR,MM
##   interval2 intervaldays treatment treatment2 season       YS twodayperiod
## 1        -3          -35     traps    control    wet 2016.wet            1
## 2        -3          -35     traps    control    wet 2016.wet            1
## 3        -3          -35     traps    control    wet 2016.wet            1
## 4        -3          -35     traps    control    wet 2016.wet            1
## 5        -3          -35     traps    control    wet 2016.wet            1
## 6        -3          -35     traps    control    wet 2016.wet            1
##    date.nw nt v.hh intervalc       iv1      iv2      iv3
## 1 7-6-2016 16  A.1        -3 -1.180698 1.394048 -1.64595
## 2 7-6-2016 16  A.1        -3 -1.180698 1.394048 -1.64595
## 3 7-6-2016 16  A.2        -3 -1.180698 1.394048 -1.64595
## 4 7-6-2016 16  A.2        -3 -1.180698 1.394048 -1.64595
## 5 7-6-2016 16  A.3        -3 -1.180698 1.394048 -1.64595
## 6 7-6-2016 16  A.3        -3 -1.180698 1.394048 -1.64595
```

```
o <- order(T$year, T$season, T$treatment, T$hh, T$date.nw, T$location)

# Take sum over two locations (floor and ceiling) and two consecutive days per household
Ta <- summaryBy(kt + nt + intervaldays ~ village + year + season + YS + treatment2 +
                  interval + v.hh + date, data=T, FUN=sum, na.rm=TRUE )

Ta$f <- Ta$kt/Ta$nt
Ta$intervaldays <- Ta$intervaldays.sum/4


man.col <- c("#F8766D", "#7CAE00", "#C77CFF", "#00BFC4")

# Overview plot of fraction
```

```
ggplot(data=Ta, aes(x=intervaldays, y=f, colour=treatment2, group=v.hh)) + theme_bw()+
  theme(panel.grid.minor = element_blank()) +
  geom_line(size=0.3, alpha=0.35) + facet_wrap(~YS) +
  scale_x_continuous(breaks=c(-28,-14,0,14,28,42,56,70), labels=c("-28","-14","0","14","28","42","56","70")) +
  labs(x = "days since start treatment", y = "fraction of cells of tiles tripped upon") +
  guides(colour = guide_legend(override.aes = list(alpha = 1), title="treatment")) +
  scale_color_manual(values=man.col)
```

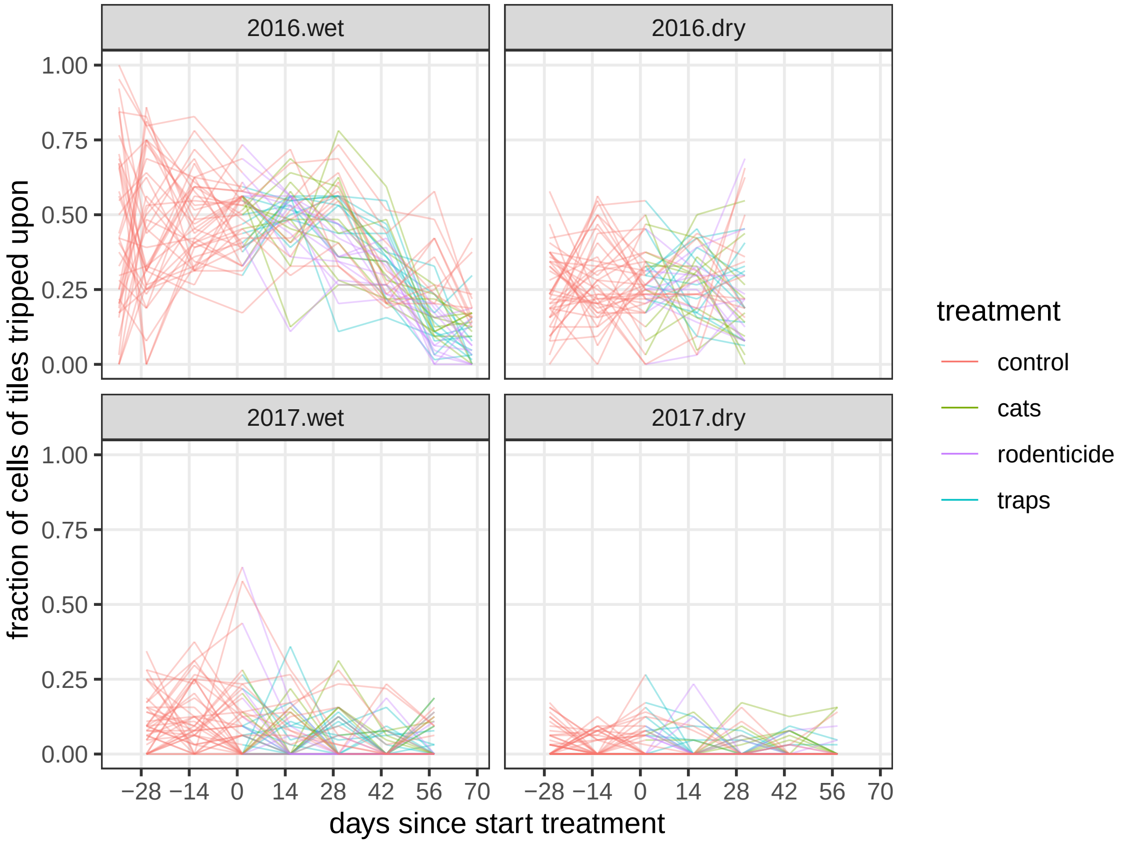


## Data on tracking tiles: generalized linear mixed model analysis of fraction tripped-upon tile parts

We fit a generalized linear mixed model for the number of cells tripped upon over night, assuming a binomial distribution. The fixed and random parts of the model are largely the same as for rice loss. In the fixed part an extra effect for location (floor vs ceiling) is added. In the random part an observation level random effect is added to handle binomial overdispersion, and an extra random effect to handle variability among the two consecutive days that measurements on tiles were taken.

```
#Tglmero <- glmer(cbind(kt,nt-kt) ~
#              -1 + location + year:season + year:season:treatment2:iv1 + year:season:treatment2:iv2
#               + (1 | village:year) + (1 | village:year:intervalc) + (1 | village:year:v.hh:date.nw)
#               + (1 | village:year:v.hh:date.nw:location) + (1 + iv1 + iv2 | year:v.hh),
#               family=binomial(link=logit), data=T,
#               control = glmerControl(optimizer="nloptwrap", optCtrl = list(maxfun = 1000000)))
#saveRDS(Tglmero, "C:/MyData/OneDrive - WageningenUR/Data/Inge Krijger/final/Tiles/Tglmero")
Tglmero <- readRDS("C:/MyData/OneDrive - WageningenUR/Data/Inge Krijger/final/Tiles/Tglmero")

logLik(Tglmero)
```

```
## 'log Lik.' -7804.645 (df=47)
```

```
#Tglmero.iv3 <- glmer(cbind(kt,nt-kt) ~
#              -1 + location + year:season
#               + year:season:treatment2:iv1 + year:season:treatment2:iv2 + year:season:treatment2:iv3
#               + (1 | village:year) + (1 | village:year:intervalc) + (1 | village:year:v.hh:date.nw)
```

```r
#               + (1 | village:year:v.hh:date.nw:location) + (1 + iv1 + iv2 + iv3| year:v.hh),
#               family=binomial(link=logit), data=T,
#               control = glmerControl(optimizer ="nloptwrap", optCtrl = list(maxfun = 1000000)))
#saveRDS(Tglmero.iv3, "C:/MyData/OneDrive - WageningenUR/Data/Inge Krijger/final/Tiles/Tglmeroiv3")
Tglmero.iv3 <- readRDS("C:/MyData/OneDrive - WageningenUR/Data/Inge Krijger/final/Tiles/Tglmeroiv3")

logLik(Tglmero.iv3)
```

```
## 'log Lik.' -7792.169 (df=67)
```

```r
anova(Tglmero, Tglmero.iv3)
```

```
## Data: T
## Models:
## Tglmero: cbind(kt, nt - kt) ~ -1 + location + year:season + year:season:treatment2:iv1 +
## Tglmero:     year:season:treatment2:iv2 + (1 | village:year) + (1 | village:year:intervalc) +
## Tglmero:     (1 | village:year:v.hh:date.nw) + (1 | village:year:v.hh:date.nw:location) +
## Tglmero:     (1 + iv1 + iv2 | year:v.hh)
## Tglmero.iv3: cbind(kt, nt - kt) ~ -1 + location + year:season + year:season:treatment2:iv1 +
## Tglmero.iv3:     year:season:treatment2:iv2 + year:season:treatment2:iv3 +
## Tglmero.iv3:     (1 | village:year) + (1 | village:year:intervalc) + (1 |
## Tglmero.iv3:     village:year:v.hh:date.nw) + (1 | village:year:v.hh:date.nw:location) +
## Tglmero.iv3:     (1 + iv1 + iv2 + iv3 | year:v.hh)
##             Df   AIC   BIC  logLik deviance  Chisq Chi Df Pr(>Chisq)
## Tglmero     47 15703 16004 -7804.6    15609
## Tglmero.iv3 67 15718 16148 -7792.2    15584 24.952     20     0.2033
```

```r
# Model with cubic terms (both in fixed and random part) not better

# We continue with the quadratic model
summary(Tglmero)
```

```
## Generalized linear mixed model fit by maximum likelihood (Laplace
##   Approximation) [glmerMod]
##  Family: binomial  ( logit )
## Formula:
## cbind(kt, nt - kt) ~ -1 + location + year:season + year:season:treatment2:iv1 +
##     year:season:treatment2:iv2 + (1 | village:year) + (1 | village:year:intervalc) +
##     (1 | village:year:v.hh:date.nw) + (1 | village:year:v.hh:date.nw:location) +
##     (1 + iv1 + iv2 | year:v.hh)
##    Data: T
## Control:
## glmerControl(optimizer = "nloptwrap", optCtrl = list(maxfun = 1e+06))
##
##      AIC      BIC   logLik deviance df.resid
##  15703.3  16004.4  -7804.6  15609.3     4429
##
## Scaled residuals:
##      Min       1Q   Median       3Q      Max
## -0.80136 -0.26584 -0.13555  0.09464  0.99416
##
## Random effects:
##  Groups                             Name        Variance  Std.Dev.  Corr
##  village:year:v.hh:date.nw:location (Intercept) 5.162e+00 2.2719257
##  village:year:v.hh:date.nw          (Intercept) 5.711e-01 0.7556870
##  year:v.hh                          (Intercept) 5.390e-05 0.0073416
##                                     iv1         3.595e-01 0.5996026 -0.08
##                                     iv2         8.290e-02 0.2879286  0.07
##  village:year:intervalc             (Intercept) 8.077e-08 0.0002842
##  village:year                       (Intercept) 0.000e+00 0.0000000
##
```

```
##
##
##
##
##  -1.00
##
##
## Number of obs: 4476, groups:
## village:year:v.hh:date.nw:location, 4476; village:year:v.hh:date.nw, 2239; year:v.hh, 160; village:year:int
##
## Fixed effects:
##                                         Estimate Std. Error z value
## locationC                              -3.792626   0.190005 -19.961
## locationF                              -3.705031   0.189904 -19.510
## year2016:seasondry                      1.720610   0.257835   6.673
## year2017:seasondry                     -2.253668   0.279016  -8.077
## year2016:seasonwet                      3.683826   0.229427  16.057
## year2016:seasondry:treatment2control:iv1      0.247837   0.238942   1.037
## year2017:seasondry:treatment2control:iv1     -0.353328   0.258899  -1.365
## year2016:seasonwet:treatment2control:iv1      0.008344   0.152002   0.055
## year2017:seasonwet:treatment2control:iv1      0.011861   0.226709   0.052
## year2016:seasondry:treatment2cats:iv1         2.471573   1.863955   1.326
## year2017:seasondry:treatment2cats:iv1         0.032786   1.010965   0.032
## year2016:seasonwet:treatment2cats:iv1         0.585051   0.582159   1.005
## year2017:seasonwet:treatment2cats:iv1        -3.511135   0.928452  -3.782
## year2016:seasondry:treatment2rodenticide:iv1 -0.427074   1.898870  -0.225
## year2017:seasondry:treatment2rodenticide:iv1 -1.670625   1.224539  -1.364
## year2016:seasonwet:treatment2rodenticide:iv1  0.826384   0.585318   1.412
## year2017:seasonwet:treatment2rodenticide:iv1 -4.698395   1.089330  -4.313
## year2016:seasondry:treatment2traps:iv1        1.001451   1.873107   0.535
## year2017:seasondry:treatment2traps:iv1        0.241916   1.078992   0.224
## year2016:seasonwet:treatment2traps:iv1        1.267344   0.581045   2.181
## year2017:seasonwet:treatment2traps:iv1       -1.042846   0.878298  -1.187
## year2016:seasondry:treatment2control:iv2      0.082694   0.339911   0.243
## year2017:seasondry:treatment2control:iv2     -0.114780   0.229215  -0.501
## year2016:seasonwet:treatment2control:iv2     -0.381839   0.102378  -3.730
## year2017:seasonwet:treatment2control:iv2     -0.521092   0.187062  -2.786
## year2016:seasondry:treatment2cats:iv2        -3.250239   1.930252  -1.684
## year2017:seasondry:treatment2cats:iv2        -0.058644   0.581575  -0.101
## year2016:seasonwet:treatment2cats:iv2        -1.040382   0.290644  -3.580
## year2017:seasonwet:treatment2cats:iv2         1.422324   0.527201   2.698
## year2016:seasondry:treatment2rodenticide:iv2 -0.011436   1.950263  -0.006
## year2017:seasondry:treatment2rodenticide:iv2  0.608957   0.704583   0.864
## year2016:seasonwet:treatment2rodenticide:iv2 -1.313159   0.294964  -4.452
## year2017:seasonwet:treatment2rodenticide:iv2  1.670735   0.627740   2.662
## year2016:seasondry:treatment2traps:iv2       -1.275490   1.922109  -0.664
## year2017:seasondry:treatment2traps:iv2       -0.376943   0.639003  -0.590
## year2016:seasonwet:treatment2traps:iv2       -1.353573   0.289401  -4.677
## year2017:seasonwet:treatment2traps:iv2        0.080876   0.504602   0.160
##                                              Pr(>|z|)
## locationC                                     < 2e-16
## locationF                                     < 2e-16
## year2016:seasondry                           2.50e-11
## year2017:seasondry                           6.63e-16
## year2016:seasonwet                            < 2e-16
## year2016:seasondry:treatment2control:iv1     0.299631
## year2017:seasondry:treatment2control:iv1     0.172337
## year2016:seasonwet:treatment2control:iv1     0.956221
## year2017:seasonwet:treatment2control:iv1     0.958276
## year2016:seasondry:treatment2cats:iv1        0.184845
```

```
## year2017:seasondry:treatment2cats:iv1        0.974129
## year2016:seasonwet:treatment2cats:iv1        0.314912
## year2017:seasonwet:treatment2cats:iv1        0.000156
## year2016:seasondry:treatment2rodenticide:iv1 0.822050
## year2017:seasondry:treatment2rodenticide:iv1 0.172477
## year2016:seasonwet:treatment2rodenticide:iv1 0.157993
## year2017:seasonwet:treatment2rodenticide:iv1 1.61e-05
## year2016:seasondry:treatment2traps:iv1       0.592894
## year2017:seasondry:treatment2traps:iv1       0.822597
## year2016:seasonwet:treatment2traps:iv1       0.029173
## year2017:seasonwet:treatment2traps:iv1       0.235090
## year2016:seasondry:treatment2control:iv2     0.807789
## year2017:seasondry:treatment2control:iv2     0.616547
## year2016:seasonwet:treatment2control:iv2     0.000192
## year2017:seasonwet:treatment2control:iv2     0.005342
## year2016:seasondry:treatment2cats:iv2        0.092212
## year2017:seasondry:treatment2cats:iv2        0.919681
## year2016:seasonwet:treatment2cats:iv2        0.000344
## year2017:seasonwet:treatment2cats:iv2        0.006978
## year2016:seasondry:treatment2rodenticide:iv2 0.995322
## year2017:seasondry:treatment2rodenticide:iv2 0.387434
## year2016:seasonwet:treatment2rodenticide:iv2 8.51e-06
## year2017:seasonwet:treatment2rodenticide:iv2 0.007779
## year2016:seasondry:treatment2traps:iv2       0.506954
## year2017:seasondry:treatment2traps:iv2       0.555263
## year2016:seasonwet:treatment2traps:iv2       2.91e-06
## year2017:seasonwet:treatment2traps:iv2       0.872663
```

```
##
## Correlation matrix not shown by default, as p = 37 > 12.
## Use print(x, correlation=TRUE)  or
##     vcov(x)        if you need it
```

```
## fit warnings:
## fixed-effect model matrix is rank deficient so dropping 1 column / coefficient
## convergence code: 0
## boundary (singular) fit: see ?isSingular
```

```
# Residual analysis using DHARMa
simulationOutput <- simulateResiduals(fittedModel = Tglmero, n=5000, plot=TRUE)
```

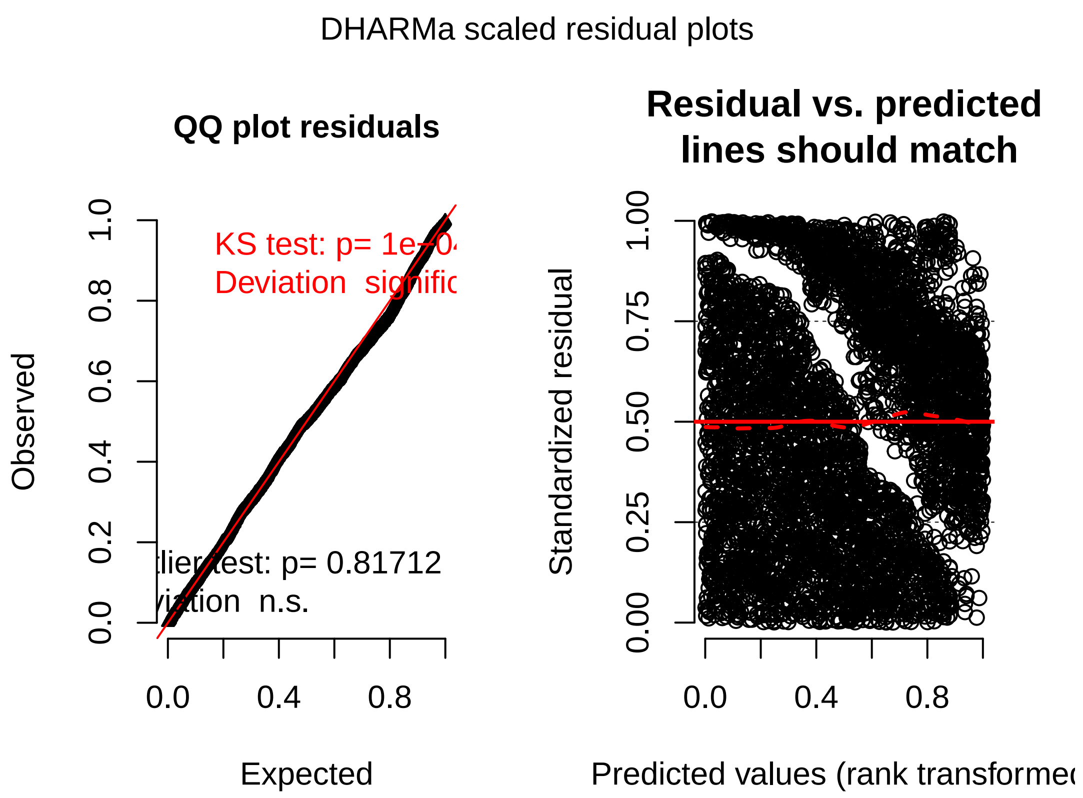


```r
# We find slight deviations in the residual patterns.
# Again, the small P-value for the KS test is not too disturbing, given the size of the dataset (n=4480)

# Compare treatment time trends between year-season combinations

Tglmero.YS <- glmer(cbind(kt,nt-kt) ~ -1 + location + treatment2:iv1 + treatment2:iv2
                      + (1 | village:year) + (1 | village:year:intervalc)
                      + (1 | village:year:v.hh:date.nw) + (1 | village:year:v.hh:date.nw:location)
                      + (1 + iv1 + iv2 | year:v.hh),
                   family=binomial(link=logit), data=T,
                    control = glmerControl(optimizer="nloptwrap", optCtrl = list(maxfun = 1000000)))
saveRDS(Tglmero.YS, "C:/MyData/OneDrive - WageningenUR/Data/Inge Krijger/final/Tiles/TglmeroYS")
Tglmero.YS <- readRDS("C:/MyData/OneDrive - WageningenUR/Data/Inge Krijger/final/Tiles/TglmeroYS")
anova(Tglmero, Tglmero.YS)
```

```
## Data: T
## Models:
## Tglmero.YS: cbind(kt, nt - kt) ~ -1 + location + treatment2:iv1 + treatment2:iv2 +
## Tglmero.YS:     (1 | village:year) + (1 | village:year:intervalc) + (1 |
## Tglmero.YS:     village:year:v.hh:date.nw) + (1 | village:year:v.hh:date.nw:location) +
## Tglmero.YS:     (1 + iv1 + iv2 | year:v.hh)
## Tglmero: cbind(kt, nt - kt) ~ -1 + location + year:season + year:season:treatment2:iv1 +
## Tglmero:     year:season:treatment2:iv2 + (1 | village:year) + (1 | village:year:intervalc) +
## Tglmero:     (1 | village:year:v.hh:date.nw) + (1 | village:year:v.hh:date.nw:location) +
## Tglmero:     (1 + iv1 + iv2 | year:v.hh)
##            Df   AIC   BIC  logLik deviance  Chisq Chi Df Pr(>Chisq)
## Tglmero.YS 20 15973 16101 -7966.3    15933
## Tglmero    47 15703 16004 -7804.6    15609 323.38     27  < 2.2e-16
```

```r
# In this test, the coefficients for intercepts are compared (3 df), coefficients for linear terms per treatme
# (so, 4x3=12 df) and for quadratic terms (4x3=12 df) are compared. In total 27 df.
# We conclude that time trends are significantly different among year-season combinations.
```

```
# Now pairwise comparisons between year-season combinations:

# Order of R$YS is: "2016.wet" "2016.dry" "2017.wet" "2017.dry"

# 2016-wet versus 2016-dry:
T$YS12 <- T$YS; levels(T$YS12) <- c(1,1,3,4)
Tglmero.12 <- glmer(cbind(kt,nt-kt) ~
                    -1 + location + YS12 + YS12:treatment2:iv1 + YS12:treatment2:iv2
                    + (1 | village:year) + (1 | village:year:intervalc) + (1 | village:year:v.hh:date.nw)
                    + (1 | village:year:v.hh:date.nw:location) + (1 + iv1 + iv2 | year:v.hh),
                    family=binomial(link=logit), data=T,
                    control = glmerControl(optimizer="nloptwrap", optCtrl = list(maxfun = 1000000)))
```

```
## boundary (singular) fit: see ?isSingular
```

```
saveRDS(Tglmero.12, "C:/MyData/OneDrive - WageningenUR/Data/Inge Krijger/final/Tiles/Tglmero12")
Tglmero.12 <- readRDS("C:/MyData/OneDrive - WageningenUR/Data/Inge Krijger/final/Tiles/Tglmero12")
anova(Tglmero, Tglmero.12)
```

```
## Data: T
## Models:
## Tglmero.12: cbind(kt, nt - kt) ~ -1 + location + YS12 + YS12:treatment2:iv1 +
## Tglmero.12:     YS12:treatment2:iv2 + (1 | village:year) + (1 | village:year:intervalc) +
## Tglmero.12:     (1 | village:year:v.hh:date.nw) + (1 | village:year:v.hh:date.nw:location) +
## Tglmero.12:     (1 + iv1 + iv2 | year:v.hh)
## Tglmero: cbind(kt, nt - kt) ~ -1 + location + year:season + year:season:treatment2:iv1 +
## Tglmero:     year:season:treatment2:iv2 + (1 | village:year) + (1 | village:year:intervalc) +
## Tglmero:     (1 | village:year:v.hh:date.nw) + (1 | village:year:v.hh:date.nw:location) +
## Tglmero:     (1 + iv1 + iv2 | year:v.hh)
##            Df   AIC   BIC  logLik deviance  Chisq Chi Df Pr(>Chisq)
## Tglmero.12 38 15842 16086 -7883.3    15766
## Tglmero    47 15703 16004 -7804.6    15609 157.21      9  < 2.2e-16
```

```
# Time trends in 2016-wet and 2016-dry are significantly different

# 2016-wet versus 2017-wet:
T$YS13 <- T$YS; levels(T$YS13) <- c(1,2,1,4)
Tglmero.13 <- glmer(cbind(kt,nt-kt) ~
                    -1 + location + YS13 + YS13:treatment2:iv1 + YS13:treatment2:iv2
                    + (1 | village:year) + (1 | village:year:intervalc) + (1 | village:year:v.hh:date.nw)
                    + (1 | village:year:v.hh:date.nw:location) + (1 + iv1 + iv2 | year:v.hh),
                    family=binomial(link=logit), data=T,
                    control = glmerControl(optimizer="nloptwrap", optCtrl = list(maxfun = 1000000)))
saveRDS(Tglmero.13, "C:/MyData/OneDrive - WageningenUR/Data/Inge Krijger/final/Tiles/Tglmero13")
Tglmero.13 <- readRDS("C:/MyData/OneDrive - WageningenUR/Data/Inge Krijger/final/Tiles/Tglmero13")
anova(Tglmero, Tglmero.13)
```

```
## Data: T
## Models:
## Tglmero.13: cbind(kt, nt - kt) ~ -1 + location + YS13 + YS13:treatment2:iv1 +
## Tglmero.13:     YS13:treatment2:iv2 + (1 | village:year) + (1 | village:year:intervalc) +
## Tglmero.13:     (1 | village:year:v.hh:date.nw) + (1 | village:year:v.hh:date.nw:location) +
## Tglmero.13:     (1 + iv1 + iv2 | year:v.hh)
## Tglmero: cbind(kt, nt - kt) ~ -1 + location + year:season + year:season:treatment2:iv1 +
## Tglmero:     year:season:treatment2:iv2 + (1 | village:year) + (1 | village:year:intervalc) +
## Tglmero:     (1 | village:year:v.hh:date.nw) + (1 | village:year:v.hh:date.nw:location) +
## Tglmero:     (1 + iv1 + iv2 | year:v.hh)
##            Df   AIC   BIC  logLik deviance  Chisq Chi Df Pr(>Chisq)
## Tglmero.13 38 15954 16197 -7938.8    15878
## Tglmero    47 15703 16004 -7804.6    15609 268.39      9  < 2.2e-16
```

```r
# Time trends in 2016-wet and 2017-wet are significantly different

# 2016-wet versus 2017-dry:
T$YS14 <- T$YS; levels(T$YS14) <- c(1,2,3,1)
Tglmero.14 <- glmer(cbind(kt,nt-kt) ~
                    -1 + location + YS14 + YS14:treatment2:iv1 + YS14:treatment2:iv2
                    + (1 | village:year) + (1 | village:year:intervalc) + (1 | village:year:v.hh:date.nw)
                    + (1 | village:year:v.hh:date.nw:location) + (1 + iv1 + iv2 | year:v.hh),
                    family=binomial(link=logit), data=T,
                    control = glmerControl(optimizer="nloptwrap", optCtrl = list(maxfun = 1000000)))
saveRDS(Tglmero.14, "C:/MyData/OneDrive - WageningenUR/Data/Inge Krijger/final/Tiles/Tglmero14")
Tglmero.14 <- readRDS("C:/MyData/OneDrive - WageningenUR/Data/Inge Krijger/final/Tiles/Tglmero14")
anova(Tglmero, Tglmero.14)
```

```
## Data: T
## Models:
## Tglmero.14: cbind(kt, nt - kt) ~ -1 + location + YS14 + YS14:treatment2:iv1 +
## Tglmero.14:     YS14:treatment2:iv2 + (1 | village:year) + (1 | village:year:intervalc) +
## Tglmero.14:     (1 | village:year:v.hh:date.nw) + (1 | village:year:v.hh:date.nw:location) +
## Tglmero.14:     (1 + iv1 + iv2 | year:v.hh)
## Tglmero: cbind(kt, nt - kt) ~ -1 + location + year:season + year:season:treatment2:iv1 +
## Tglmero:     year:season:treatment2:iv2 + (1 | village:year) + (1 | village:year:intervalc) +
## Tglmero:     (1 | village:year:v.hh:date.nw) + (1 | village:year:v.hh:date.nw:location) +
## Tglmero:     (1 + iv1 + iv2 | year:v.hh)
##            Df   AIC   BIC  logLik deviance  Chisq Chi Df Pr(>Chisq)
## Tglmero.14 38 15952 16195 -7937.9    15876
## Tglmero    47 15703 16004 -7804.6    15609 266.47      9  < 2.2e-16
```

```r
# Time trends in 2016-wet and 2017-dry are significantly different

# 2016-dry versus 2017-wet:
T$YS23 <- T$YS; levels(T$YS23) <- c(1,2,2,4)
Tglmero.23 <- glmer(cbind(kt,nt-kt) ~
                    -1 + location + YS23 + YS23:treatment2:iv1 + YS23:treatment2:iv2
                    + (1 | village:year) + (1 | village:year:intervalc) + (1 | village:year:v.hh:date.nw)
                    + (1 | village:year:v.hh:date.nw:location) + (1 + iv1 + iv2 | year:v.hh),
                    family=binomial(link=logit), data=T,
                    control = glmerControl(optimizer="nloptwrap", optCtrl = list(maxfun = 1000000)))
saveRDS(Tglmero.23, "C:/MyData/OneDrive - WageningenUR/Data/Inge Krijger/final/Tiles/Tglmero23")
Tglmero.23 <- readRDS("C:/MyData/OneDrive - WageningenUR/Data/Inge Krijger/final/Tiles/Tglmero23")
anova(Tglmero, Tglmero.23)
```

```
## Data: T
## Models:
## Tglmero.23: cbind(kt, nt - kt) ~ -1 + location + YS23 + YS23:treatment2:iv1 +
## Tglmero.23:     YS23:treatment2:iv2 + (1 | village:year) + (1 | village:year:intervalc) +
## Tglmero.23:     (1 | village:year:v.hh:date.nw) + (1 | village:year:v.hh:date.nw:location) +
## Tglmero.23:     (1 + iv1 + iv2 | year:v.hh)
## Tglmero: cbind(kt, nt - kt) ~ -1 + location + year:season + year:season:treatment2:iv1 +
## Tglmero:     year:season:treatment2:iv2 + (1 | village:year) + (1 | village:year:intervalc) +
## Tglmero:     (1 | village:year:v.hh:date.nw) + (1 | village:year:v.hh:date.nw:location) +
## Tglmero:     (1 + iv1 + iv2 | year:v.hh)
##            Df   AIC   BIC  logLik deviance  Chisq Chi Df Pr(>Chisq)
## Tglmero.23 38 15908 16152 -7916.2    15832
## Tglmero    47 15703 16004 -7804.6    15609 223.07      9  < 2.2e-16
```

```r
# Time trends in 2016-dry and 2017-wet are significantly different

# 2016-dry versus 2017-dry:
T$YS24 <- T$YS; levels(T$YS24) <- c(1,2,3,2)
Tglmero.24 <- glmer(cbind(kt,nt-kt) ~
```

```
              -1 + location + YS24 + YS24:treatment2:iv1 + YS24:treatment2:iv2
              + (1 | village:year) + (1 | village:year:intervalc) + (1 | village:year:v.hh:date.nw)
              + (1 | village:year:v.hh:date.nw:location) + (1 + iv1 + iv2 | year:v.hh),
              family=binomial(link=logit), data=T,
              control = glmerControl(optimizer="nloptwrap", optCtrl = list(maxfun = 1000000)))
saveRDS(Tglmero.24, "C:/MyData/OneDrive - WageningenUR/Data/Inge Krijger/final/Tiles/Tglmero24")
Tglmero.24 <- readRDS("C:/MyData/OneDrive - WageningenUR/Data/Inge Krijger/final/Tiles/Tglmero24")
anova(Tglmero, Tglmero.24)
```

```
## Data: T
## Models:
## Tglmero.24: cbind(kt, nt - kt) ~ -1 + location + YS24 + YS24:treatment2:iv1 +
## Tglmero.24:     YS24:treatment2:iv2 + (1 | village:year) + (1 | village:year:intervalc) +
## Tglmero.24:     (1 | village:year:v.hh:date.nw) + (1 | village:year:v.hh:date.nw:location) +
## Tglmero.24:     (1 + iv1 + iv2 | year:v.hh)
## Tglmero: cbind(kt, nt - kt) ~ -1 + location + year:season + year:season:treatment2:iv1 +
## Tglmero:     year:season:treatment2:iv2 + (1 | village:year) + (1 | village:year:intervalc) +
## Tglmero:     (1 | village:year:v.hh:date.nw) + (1 | village:year:v.hh:date.nw:location) +
## Tglmero:     (1 + iv1 + iv2 | year:v.hh)
##            Df   AIC   BIC  logLik deviance  Chisq Chi Df Pr(>Chisq)
## Tglmero.24 38 15929 16172 -7926.5    15853
## Tglmero    47 15703 16004 -7804.6    15609 243.66      9  < 2.2e-16
```

```
# Time trends in 2016-dry and 2017-dry are significantly different

# 2017-wet versus 2017-dry:
T$YS34 <- T$YS; levels(T$YS34) <- c(1,2,3,3)
Tglmero.34 <- glmer(cbind(kt,nt-kt) ~
                    -1 + location + YS34 + YS34:treatment2:iv1 + YS34:treatment2:iv2
                    + (1 | village:year) + (1 | village:year:intervalc) + (1 | village:year:v.hh:date.nw)
                    + (1 | village:year:v.hh:date.nw:location) + (1 + iv1 + iv2 | year:v.hh),
                    family=binomial(link=logit), data=T,
                    control = glmerControl(optimizer="nloptwrap", optCtrl = list(maxfun = 1000000)))
```

```
## boundary (singular) fit: see ?isSingular
```

```
saveRDS(Tglmero.34, "C:/MyData/OneDrive - WageningenUR/Data/Inge Krijger/final/Tiles/Tglmero34")
Tglmero.34 <- readRDS("C:/MyData/OneDrive - WageningenUR/Data/Inge Krijger/final/Tiles/Tglmero34")
anova(Tglmero, Tglmero.34)
```

```
## Data: T
## Models:
## Tglmero.34: cbind(kt, nt - kt) ~ -1 + location + YS34 + YS34:treatment2:iv1 +
## Tglmero.34:     YS34:treatment2:iv2 + (1 | village:year) + (1 | village:year:intervalc) +
## Tglmero.34:     (1 | village:year:v.hh:date.nw) + (1 | village:year:v.hh:date.nw:location) +
## Tglmero.34:     (1 + iv1 + iv2 | year:v.hh)
## Tglmero: cbind(kt, nt - kt) ~ -1 + location + year:season + year:season:treatment2:iv1 +
## Tglmero:     year:season:treatment2:iv2 + (1 | village:year) + (1 | village:year:intervalc) +
## Tglmero:     (1 | village:year:v.hh:date.nw) + (1 | village:year:v.hh:date.nw:location) +
## Tglmero:     (1 + iv1 + iv2 | year:v.hh)
##            Df   AIC   BIC  logLik deviance  Chisq Chi Df Pr(>Chisq)
## Tglmero.34 38 15806 16050 -7865.2    15730
## Tglmero    47 15703 16004 -7804.6    15609 121.13      9  < 2.2e-16
```

```
# Time trends in 2017-wet and 2017-dry are significantly different

# Compare time trends between treatments per year-season combination:
# Within year 2016-dry:
C1 <- rbind(c(0,0, 0,0,0,  1,0,0,0, -1,0,0,0, 0,0,0,0, 0,0,0,0,  0,0,0,0, 0,0,0,0, 0,0,0,0, 0,0,0,0),
            c(0,0, 0,0,0,  1,0,0,0, 0,0,0,0, -1,0,0,0, 0,0,0,0,  0,0,0,0, 0,0,0,0, 0,0,0,0, 0,0,0,0),
            c(0,0, 0,0,0,  1,0,0,0, 0,0,0,0, 0,0,0,0, -1,0,0,0,  0,0,0,0, 0,0,0,0, 0,0,0,0, 0,0,0,0),
```

```r
            c(0,0, 0,0,0,  0,0,0,0, 0,0,0,0, 0,0,0,0, 0,0,0,0,  1,0,0,0, -1,0,0,0, 0,0,0,0, 0,0,0,0),
            c(0,0, 0,0,0,  0,0,0,0, 0,0,0,0, 0,0,0,0, 0,0,0,0,  1,0,0,0, 0,0,0,0, -1,0,0,0, 0,0,0,0),
            c(0,0, 0,0,0,  0,0,0,0, 0,0,0,0, 0,0,0,0, 0,0,0,0,  1,0,0,0, 0,0,0,0, 0,0,0,0, -1,0,0,0))
linearHypothesis(Tglmero, C1)
```

```
## Linear hypothesis test
##
## Hypothesis:
## year2016:seasondry:treatment2control:iv1 - year2016:seasondry:treatment2cats:iv1 = 0
## year2016:seasondry:treatment2control:iv1 - year2016:seasondry:treatment2rodenticide:iv1 = 0
## year2016:seasondry:treatment2control:iv1 - year2016:seasondry:treatment2traps:iv1 = 0
## year2016:seasondry:treatment2control:iv2 - year2016:seasondry:treatment2cats:iv2 = 0
## year2016:seasondry:treatment2control:iv2 - year2016:seasondry:treatment2rodenticide:iv2 = 0
## year2016:seasondry:treatment2control:iv2 - year2016:seasondry:treatment2traps:iv2 = 0
##
## Model 1: restricted model
## Model 2: cbind(kt, nt - kt) ~ -1 + location + year:season + year:season:treatment2:iv1 +
##     year:season:treatment2:iv2 + (1 | village:year) + (1 | village:year:intervalc) +
##     (1 | village:year:v.hh:date.nw) + (1 | village:year:v.hh:date.nw:location) +
##     (1 + iv1 + iv2 | year:v.hh)
##
##   Df  Chisq Pr(>Chisq)
## 1
## 2  6 5.5261     0.4783
```

```r
# No time trend differences between treatments in 2016-dry

# Within year 2017-dry:
C2 <- rbind(c(0,0, 0,0,0,  0,1,0,0, 0,-1,0,0, 0,0,0,0, 0,0,0,0,  0,0,0,0, 0,0,0,0, 0,0,0,0, 0,0,0,0),
            c(0,0, 0,0,0,  0,1,0,0, 0,0,0,0, 0,-1,0,0, 0,0,0,0,  0,0,0,0, 0,0,0,0, 0,0,0,0, 0,0,0,0),
            c(0,0, 0,0,0,  0,1,0,0, 0,0,0,0, 0,0,0,0, 0,-1,0,0,  0,0,0,0, 0,0,0,0, 0,0,0,0, 0,0,0,0),

            c(0,0, 0,0,0,  0,0,0,0, 0,0,0,0, 0,0,0,0, 0,0,0,0,  0,1,0,0, 0,-1,0,0, 0,0,0,0, 0,0,0,0),
            c(0,0, 0,0,0,  0,0,0,0, 0,0,0,0, 0,0,0,0, 0,0,0,0,  0,1,0,0, 0,0,0,0, 0,-1,0,0, 0,0,0,0),
            c(0,0, 0,0,0,  0,0,0,0, 0,0,0,0, 0,0,0,0, 0,0,0,0,  0,1,0,0, 0,0,0,0, 0,0,0,0, 0,-1,0,0))
linearHypothesis(Tglmero, C2)
```

```
## Linear hypothesis test
##
## Hypothesis:
## year2017:seasondry:treatment2control:iv1 - year2017:seasondry:treatment2cats:iv1 = 0
## year2017:seasondry:treatment2control:iv1 - year2017:seasondry:treatment2rodenticide:iv1 = 0
## year2017:seasondry:treatment2control:iv1 - year2017:seasondry:treatment2traps:iv1 = 0
## year2017:seasondry:treatment2control:iv2 - year2017:seasondry:treatment2cats:iv2 = 0
## year2017:seasondry:treatment2control:iv2 - year2017:seasondry:treatment2rodenticide:iv2 = 0
## year2017:seasondry:treatment2control:iv2 - year2017:seasondry:treatment2traps:iv2 = 0
##
## Model 1: restricted model
## Model 2: cbind(kt, nt - kt) ~ -1 + location + year:season + year:season:treatment2:iv1 +
##     year:season:treatment2:iv2 + (1 | village:year) + (1 | village:year:intervalc) +
##     (1 | village:year:v.hh:date.nw) + (1 | village:year:v.hh:date.nw:location) +
##     (1 + iv1 + iv2 | year:v.hh)
##
##   Df  Chisq Pr(>Chisq)
## 1
## 2  6 4.6395     0.5908
```

```r
# No time trend differences between treatments in 2017-dry

# Within year 2016-wet:
C3 <- rbind(c(0,0, 0,0,0,  0,0,1,0, 0,0,-1,0, 0,0,0,0, 0,0,0,0,  0,0,0,0, 0,0,0,0, 0,0,0,0, 0,0,0,0),
```

```r
           c(0,0, 0,0,0,  0,0,1,0, 0,0,0,0, 0,0,-1,0, 0,0,0,0,  0,0,0,0, 0,0,0,0, 0,0,0,0, 0,0,0,0),
           c(0,0, 0,0,0,  0,0,1,0, 0,0,0,0, 0,0,0,0, 0,0,-1,0,  0,0,0,0, 0,0,0,0, 0,0,0,0, 0,0,0,0),

           c(0,0, 0,0,0,  0,0,0,0, 0,0,0,0, 0,0,0,0, 0,0,0,0,  0,0,1,0, 0,0,-1,0, 0,0,0,0, 0,0,0,0),
           c(0,0, 0,0,0,  0,0,0,0, 0,0,0,0, 0,0,0,0, 0,0,0,0,  0,0,1,0, 0,0,0,0, 0,0,-1,0, 0,0,0,0),
           c(0,0, 0,0,0,  0,0,0,0, 0,0,0,0, 0,0,0,0, 0,0,0,0,  0,0,1,0, 0,0,0,0, 0,0,0,0, 0,0,-1,0))
linearHypothesis(Tglmero, C3)
```

```
## Linear hypothesis test
##
## Hypothesis:
## year2016:seasonwet:treatment2control:iv1 - year2016:seasonwet:treatment2cats:iv1 = 0
## year2016:seasonwet:treatment2control:iv1 - year2016:seasonwet:treatment2rodenticide:iv1 = 0
## year2016:seasonwet:treatment2control:iv1 - year2016:seasonwet:treatment2traps:iv1 = 0
## year2016:seasonwet:treatment2control:iv2 - year2016:seasonwet:treatment2cats:iv2 = 0
## year2016:seasonwet:treatment2control:iv2 - year2016:seasonwet:treatment2rodenticide:iv2 = 0
## year2016:seasonwet:treatment2control:iv2 - year2016:seasonwet:treatment2traps:iv2 = 0
##
## Model 1: restricted model
## Model 2: cbind(kt, nt - kt) ~ -1 + location + year:season + year:season:treatment2:iv1 +
##     year:season:treatment2:iv2 + (1 | village:year) + (1 | village:year:intervalc) +
##     (1 | village:year:v.hh:date.nw) + (1 | village:year:v.hh:date.nw:location) +
##     (1 + iv1 + iv2 | year:v.hh)
##
##   Df  Chisq Pr(>Chisq)
## 1
## 2  6 39.515  5.673e-07
```

```r
# Time trend differences between treatments in 2016-wet

# Within year 2017-wet:
C4 <- rbind(c(0,0, 0,0,0,  0,0,0,1, 0,0,0,-1, 0,0,0,0, 0,0,0,0,  0,0,0,0, 0,0,0,0, 0,0,0,0, 0,0,0,0),
            c(0,0, 0,0,0,  0,0,0,1, 0,0,0,0, 0,0,0,-1, 0,0,0,0,  0,0,0,0, 0,0,0,0, 0,0,0,0, 0,0,0,0),
            c(0,0, 0,0,0,  0,0,0,1, 0,0,0,0, 0,0,0,0, 0,0,0,-1,  0,0,0,0, 0,0,0,0, 0,0,0,0, 0,0,0,0),

            c(0,0, 0,0,0,  0,0,0,0, 0,0,0,0, 0,0,0,0, 0,0,0,0,  0,0,0,1, 0,0,0,-1, 0,0,0,0, 0,0,0,0),
            c(0,0, 0,0,0,  0,0,0,0, 0,0,0,0, 0,0,0,0, 0,0,0,0,  0,0,0,1, 0,0,0,0, 0,0,0,-1, 0,0,0,0),
            c(0,0, 0,0,0,  0,0,0,0, 0,0,0,0, 0,0,0,0, 0,0,0,0,  0,0,0,1, 0,0,0,0, 0,0,0,0, 0,0,0,-1))
linearHypothesis(Tglmero, C4)
```

```
## Linear hypothesis test
##
## Hypothesis:
## year2017:seasonwet:treatment2control:iv1 - year2017:seasonwet:treatment2cats:iv1 = 0
## year2017:seasonwet:treatment2control:iv1 - year2017:seasonwet:treatment2rodenticide:iv1 = 0
## year2017:seasonwet:treatment2control:iv1 - year2017:seasonwet:treatment2traps:iv1 = 0
## year2017:seasonwet:treatment2control:iv2 - year2017:seasonwet:treatment2cats:iv2 = 0
## year2017:seasonwet:treatment2control:iv2 - year2017:seasonwet:treatment2rodenticide:iv2 = 0
## year2017:seasonwet:treatment2control:iv2 - year2017:seasonwet:treatment2traps:iv2 = 0
##
## Model 1: restricted model
## Model 2: cbind(kt, nt - kt) ~ -1 + location + year:season + year:season:treatment2:iv1 +
##     year:season:treatment2:iv2 + (1 | village:year) + (1 | village:year:intervalc) +
##     (1 | village:year:v.hh:date.nw) + (1 | village:year:v.hh:date.nw:location) +
##     (1 + iv1 + iv2 | year:v.hh)
##
##   Df  Chisq Pr(>Chisq)
## 1
## 2  6 32.977   1.06e-05
```

```
# Time trend differences between treatments in 2017-wet
```

### Data on tracking tiles: Testing pairwise treatment differences at different time points within 2016-wet and within 2017-wet

```
# 2016-wet
T1.emm <- emmeans(Tglmero,"treatment2", type="response",
          at=list(year="2016", season="wet", iv1=14/sd.iv, iv2=(14/sd.iv)^2, iv3=(14/sd.iv)^3))
```

```
## NOTE: Results may be misleading due to involvement in interactions
```

```
pairs(T1.emm)
```

```
##  contrast               odds.ratio    SE  df z.ratio p.value
##  control / cats              0.882 0.195 Inf -0.568  0.9417
##  control / rodenticide       0.836 0.186 Inf -0.804  0.8524
##  control / traps             0.685 0.151 Inf -1.711  0.3181
##  cats / rodenticide          0.948 0.258 Inf -0.195  0.9974
##  cats / traps                0.777 0.211 Inf -0.929  0.7896
##  rodenticide / traps         0.819 0.222 Inf -0.735  0.8831
##
## Results are averaged over the levels of: location
## P value adjustment: tukey method for comparing a family of 4 estimates
## Tests are performed on the log odds ratio scale
```

```
CLD(T1.emm, Letters=letters)
```

```
##  treatment2   prob     SE  df asymp.LCL asymp.UCL .group
##  control     0.464 0.0397 Inf     0.387     0.542  a
##  cats        0.495 0.0498 Inf     0.399     0.591  a
##  rodenticide 0.508 0.0498 Inf     0.411     0.604  a
##  traps       0.558 0.0490 Inf     0.461     0.650  a
##
## Results are averaged over the levels of: location
## Confidence level used: 0.95
## Intervals are back-transformed from the logit scale
## P value adjustment: tukey method for comparing a family of 4 estimates
## Tests are performed on the log odds ratio scale
## significance level used: alpha = 0.05
```

```
T2.emm <- emmeans(Tglmero,"treatment2", type="response",
                  at=list(year="2016", season="wet", iv1=28/sd.iv, iv2=(28/sd.iv)^2, iv3=(28/sd.iv)^3))
```

```
## NOTE: Results may be misleading due to involvement in interactions
```

```
pairs(T2.emm)
```

```
##  contrast               odds.ratio    SE  df z.ratio p.value
##  control / cats              1.044 0.330 Inf  0.136  0.9991
##  control / rodenticide       1.060 0.336 Inf  0.184  0.9978
##  control / traps             0.725 0.229 Inf -1.019  0.7384
##  cats / rodenticide          1.016 0.389 Inf  0.040  1.0000
##  cats / traps                0.694 0.266 Inf -0.954  0.7754
##  rodenticide / traps         0.684 0.260 Inf -1.000  0.7494
##
## Results are averaged over the levels of: location
## P value adjustment: tukey method for comparing a family of 4 estimates
## Tests are performed on the log odds ratio scale
```

```
CLD(T2.emm, Letters=letters)
```

```
##  treatment2   prob     SE  df asymp.LCL asymp.UCL .group
##  rodenticide 0.388 0.0646 Inf     0.271     0.519  a
```

```
##  cats        0.392 0.0650 Inf     0.274     0.523  a
##  control     0.402 0.0404 Inf     0.326     0.483  a
##  traps       0.481 0.0678 Inf     0.353     0.612  a
##
## Results are averaged over the levels of: location
## Confidence level used: 0.95
## Intervals are back-transformed from the logit scale
## P value adjustment: tukey method for comparing a family of 4 estimates
## Tests are performed on the log odds ratio scale
## significance level used: alpha = 0.05
```

```
T3.emm <- emmeans(Tglmero,"treatment2", type="response",
                  at=list(year="2016", season="wet", iv1=42/sd.iv, iv2=(42/sd.iv)^2, iv3=(42/sd.iv)^3))
```

```
## NOTE: Results may be misleading due to involvement in interactions
```

```
pairs(T3.emm)
```

```
##  contrast              odds.ratio    SE  df z.ratio p.value
##  control / cats             1.657 0.522 Inf   1.602  0.3774
##  control / rodenticide      2.035 0.648 Inf   2.231  0.1148
##  control / traps            1.182 0.375 Inf   0.526  0.9529
##  cats / rodenticide         1.228 0.447 Inf   0.565  0.9423
##  cats / traps               0.713 0.259 Inf  -0.931  0.7881
##  rodenticide / traps        0.581 0.209 Inf  -1.514  0.4293
##
## Results are averaged over the levels of: location
## P value adjustment: tukey method for comparing a family of 4 estimates
## Tests are performed on the log odds ratio scale
```

```
CLD(T3.emm, Letters=letters)
```

```
##  treatment2   prob     SE  df asymp.LCL asymp.UCL .group
##  rodenticide 0.178 0.0380 Inf     0.115     0.265  a
##  cats        0.210 0.0431 Inf     0.138     0.307  a
##  traps       0.272 0.0511 Inf     0.183     0.382  a
##  control     0.306 0.0376 Inf     0.237     0.384  a
##
## Results are averaged over the levels of: location
## Confidence level used: 0.95
## Intervals are back-transformed from the logit scale
## P value adjustment: tukey method for comparing a family of 4 estimates
## Tests are performed on the log odds ratio scale
## significance level used: alpha = 0.05
```

```
T4.emm <- emmeans(Tglmero,"treatment2", type="response",
                  at=list(year="2016", season="wet", iv1=56/sd.iv, iv2=(56/sd.iv)^2, iv3=(56/sd.iv)^3))
```

```
## NOTE: Results may be misleading due to involvement in interactions
```

```
pairs(T4.emm)
```

```
##  contrast              odds.ratio    SE  df z.ratio p.value
##  control / cats             3.528 1.194 Inf   3.726  0.0011
##  control / rodenticide      5.920 2.080 Inf   5.060  <.0001
##  control / traps            2.973 1.020 Inf   3.175  0.0082
##  cats / rodenticide         1.678 0.615 Inf   1.413  0.4911
##  cats / traps               0.843 0.302 Inf  -0.477  0.9641
##  rodenticide / traps        0.502 0.179 Inf  -1.931  0.2150
##
## Results are averaged over the levels of: location
## P value adjustment: tukey method for comparing a family of 4 estimates
## Tests are performed on the log odds ratio scale
```

```
CLD(T4.emm, Letters=letters)
```

```
##  treatment2    prob     SE  df asymp.LCL asymp.UCL .group
##  rodenticide 0.0395 0.0100 Inf    0.0240    0.0645  a
##  cats        0.0646 0.0155 Inf    0.0401    0.1024  a
##  traps       0.0758 0.0176 Inf    0.0477    0.1183  a
##  control     0.1959 0.0361 Inf    0.1345    0.2764   b
##
## Results are averaged over the levels of: location
## Confidence level used: 0.95
## Intervals are back-transformed from the logit scale
## P value adjustment: tukey method for comparing a family of 4 estimates
## Tests are performed on the log odds ratio scale
## significance level used: alpha = 0.05
```

```
T5.emm <- emmeans(Tglmero,"treatment2", type="response",
                  at=list(year="2016", season="wet", iv1=67/sd.iv, iv2=(67/sd.iv)^2, iv3=(67/sd.iv)^3))
```

```
## NOTE: Results may be misleading due to involvement in interactions
```

```
pairs(T5.emm)
```

```
##  contrast              odds.ratio    SE  df z.ratio p.value
##  control / cats             7.851 3.965 Inf   4.081  0.0003
##  control / rodenticide     18.333 9.679 Inf   5.510  <.0001
##  control / traps            8.319 4.239 Inf   4.157  0.0002
##  cats / rodenticide         2.335 1.341 Inf   1.476  0.4519
##  cats / traps               1.060 0.592 Inf   0.103  0.9996
##  rodenticide / traps        0.454 0.255 Inf  -1.406  0.4957
##
## Results are averaged over the levels of: location
## P value adjustment: tukey method for comparing a family of 4 estimates
## Tests are performed on the log odds ratio scale
```

```
CLD(T5.emm, Letters=letters)
```

```
##  treatment2     prob      SE  df asymp.LCL asymp.UCL .group
##  rodenticide 0.00735 0.00304 Inf   0.00326    0.0165  a
##  traps       0.01606 0.00621 Inf   0.00750    0.0340  a
##  cats        0.01700 0.00667 Inf   0.00784    0.0364  a
##  control     0.11954 0.03341 Inf   0.06793    0.2019   b
##
## Results are averaged over the levels of: location
## Confidence level used: 0.95
## Intervals are back-transformed from the logit scale
## P value adjustment: tukey method for comparing a family of 4 estimates
## Tests are performed on the log odds ratio scale
## significance level used: alpha = 0.05
```

```
# 2017 wet

T1.emm <- emmeans(Tglmero,"treatment2", type="response",
                  at=list(year="2017", season="wet", iv1=14/sd.iv, iv2=(14/sd.iv)^2, iv3=(14/sd.iv)^3))
```

```
## NOTE: Results may be misleading due to involvement in interactions
```

```
pairs(T1.emm)
```

```
##  contrast              odds.ratio    SE  df z.ratio p.value
##  control / cats             3.422 1.168 Inf   3.604  0.0018
##  control / rodenticide      5.673 2.238 Inf   4.399  0.0001
##  control / traps            1.439 0.466 Inf   1.122  0.6757
##  cats / rodenticide         1.658 0.766 Inf   1.093  0.6936
##  cats / traps               0.420 0.171 Inf  -2.127  0.1444
```

```
##  rodenticide / traps        0.254 0.114 Inf -3.048  0.0123
##
## Results are averaged over the levels of: location
## P value adjustment: tukey method for comparing a family of 4 estimates
## Tests are performed on the log odds ratio scale
```

```
CLD(T1.emm, Letters=letters)
```

```
##  treatment2     prob      SE  df asymp.LCL asymp.UCL .group
##  rodenticide 0.00370 0.00133 Inf   0.00183   0.00749  a
##  cats        0.00612 0.00188 Inf   0.00335   0.01115  ab
##  traps       0.01444 0.00408 Inf   0.00828   0.02506   bc
##  control     0.02064 0.00445 Inf   0.01351   0.03142    c
##
## Results are averaged over the levels of: location
## Confidence level used: 0.95
## Intervals are back-transformed from the logit scale
## P value adjustment: tukey method for comparing a family of 4 estimates
## Tests are performed on the log odds ratio scale
## significance level used: alpha = 0.05
```

```
T2.emm <- emmeans(Tglmero,"treatment2", type="response",
                  at=list(year="2017", season="wet", iv1=28/sd.iv, iv2=(28/sd.iv)^2, iv3=(28/sd.iv)^3))
```

```
## NOTE: Results may be misleading due to involvement in interactions
```

```
pairs(T2.emm)
```

```
##  contrast              odds.ratio     SE  df z.ratio p.value
##  control / cats             4.922 2.2442 Inf   3.496  0.0027
##  control / rodenticide     12.105 6.3559 Inf   4.749  <.0001
##  control / traps            1.583 0.6832 Inf   1.064  0.7117
##  cats / rodenticide         2.459 1.4854 Inf   1.490  0.4437
##  cats / traps               0.322 0.1702 Inf  -2.143  0.1395
##  rodenticide / traps        0.131 0.0767 Inf  -3.467  0.0030
##
## Results are averaged over the levels of: location
## P value adjustment: tukey method for comparing a family of 4 estimates
## Tests are performed on the log odds ratio scale
```

```
CLD(T2.emm, Letters=letters)
```

```
##  treatment2     prob      SE  df asymp.LCL asymp.UCL .group
##  rodenticide 0.00123 0.00058 Inf  0.000491   0.00310  a
##  cats        0.00303 0.00120 Inf  0.001396   0.00656  ab
##  traps       0.00936 0.00339 Inf  0.004596   0.01897   bc
##  control     0.01474 0.00339 Inf  0.009379   0.02308    c
##
## Results are averaged over the levels of: location
## Confidence level used: 0.95
## Intervals are back-transformed from the logit scale
## P value adjustment: tukey method for comparing a family of 4 estimates
## Tests are performed on the log odds ratio scale
## significance level used: alpha = 0.05
```

```
T3.emm <- emmeans(Tglmero,"treatment2", type="response",
                  at=list(year="2017", season="wet", iv1=42/sd.iv, iv2=(42/sd.iv)^2, iv3=(42/sd.iv)^3))
```

```
## NOTE: Results may be misleading due to involvement in interactions
```

```
pairs(T3.emm)
```

```
##  contrast              odds.ratio     SE  df z.ratio p.value
##  control / cats             2.975 1.3072 Inf   2.481  0.0628
```

```
##  control / rodenticide      9.716 4.8878 Inf  4.520  <.0001
##  control / traps            1.331 0.5634 Inf  0.675  0.9064
##  cats / rodenticide         3.266 1.7391 Inf  2.222  0.1171
##  cats / traps               0.447 0.2057 Inf -1.749  0.2982
##  rodenticide / traps        0.137 0.0713 Inf -3.820  0.0008
##
## Results are averaged over the levels of: location
## P value adjustment: tukey method for comparing a family of 4 estimates
## Tests are performed on the log odds ratio scale
```

```
CLD(T3.emm, Letters=letters)
```

```
##  treatment2      prob       SE  df asymp.LCL asymp.UCL .group
##  rodenticide 0.000865 0.000365 Inf  0.000378   0.00198  a
##  cats        0.002820 0.000971 Inf  0.001435   0.00553  ab
##  traps       0.006280 0.002015 Inf  0.003345   0.01176   b
##  control     0.008342 0.002333 Inf  0.004817   0.01441   b
##
## Results are averaged over the levels of: location
## Confidence level used: 0.95
## Intervals are back-transformed from the logit scale
## P value adjustment: tukey method for comparing a family of 4 estimates
## Tests are performed on the log odds ratio scale
## significance level used: alpha = 0.05
```

```
T4.emm <- emmeans(Tglmero,"treatment2", type="response",
                at=list(year="2017", season="wet", iv1=56/sd.iv, iv2=(56/sd.iv)^2, iv3=(56/sd.iv)^3))
```

```
## NOTE: Results may be misleading due to involvement in interactions
```

```
pairs(T4.emm)
```

```
##  contrast              odds.ratio    SE  df z.ratio p.value
##  control / cats             0.756 0.478 Inf -0.443  0.9710
##  control / rodenticide      2.933 2.141 Inf  1.474  0.4531
##  control / traps            0.856 0.542 Inf -0.246  0.9948
##  cats / rodenticide         3.882 2.884 Inf  1.826  0.2612
##  cats / traps               1.132 0.734 Inf  0.192  0.9975
##  rodenticide / traps        0.292 0.217 Inf -1.657  0.3467
##
## Results are averaged over the levels of: location
## P value adjustment: tukey method for comparing a family of 4 estimates
## Tests are performed on the log odds ratio scale
```

```
CLD(T4.emm, Letters=letters)
```

```
##  treatment2     prob       SE  df asymp.LCL asymp.UCL .group
##  rodenticide 0.00128 0.000751 Inf  0.000403   0.00404  a
##  control     0.00374 0.001633 Inf  0.001584   0.00878  a
##  traps       0.00436 0.002012 Inf  0.001764   0.01075  a
##  cats        0.00494 0.002275 Inf  0.001999   0.01215  a
##
## Results are averaged over the levels of: location
## Confidence level used: 0.95
## Intervals are back-transformed from the logit scale
## P value adjustment: tukey method for comparing a family of 4 estimates
## Tests are performed on the log odds ratio scale
## significance level used: alpha = 0.05
```

### Data on tracking tiles: 2016-wet season and 2017-wet season: plots of data and fitted model

```
Ta2016W <- Ta[Ta$year==2016 & Ta$season=="wet",]
```

```r
# Results from model fitting R: cubic model; quadratic random coefficients

coefs <- fixef(Tglmero)

ic <- 0.5*(coefs[1] + coefs[2]) + coefs[5]
b0 <- rep(ic,4)

b1.co <- coefs[6:9]
b2.co <- coefs[22:25]

b1.ca <- coefs[10:13]
b2.ca <- coefs[26:29]

b1.ro <- coefs[14:17]
b2.ro <- coefs[30:33]

b1.tr <- coefs[18:21]
b2.tr <- coefs[34:37]


iv <- seq(-34,69,1)/sd.iv
iv2 <- seq(0,69,1)/sd.iv

eta.co <- b0[3] + b1.co[3]*iv  + b2.co[3]*iv^2
eta.ca <- b0[3] + b1.ca[3]*iv2 + b2.ca[3]*iv2^2
eta.ro <- b0[3] + b1.ro[3]*iv2 + b2.ro[3]*iv2^2
eta.tr <- b0[3] + b1.tr[3]*iv2 + b2.tr[3]*iv2^2

yhat.co <- 1/(1+exp(-eta.co))
yhat.ca <- 1/(1+exp(-eta.ca))
yhat.ro <- 1/(1+exp(-eta.ro))
yhat.tr <- 1/(1+exp(-eta.tr))

V <- vcov(Tglmero)

C.co <- matrix(rep(0,length(iv)*37), ncol=37)
C.co[,c(1,2,5, 8, 24)] <- cbind(0.5,0.5,1,iv,iv^2)

var.eta.co <- diag(C.co %*% V %*% t(C.co))
se.eta.co <- sqrt(var.eta.co)
lwr.co <- 1/(1+exp(-(eta.co-1.96*se.eta.co)))
upr.co <- 1/(1+exp(-(eta.co+1.96*se.eta.co)))

df.ctrl <- data.frame(iv=iv*sd.iv, yhat.co=yhat.co, lwr.co=lwr.co, upr.co=upr.co)

C.ca <- matrix(rep(0,length(iv2)*37), ncol=37)
C.ca[,c(1,2,5,12,28)] <- cbind(0.5,0.5,1,iv2,iv2^2)
var.eta.ca <- diag(C.ca %*% V %*% t(C.ca))
se.eta.ca <- sqrt(var.eta.ca)
lwr.ca <- 1/(1+exp(-(eta.ca-1.96*se.eta.ca)))
upr.ca <- 1/(1+exp(-(eta.ca+1.96*se.eta.ca)))

C.ro <- matrix(rep(0,length(iv2)*37), ncol=37)
C.ro[,c(1,2,5,16,32)] <- cbind(0.5,0.5,1,iv2,iv2^2)
var.eta.ro <- diag(C.ro %*% V %*% t(C.ro))
se.eta.ro <- sqrt(var.eta.ro)
lwr.ro <- 1/(1+exp(-(eta.ro-1.96*se.eta.ro)))
upr.ro <- 1/(1+exp(-(eta.ro+1.96*se.eta.ro)))
```

```r
C.tr <- matrix(rep(0,length(iv2)*37), ncol=37)
C.tr[,c(1,2,5,20,36)] <- cbind(0.5,0.5,1,iv2,iv2^2)
var.eta.tr <- diag(C.tr %*% V %*% t(C.tr))
se.eta.tr <- sqrt(var.eta.tr)
lwr.tr <- 1/(1+exp(-(eta.tr-1.96*se.eta.tr)))
upr.tr <- 1/(1+exp(-(eta.tr+1.96*se.eta.tr)))

df.other <- data.frame(iv2=iv2*sd.iv, yhat.ca=yhat.ca, lwr=lwr.ca, upr=upr.ca,
                       yhat.ro=yhat.ro, lwr=lwr.ro, upr=upr.ro,
                       yhat.tr=yhat.tr, lwr=lwr.tr, upr=upr.tr)

man.col <- c("#F8766D", "#7CAE00", "#C77CFF", "#00BFC4")

df.ctrl <- data.frame(iv=iv*sd.iv, yhat.co=yhat.co)
df.other <- data.frame(iv2=iv2*sd.iv, yhat.ca=yhat.ca, yhat.ro=yhat.ro, yhat.tr=yhat.tr)

man.col <- c("#F8766D", "#7CAE00", "#C77CFF", "#00BFC4")

# plot 95% confidence bands and points instead of lines
jitter <- position_jitter(width = 0.25, height = 0.01)
p <- ggplot(data=Ta2016W, aes(x=intervaldays, y=f)) +
  labs(x = "days since start treatment", y = "fraction of cells of tiles tripped upon") +
  theme_bw()+ theme(panel.grid.minor = element_blank()) +
  geom_point(aes(colour=treatment2, group=v.hh), position=jitter, size=0.75, alpha=0.75) +
  guides(colour = guide_legend(override.aes = list(alpha = 1), title="treatment")) +
  scale_colour_manual(values=man.col) +
  scale_x_continuous(breaks=c(-28,-14,0,14,28,42,56,70), labels=c("-28","-14","0","14","28","42","56","70")) +

  geom_ribbon(data=df.ctrl, mapping=aes(x=iv, y=yhat.co, ymin = lwr.co, ymax = upr.co, group=1),
              fill=man.col[1], alpha = .2) +
  geom_line(data=df.ctrl,  mapping=aes(x=iv, y=yhat.co, group=1),  size=1.1, colour=man.col[1]) +

  geom_ribbon(data=df.other, mapping=aes(x=iv2, y=yhat.ca, ymin = lwr.ca, ymax = upr.ca, group=1),
              fill=man.col[2], alpha = .2) +
  geom_line(data=df.other, mapping=aes(x=iv2, y=yhat.ca, group=1), size=1.1, colour=man.col[2]) +

  geom_ribbon(data=df.other, mapping=aes(x=iv2, y=yhat.ro, ymin = lwr.ro, ymax = upr.ro, group=1),
              fill=man.col[3], alpha = .2) +
  geom_line(data=df.other, mapping=aes(x=iv2, y=yhat.ro, group=1), size=1.1, colour=man.col[3]) +

  geom_ribbon(data=df.other, mapping=aes(x=iv2, y=yhat.tr, ymin = lwr.tr, ymax = upr.tr, group=1),
              fill=man.col[4], alpha = .2) +
  geom_line(data=df.other, mapping=aes(x=iv2, y=yhat.tr, group=1), size=1.1, colour=man.col[4])
p
```

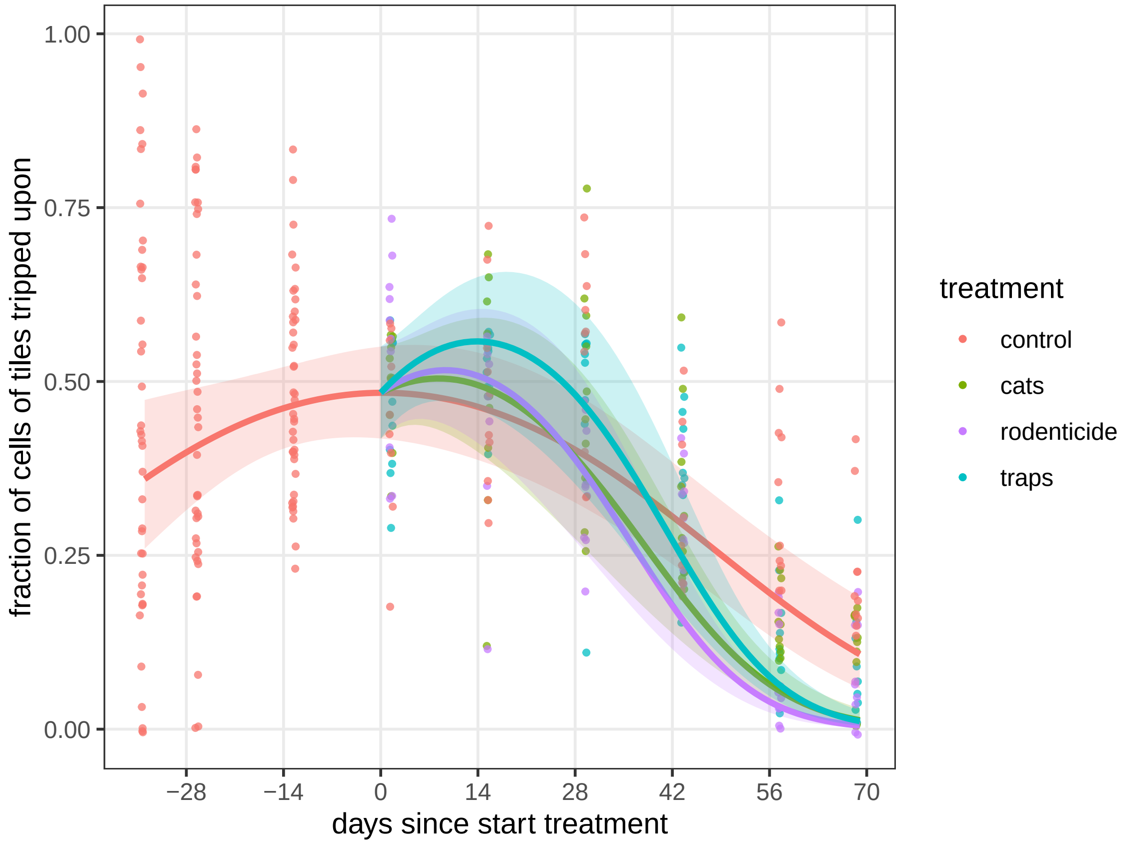


```
p <- ggplot(data=Ta2016W, aes(x=intervaldays, y=f)) +
  labs(x = "days since start treatment", y = "fraction of cells of tiles tripped upon") +
  theme_bw()+ theme(panel.grid.minor = element_blank()) +
  geom_point(aes(colour=treatment2, group=v.hh), size=1, position=jitter, size=0.75, alpha=0.75) +
  guides(colour = guide_legend(override.aes = list(alpha = 1), title="treatment")) +
  scale_colour_manual(values=man.col) +
  scale_x_continuous(breaks=c(-28,-14,0,14,28,42,56,70), labels=c("-28","-14","0","14","28","42","56","70")) +

  geom_ribbon(data=df.ctrl, mapping=aes(x=iv, y=yhat.co, ymin = lwr.co, ymax = upr.co, group=1),
              fill=man.col[1], alpha = .2, colour=man.col[1], size=0.2) +
  geom_line(data=df.ctrl,  mapping=aes(x=iv, y=yhat.co, group=1),  size=1.1, colour=man.col[1]) +

  geom_ribbon(data=df.other, mapping=aes(x=iv2, y=yhat.ca, ymin = lwr.ca, ymax = upr.ca, group=1),
              fill=man.col[2], alpha = .2, colour=man.col[2], size=0.2) +
  geom_line(data=df.other, mapping=aes(x=iv2, y=yhat.ca, group=1), size=1.1, colour=man.col[2]) +

  geom_ribbon(data=df.other, mapping=aes(x=iv2, y=yhat.ro, ymin = lwr.ro, ymax = upr.ro, group=1),
              fill=man.col[3], alpha = .2, colour=man.col[3], size=0.2) +
  geom_line(data=df.other, mapping=aes(x=iv2, y=yhat.ro, group=1), size=1.1, colour=man.col[3]) +

  geom_ribbon(data=df.other, mapping=aes(x=iv2, y=yhat.tr, ymin = lwr.tr, ymax = upr.tr, group=1),
              fill=man.col[4], alpha = .2, colour=man.col[4], size=0.2) +
  geom_line(data=df.other, mapping=aes(x=iv2, y=yhat.tr, group=1), size=1.1, colour=man.col[4])
p
```

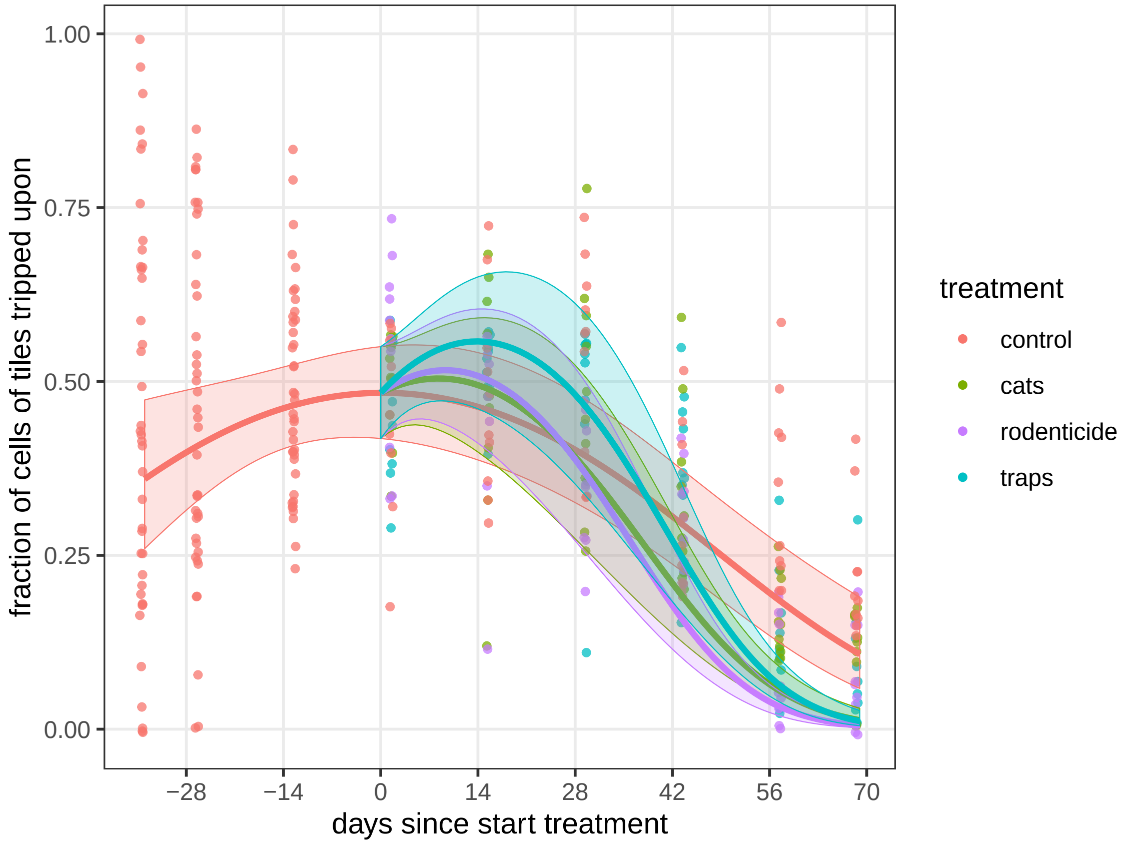


```
# 2017 wet also shows significant differences among treatments

Ta2017W <- Ta[Ta$year==2017 & Ta$season=="wet",]

ic <- 0.5*(coefs[1] + coefs[2]) # ic is different
b0 <- rep(ic,4)

b1.co <- coefs[6:9]
b2.co <- coefs[22:25]

b1.ca <- coefs[10:13]
b2.ca <- coefs[26:29]

b1.ro <- coefs[14:17]
b2.ro <- coefs[30:33]

b1.tr <- coefs[18:21]
b2.tr <- coefs[34:37]

iv <- seq(-26.5,57.5,1)/sd.iv
iv2 <- seq(0,57.5,1)/sd.iv

eta.co <- b0[4] + b1.co[4]*iv  + b2.co[4]*iv^2
eta.ca <- b0[4] + b1.ca[4]*iv2 + b2.ca[4]*iv2^2
eta.ro <- b0[4] + b1.ro[4]*iv2 + b2.ro[4]*iv2^2
eta.tr <- b0[4] + b1.tr[4]*iv2 + b2.tr[4]*iv2^2

yhat.co <- 1/(1+exp(-eta.co))
yhat.ca <- 1/(1+exp(-eta.ca))
yhat.ro <- 1/(1+exp(-eta.ro))
yhat.tr <- 1/(1+exp(-eta.tr))
```

```r
V <- vcov(Tglmero)

C.co <- matrix(rep(0,length(iv)*37), ncol=37)
C.co[,c(1,2, 9, 25)] <- cbind(0.5,0.5, iv,iv^2)

var.eta.co <- diag(C.co %*% V %*% t(C.co))
se.eta.co <- sqrt(var.eta.co)
lwr.co <- 1/(1+exp(-(eta.co-1.96*se.eta.co)))
upr.co <- 1/(1+exp(-(eta.co+1.96*se.eta.co)))

df.ctrl <- data.frame(iv=iv*sd.iv, yhat.co=yhat.co, lwr.co=lwr.co, upr.co=upr.co)

C.ca <- matrix(rep(0,length(iv2)*37), ncol=37)
C.ca[,c(1,2,13,29)] <- cbind(0.5,0.5,iv2,iv2^2)
var.eta.ca <- diag(C.ca %*% V %*% t(C.ca))
se.eta.ca <- sqrt(var.eta.ca)
lwr.ca <- 1/(1+exp(-(eta.ca-1.96*se.eta.ca)))
upr.ca <- 1/(1+exp(-(eta.ca+1.96*se.eta.ca)))

C.ro <- matrix(rep(0,length(iv2)*37), ncol=37)
C.ro[,c(1,2,17,33)] <- cbind(0.5,0.5,iv2,iv2^2)
var.eta.ro <- diag(C.ro %*% V %*% t(C.ro))
se.eta.ro <- sqrt(var.eta.ro)
lwr.ro <- 1/(1+exp(-(eta.ro-1.96*se.eta.ro)))
upr.ro <- 1/(1+exp(-(eta.ro+1.96*se.eta.ro)))

C.tr <- matrix(rep(0,length(iv2)*37), ncol=37)
C.tr[,c(1,2,21,37)] <- cbind(0.5,0.5,iv2,iv2^2)
var.eta.tr <- diag(C.tr %*% V %*% t(C.tr))
se.eta.tr <- sqrt(var.eta.tr)
lwr.tr <- 1/(1+exp(-(eta.tr-1.96*se.eta.tr)))
upr.tr <- 1/(1+exp(-(eta.tr+1.96*se.eta.tr)))

df.other <- data.frame(iv2=iv2*sd.iv, yhat.ca=yhat.ca, lwr=lwr.ca, upr=upr.ca,
                       yhat.ro=yhat.ro, lwr=lwr.ro, upr=upr.ro,
                       yhat.tr=yhat.tr, lwr=lwr.tr, upr=upr.tr)

df.ctrl <- data.frame(iv=iv*sd.iv, yhat.co=yhat.co)
df.other <- data.frame(iv2=iv2*sd.iv, yhat.ca=yhat.ca, yhat.ro=yhat.ro, yhat.tr=yhat.tr)


# Plot 95% confidence bands and points instead of lines
p <- ggplot(data=Ta2017W, aes(x=intervaldays, y=f)) +
  labs(x = "days since start treatment", y = "fraction of cells of tiles tripped upon") +
  theme_bw()+ theme(panel.grid.minor = element_blank()) +
  geom_point(aes(colour=treatment2, group=v.hh), size=1, position=jitter, size=0.75, alpha=0.75) +
  guides(colour = guide_legend(override.aes = list(alpha = 1), title="treatment")) +
  scale_colour_manual(values=man.col) +
  scale_x_continuous(breaks=c(-28,-14,0,14,28,42,56,70), labels=c("-28","-14","0","14","28","42","56","70")) +

  geom_ribbon(data=df.ctrl, mapping=aes(x=iv, y=yhat.co, ymin = lwr.co, ymax = upr.co, group=1),
              fill=man.col[1], alpha = .2) +
  geom_line(data=df.ctrl,  mapping=aes(x=iv, y=yhat.co, group=1),  size=1.1, colour=man.col[1]) +

  geom_ribbon(data=df.other, mapping=aes(x=iv2, y=yhat.ca, ymin = lwr.ca, ymax = upr.ca, group=1),
              fill=man.col[2], alpha = .2) +
  geom_line(data=df.other, mapping=aes(x=iv2, y=yhat.ca, group=1), size=1.1, colour=man.col[2]) +

  geom_ribbon(data=df.other, mapping=aes(x=iv2, y=yhat.ro, ymin = lwr.ro, ymax = upr.ro, group=1),
```

```
              fill=man.col[3], alpha = .2) +
  geom_line(data=df.other, mapping=aes(x=iv2, y=yhat.ro, group=1), size=1.1, colour=man.col[3]) +

  geom_ribbon(data=df.other, mapping=aes(x=iv2, y=yhat.tr, ymin = lwr.tr, ymax = upr.tr, group=1),
              fill=man.col[4], alpha = .2) +
  geom_line(data=df.other, mapping=aes(x=iv2, y=yhat.tr, group=1), size=1.1, colour=man.col[4])
p
```

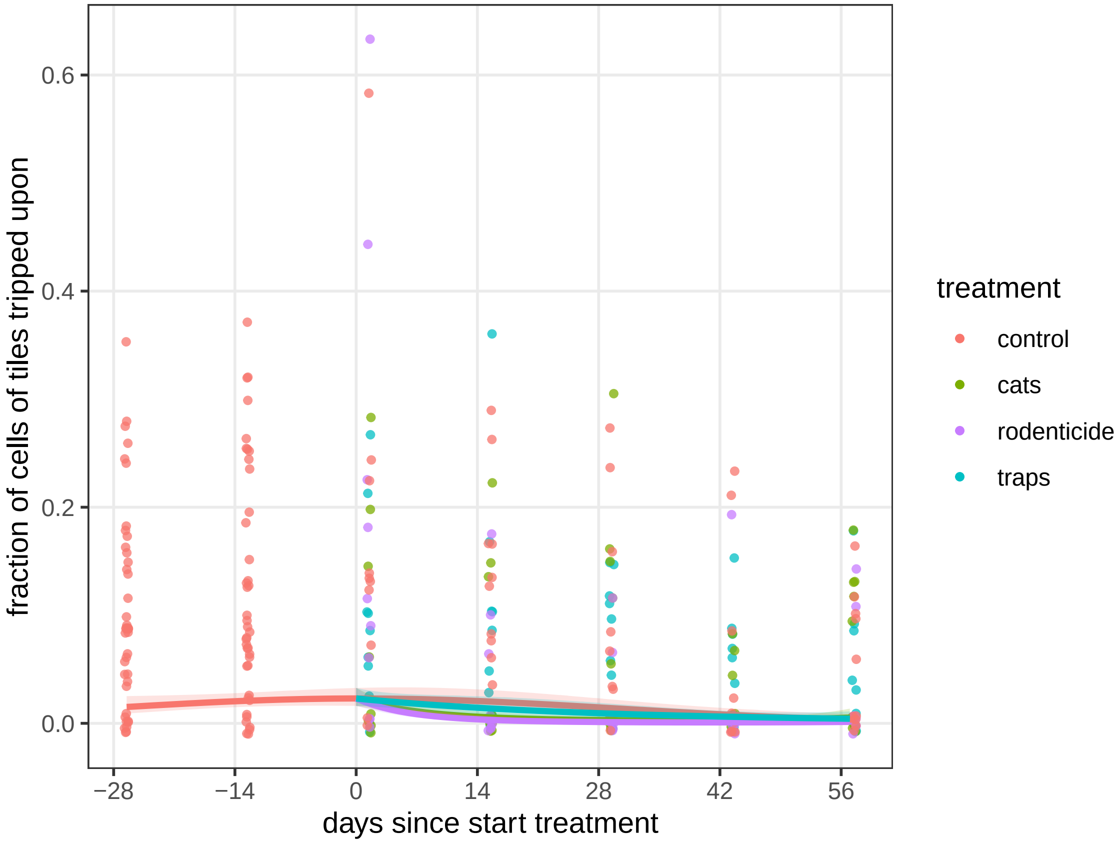


```
cat("\n")
```

## Analyze data on Giving Up Densities (fraction of eaten peanuts per night out of 20 or 25)

The response variable is the number of peanuts eaten out of 20 or 25 given per night. Per household measurements were taken on two consecutive days after the days of the rice measurements.

### Data on GUD: overview plot

```
setwd("C:/MyData/OneDrive - WageningenUR/Data/Inge Krijger/final/GUD")
G <- read.csv("GUD.csv")

G$treatment2 <- relevel(G$treatment2, ref="control")

G$year <- factor(G$year)
G$intervalc <- factor(G$interval2)

sd.iv <- sd(G$intervaldays)
G$iv1 <- (G$intervaldays)/sd(G$intervaldays)
G$iv2 <- G$iv1*G$iv1
G$iv3 <- G$iv1*G$iv1*G$iv1
G$iv4 <- G$iv1*G$iv1*G$iv1*G$iv1

G$YS <- factor(paste(G$year, G$season, sep="."), levels=c("2016.wet","2016.dry","2017.wet","2017.dry"))
# Note: order of levels of T$YS is now: "2016.wet" "2016.dry" "2017.wet" "2017.dry"

head(G)
```

```
##   interval village year hh       date np kp remarks interval2 intervaldays
## 1        1       A 2016  1 06/06/2016 25  7                -3          -35
## 2        1       A 2016  2 06/06/2016 25 19                -3          -35
## 3        1       A 2016  3 06/06/2016 25  9                -3          -35
## 4        1       A 2016  4 06/06/2016 25 12                -3          -35
## 5        1       A 2016  5 06/06/2016 25  0                -3          -35
## 6        1       A 2016  6 06/06/2016 25  2                -3          -35
##   treatment treatment2 season       YS twodayperiod    date.nw v.hh
## 1     traps    control    wet 2016.wet            1 07/06/2016  A.1
## 2     traps    control    wet 2016.wet            1 07/06/2016  A.2
## 3     traps    control    wet 2016.wet            1 07/06/2016  A.3
## 4     traps    control    wet 2016.wet            1 07/06/2016  A.4
## 5     traps    control    wet 2016.wet            1 07/06/2016  A.5
## 6     traps    control    wet 2016.wet            1 07/06/2016  A.6
##   intervalc       iv1      iv2       iv3      iv4
## 1        -3 -1.180566 1.393737 -1.645399 1.942503
## 2        -3 -1.180566 1.393737 -1.645399 1.942503
## 3        -3 -1.180566 1.393737 -1.645399 1.942503
## 4        -3 -1.180566 1.393737 -1.645399 1.942503
## 5        -3 -1.180566 1.393737 -1.645399 1.942503
## 6        -3 -1.180566 1.393737 -1.645399 1.942503
```

```
# Take sum over two consecutive days per household
Ga <- summaryBy(kp + np + intervaldays ~ village + year + season + YS + treatment2 + interval + v.hh + date,
                data=G, FUN=sum, na.rm=TRUE )

Ga$f <- Ga$kp/Ga$np
Ga$intervaldays <- Ga$intervaldays.sum/2

# Overview plot of GUD
ggplot(data=Ga, aes(x=intervaldays, y=f, colour=treatment2, group=v.hh)) + theme_bw()+
  theme(panel.grid.minor = element_blank()) +
  geom_line(size=0.3, alpha=0.35) + facet_wrap(~YS) +
```

```
scale_x_continuous(breaks=c(-28,-14,0,14,28,42,56,70), labels=c("-28","-14","0","14","28","42","56","70")) +
labs(x = "days since start treatment", y = "Giving up densities") +
guides(colour = guide_legend(override.aes = list(alpha = 1), title="treatment")) +
scale_color_manual(values=man.col)
```

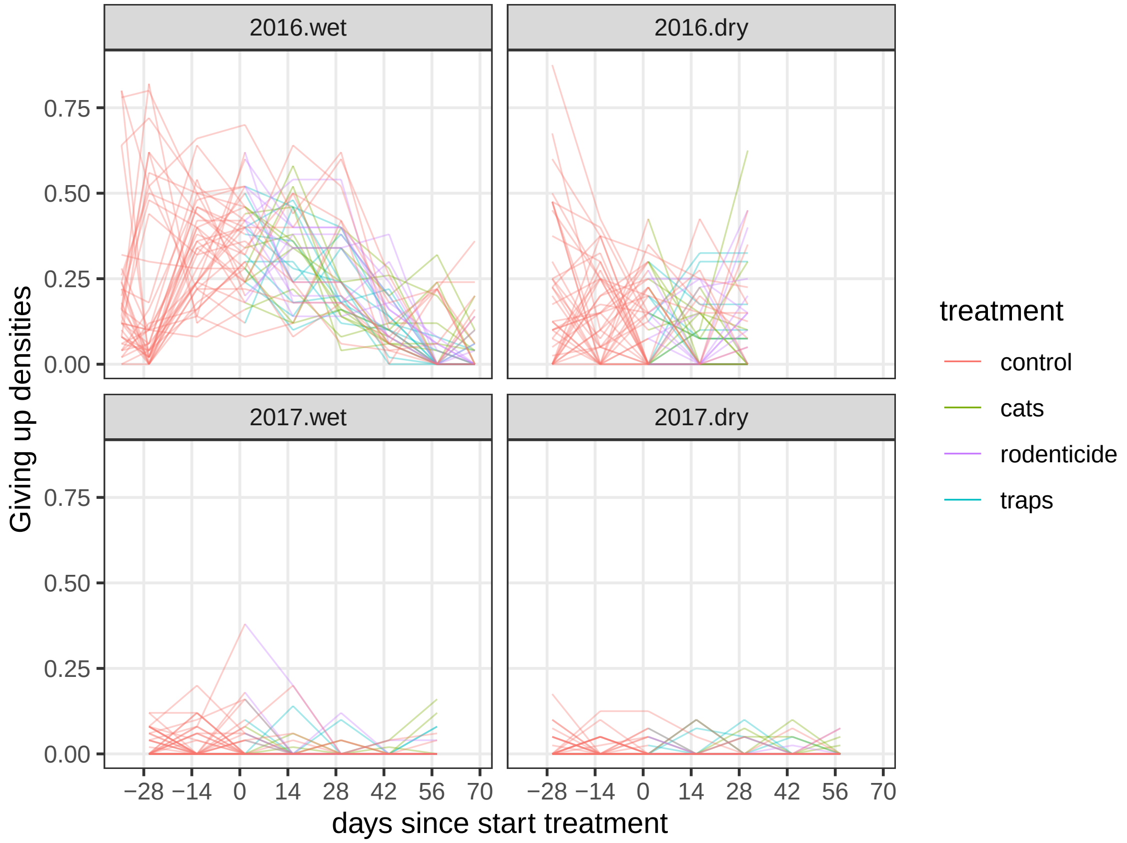


### Data on GUD: generalized linear mixed model analysis of GUDs

We fit a generalized linear mixed model for the number of eaten peanuts (out of 20 or 25) over night, assuming a binomial distribution. The fixed part of the model is the same as for rice loss. In the random part an observation level random effect is added to handle binomial overdispersion.

```
# Quadratic model, binomial overdispersion (random term with date.nw)
#Gglmero<- glmer(cbind(kp,np-kp) ~
#          -1 + year:season + year:season:treatment2:iv1 + year:season:treatment2:iv2
#           + (1 | village:year) + (1 | village:year:intervalc) + (1 | village:year:v.hh:date.nw)
#           + (1 + iv1 + iv2 | year:v.hh),
#           family=binomial(link=logit), data=G,
#           control = glmerControl(optimizer="bobyqa", optCtrl = list(maxfun = 1000000)))
#saveRDS(Gglmero, "C:/MyData/OneDrive - WageningenUR/Data/Inge Krijger/final/GUD/Gglmero")
Gglmero <- readRDS("C:/MyData/OneDrive - WageningenUR/Data/Inge Krijger/final/GUD/Gglmero")

# Cubic model
#Gglmero.iv3 <- glmer(cbind(kp,np-kp) ~
#                -1 + year:season + year:season:treatment2:iv1 + year:season:treatment2:iv2
#                + year:season:treatment2:iv3
#                + (1 | village:year) + (1 | village:year:intervalc) + (1 | village:year:v.hh:date.nw)
#                + (1 + iv1 + iv2 + iv3 | year:v.hh),
#                family=binomial(link=logit), data=G,
#                control = glmerControl(optimizer="nloptwrap", optCtrl = list(maxfun = 1000000)))
#saveRDS(Gglmero.iv3, "C:/MyData/OneDrive - WageningenUR/Data/Inge Krijger/final/GUD/Gglmeroiv3")
Gglmero.iv3 <- readRDS("C:/MyData/OneDrive - WageningenUR/Data/Inge Krijger/final/GUD/Gglmeroiv3")
```

```r
anova(Gglmero, Gglmero.iv3)
```

```
## Data: G
## Models:
## Gglmero: cbind(kp, np - kp) ~ -1 + year:season + year:season:treatment2:iv1 +
## Gglmero:     year:season:treatment2:iv2 + (1 | village:year) + (1 | village:year:intervalc) +
## Gglmero:     (1 | village:year:v.hh:date.nw) + (1 + iv1 + iv2 | year:v.hh)
## Gglmero.iv3: cbind(kp, np - kp) ~ -1 + year:season + year:season:treatment2:iv1 +
## Gglmero.iv3:     year:season:treatment2:iv2 + year:season:treatment2:iv3 +
## Gglmero.iv3:     (1 | village:year) + (1 | village:year:intervalc) + (1 |
## Gglmero.iv3:     village:year:v.hh:date.nw) + (1 + iv1 + iv2 + iv3 | year:v.hh)
##             Df    AIC    BIC  logLik deviance  Chisq Chi Df Pr(>Chisq)
## Gglmero     45 6651.1 6908.1 -3280.6   6561.1
## Gglmero.iv3 65 6681.8 7053.0 -3275.9   6551.8 9.3489     20     0.9785
```

```r
# Model with cubic terms (both in fixed and random part) not better

# We continue with the quadratic model; quadratic random coefficients; binomial overdispersion
summary(Gglmero)
```

```
## Generalized linear mixed model fit by maximum likelihood (Laplace
##   Approximation) [glmerMod]
##  Family: binomial  ( logit )
## Formula:
## cbind(kp, np - kp) ~ -1 + year:season + year:season:treatment2:iv1 +
##     year:season:treatment2:iv2 + (1 | village:year) + (1 | village:year:intervalc) +
##     (1 | village:year:v.hh:date.nw) + (1 + iv1 + iv2 | year:v.hh)
##    Data: G
## Control:
## glmerControl(optimizer = "bobyqa", optCtrl = list(maxfun = 1e+06))
##
##      AIC      BIC   logLik deviance df.resid
##   6651.1   6908.1  -3280.6   6561.1     2188
##
## Scaled residuals:
##      Min       1Q   Median       3Q      Max
## -0.81783 -0.25222 -0.14692  0.04471  1.27853
##
## Random effects:
##  Groups                    Name        Variance  Std.Dev.  Corr
##  village:year:v.hh:date.nw (Intercept) 4.523e+00 2.127e+00
##  year:v.hh                 (Intercept) 4.866e-02 2.206e-01
##                            iv1         2.887e-01 5.373e-01 -1.00
##                            iv2         1.118e-02 1.057e-01  0.91 -0.93
##  village:year:intervalc    (Intercept) 1.840e-15 4.290e-08
##  village:year              (Intercept) 1.039e-16 1.019e-08
## Number of obs: 2233, groups:
## village:year:v.hh:date.nw, 2228; year:v.hh, 160; village:year:intervalc, 112; village:year, 16
##
## Fixed effects:
##                                         Estimate Std. Error z value
## year2016:seasondry                       -3.3425     0.2520 -13.266
## year2017:seasondry                       -6.5881     0.3349 -19.669
## year2016:seasonwet                       -0.7978     0.1675  -4.764
## year2017:seasonwet                       -5.5285     0.2716 -20.356
## year2016:seasondry:treatment2control:iv1 -0.3586     0.2919  -1.228
## year2017:seasondry:treatment2control:iv1 -0.4285     0.3570  -1.200
## year2016:seasonwet:treatment2control:iv1  0.4557     0.1712   2.662
## year2017:seasonwet:treatment2control:iv1 -0.2852     0.3012  -0.947
## year2016:seasondry:treatment2cats:iv1    -0.4453     2.4841  -0.179
```

```
## year2017:seasondry:treatment2cats:iv1          0.9406     1.3876   0.678
## year2016:seasonwet:treatment2cats:iv1         -0.4492     0.6971  -0.644
## year2017:seasonwet:treatment2cats:iv1         -3.0280     1.4788  -2.048
## year2016:seasondry:treatment2rodenticide:iv1  -4.4730     2.5593  -1.748
## year2017:seasondry:treatment2rodenticide:iv1  -0.9474     1.7175  -0.552
## year2016:seasonwet:treatment2rodenticide:iv1   0.9093     0.7001   1.299
## year2017:seasonwet:treatment2rodenticide:iv1  -2.0578     1.5157  -1.358
## year2016:seasondry:treatment2traps:iv1        -2.5117     2.5877  -0.971
## year2017:seasondry:treatment2traps:iv1         1.3156     1.7585   0.748
## year2016:seasonwet:treatment2traps:iv1         0.7238     0.7605   0.952
## year2017:seasonwet:treatment2traps:iv1        -2.5791     1.4438  -1.786
## year2016:seasondry:treatment2control:iv2       0.6366     0.4330   1.470
## year2017:seasondry:treatment2control:iv2       0.1098     0.3202   0.343
## year2016:seasonwet:treatment2control:iv2      -0.8850     0.1196  -7.402
## year2017:seasonwet:treatment2control:iv2      -0.2581     0.2693  -0.958
## year2016:seasondry:treatment2cats:iv2         -0.1815     2.5628  -0.071
## year2017:seasondry:treatment2cats:iv2         -0.5032     0.8027  -0.627
## year2016:seasonwet:treatment2cats:iv2         -0.5407     0.3488  -1.550
## year2017:seasonwet:treatment2cats:iv2          1.3953     0.8297   1.682
## year2016:seasondry:treatment2rodenticide:iv2   5.1701     2.5935   1.994
## year2017:seasondry:treatment2rodenticide:iv2   0.3624     0.9843   0.368
## year2016:seasonwet:treatment2rodenticide:iv2  -1.3629     0.3604  -3.782
## year2017:seasonwet:treatment2rodenticide:iv2   0.5935     0.8996   0.660
## year2016:seasondry:treatment2traps:iv2         1.7864     2.6562   0.673
## year2017:seasondry:treatment2traps:iv2        -1.2329     1.2197  -1.011
## year2016:seasonwet:treatment2traps:iv2        -1.5819     0.4179  -3.786
## year2017:seasonwet:treatment2traps:iv2         1.0420     0.8181   1.274
##                                              Pr(>|z|)
## year2016:seasondry                             < 2e-16
## year2017:seasondry                             < 2e-16
## year2016:seasonwet                            1.90e-06
## year2017:seasonwet                             < 2e-16
## year2016:seasondry:treatment2control:iv1      0.219328
## year2017:seasondry:treatment2control:iv1      0.230066
## year2016:seasonwet:treatment2control:iv1      0.007768
## year2017:seasonwet:treatment2control:iv1      0.343732
## year2016:seasondry:treatment2cats:iv1         0.857742
## year2017:seasondry:treatment2cats:iv1         0.497840
## year2016:seasonwet:treatment2cats:iv1         0.519276
## year2017:seasonwet:treatment2cats:iv1         0.040594
## year2016:seasondry:treatment2rodenticide:iv1  0.080502
## year2017:seasondry:treatment2rodenticide:iv1  0.581233
## year2016:seasonwet:treatment2rodenticide:iv1  0.193994
## year2017:seasonwet:treatment2rodenticide:iv1  0.174583
## year2016:seasondry:treatment2traps:iv1        0.331723
## year2017:seasondry:treatment2traps:iv1        0.454392
## year2016:seasonwet:treatment2traps:iv1        0.341212
## year2017:seasonwet:treatment2traps:iv1        0.074049
## year2016:seasondry:treatment2control:iv2      0.141534
## year2017:seasondry:treatment2control:iv2      0.731651
## year2016:seasonwet:treatment2control:iv2      1.34e-13
## year2017:seasonwet:treatment2control:iv2      0.337852
## year2016:seasondry:treatment2cats:iv2         0.943526
## year2017:seasondry:treatment2cats:iv2         0.530689
## year2016:seasonwet:treatment2cats:iv2         0.121145
## year2017:seasonwet:treatment2cats:iv2         0.092613
## year2016:seasondry:treatment2rodenticide:iv2  0.046206
## year2017:seasondry:treatment2rodenticide:iv2  0.712734
## year2016:seasonwet:treatment2rodenticide:iv2  0.000156
## year2017:seasonwet:treatment2rodenticide:iv2  0.509428
```

```
## year2016:seasondry:treatment2traps:iv2       0.501239
## year2017:seasondry:treatment2traps:iv2       0.312132
## year2016:seasonwet:treatment2traps:iv2       0.000153
## year2017:seasonwet:treatment2traps:iv2       0.202758
```

```
##
## Correlation matrix not shown by default, as p = 36 > 12.
## Use print(x, correlation=TRUE)  or
##     vcov(x)        if you need it
```

```
## convergence code: 0
## boundary (singular) fit: see ?isSingular
```

```
# Residual analysis using DHARMa
simulationOutput <- simulateResiduals(fittedModel = Gglmero, n=5000, plot=TRUE)
```

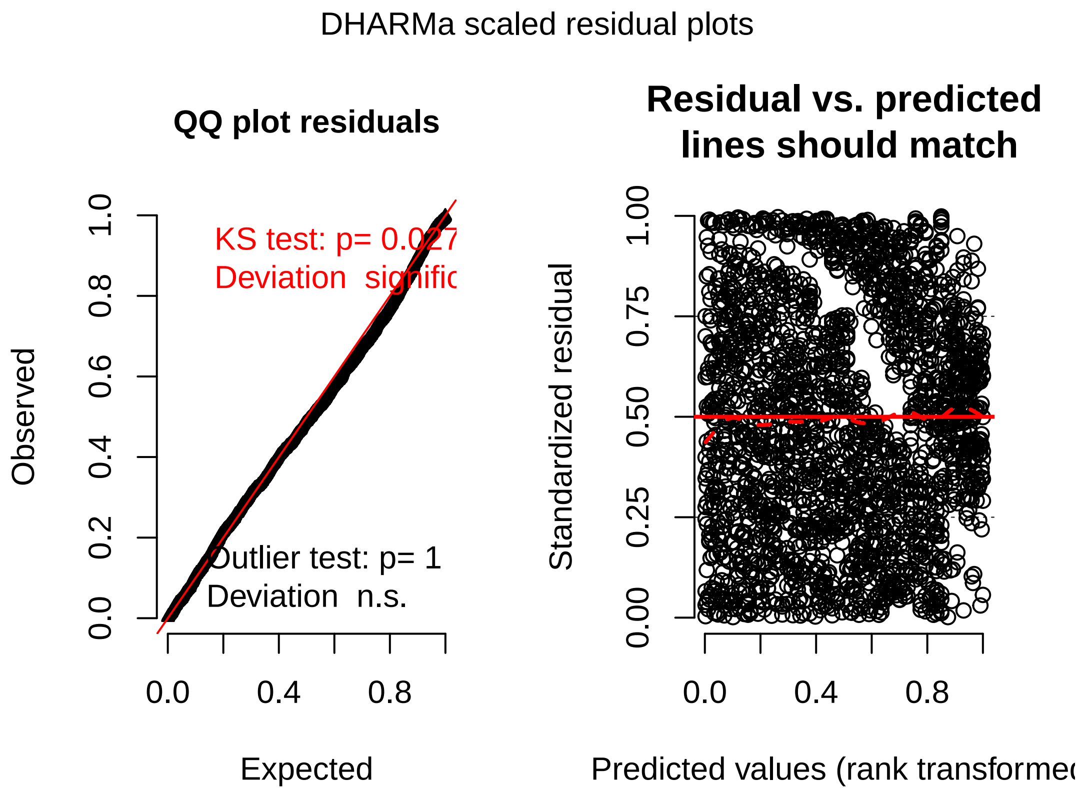


```
# We again find slight deviations in the residual patterns.
# Again, the small P-value for the KS test is not too disturbing, given the size of the dataset (n=2240)


# Compare treatment time trends between year-season combinations:
#Gglmero.YS <- glmer(cbind(kp,np-kp) ~ treatment2:iv1 + treatment2:iv2
#                + (1 | village:year) + (1 | village:year:intervalc) + (1 | village:year:v.hh:date.nw)
#                + (1 + iv1 + iv2 | year:v.hh),
#                family=binomial(link=logit), data=G,
#                control = glmerControl(optimizer="nloptwrap", optCtrl = list(maxfun = 1000000)))
#saveRDS(Gglmero.YS, "C:/MyData/OneDrive - WageningenUR/Data/Inge Krijger/final/GUD/GglmeroYS")
Gglmero.YS <- readRDS("C:/MyData/OneDrive - WageningenUR/Data/Inge Krijger/final/GUD/GglmeroYS")
anova(Gglmero, Gglmero.YS)
```

```
## Data: G
## Models:
## Gglmero.YS: cbind(kp, np - kp) ~ treatment2:iv1 + treatment2:iv2 + (1 | village:year) +
## Gglmero.YS:     (1 | village:year:intervalc) + (1 | village:year:v.hh:date.nw) +
```

```
## Gglmero.YS:     (1 + iv1 + iv2 | year:v.hh)
## Gglmero: cbind(kp, np - kp) ~ -1 + year:season + year:season:treatment2:iv1 +
## Gglmero:     year:season:treatment2:iv2 + (1 | village:year) + (1 | village:year:intervalc) +
## Gglmero:     (1 | village:year:v.hh:date.nw) + (1 + iv1 + iv2 | year:v.hh)
##            Df    AIC    BIC  logLik deviance  Chisq Chi Df Pr(>Chisq)
## Gglmero.YS 18 6821.5 6924.3 -3392.7   6785.5
## Gglmero    45 6651.1 6908.1 -3280.6   6561.1 224.34     27  < 2.2e-16
```

```
# In this test, the coefficients for intercepts are compared (3 df),
# coefficients for linear terms per treatment (so, 4x3=12 df) and
# for quadratic terms (4x3=12 df) are compared. In total 27 df.
# Time trends are significantly different among year-season combinations.

# Now pairwise comparisons between year-season combinations:

# Order of R$YS is: "2016.wet" "2016.dry" "2017.wet" "2017.dry"

# 2016-wet versus 2016-dry:
G$YS12 <- G$YS; levels(G$YS12) <- c(1,1,3,4)
#Gglmero.12 <- glmer(cbind(kp,np-kp) ~ YS12 + YS12:treatment2:iv1 + YS12:treatment2:iv2
#                    + (1 | village:year) + (1 | village:year:intervalc)  + (1 | village:year:v.hh:date.nw)
#                    + (1 + iv1 + iv2 | year:v.hh),
#                    family=binomial(link=logit), data=G,
#                    control = glmerControl(optimizer="nloptwrap", optCtrl = list(maxfun = 1000000)))
#saveRDS(Gglmero.12, "C:/MyData/OneDrive - WageningenUR/Data/Inge Krijger/final/GUD/Gglmero12")
Gglmero.12 <- readRDS("C:/MyData/OneDrive - WageningenUR/Data/Inge Krijger/final/GUD/Gglmero12")

anova(Gglmero, Gglmero.12)
```

```
## Data: G
## Models:
## Gglmero.12: cbind(kp, np - kp) ~ YS12 + YS12:treatment2:iv1 + YS12:treatment2:iv2 +
## Gglmero.12:     (1 | village:year) + (1 | village:year:intervalc) + (1 |
## Gglmero.12:     village:year:v.hh:date.nw) + (1 + iv1 + iv2 | year:v.hh)
## Gglmero: cbind(kp, np - kp) ~ -1 + year:season + year:season:treatment2:iv1 +
## Gglmero:     year:season:treatment2:iv2 + (1 | village:year) + (1 | village:year:intervalc) +
## Gglmero:     (1 | village:year:v.hh:date.nw) + (1 + iv1 + iv2 | year:v.hh)
##            Df    AIC    BIC  logLik deviance  Chisq Chi Df Pr(>Chisq)
## Gglmero.12 36 6748.9 6954.5 -3338.4   6676.9
## Gglmero    45 6651.1 6908.1 -3280.6   6561.1 115.78      9  < 2.2e-16
```

```
# Time trends in 2016-wet and 2016-dry are significantly different

# 2016-wet versus 2017-wet:
G$YS13 <- G$YS; levels(G$YS13) <- c(1,2,1,4)
#Gglmero.13 <- glmer(cbind(kp,np-kp) ~ YS13 + YS13:treatment2:iv1 + YS13:treatment2:iv2
#              + (1 | village:year) + (1 | village:year:intervalc)  + (1 | village:year:v.hh:date.nw)
#              + (1 + iv1 + iv2 | year:v.hh),
#              family=binomial(link=logit), data=G,
#              control = glmerControl(optimizer="nloptwrap", optCtrl = list(maxfun = 1000000)))
#saveRDS(Gglmero.13, "C:/MyData/OneDrive - WageningenUR/Data/Inge Krijger/final/GUD/Gglmero13")
Gglmero.13 <- readRDS("C:/MyData/OneDrive - WageningenUR/Data/Inge Krijger/final/GUD/Gglmero13")
anova(Gglmero, Gglmero.13)
```

```
## Data: G
## Models:
## Gglmero.13: cbind(kp, np - kp) ~ YS13 + YS13:treatment2:iv1 + YS13:treatment2:iv2 +
## Gglmero.13:     (1 | village:year) + (1 | village:year:intervalc) + (1 |
## Gglmero.13:     village:year:v.hh:date.nw) + (1 + iv1 + iv2 | year:v.hh)
## Gglmero: cbind(kp, np - kp) ~ -1 + year:season + year:season:treatment2:iv1 +
## Gglmero:     year:season:treatment2:iv2 + (1 | village:year) + (1 | village:year:intervalc) +
```

```
## Gglmero:     (1 | village:year:v.hh:date.nw) + (1 + iv1 + iv2 | year:v.hh)
##            Df    AIC    BIC  logLik deviance Chisq Chi Df Pr(>Chisq)
## Gglmero.13 36 6811.9 7017.5 -3370.0   6739.9
## Gglmero    45 6651.1 6908.1 -3280.6   6561.1 178.8      9  < 2.2e-16
```

```
# Time trends in 2016-wet and 2017-wet are significantly different

# 2016-wet versus 2017-dry:
G$YS14 <- G$YS; levels(G$YS14) <- c(1,2,3,1)
#Gglmero.14 <- glmer(cbind(kp,np-kp) ~ YS14 + YS14:treatment2:iv1 + YS14:treatment2:iv2
#              + (1 | village:year) + (1 | village:year:intervalc)  + (1 | village:year:v.hh:date.nw)
#              + (1 + iv1 + iv2 | year:v.hh),
#              family=binomial(link=logit), data=G,
#              control = glmerControl(optimizer="nloptwrap", optCtrl = list(maxfun = 1000000)))
#saveRDS(Gglmero.14, "C:/MyData/OneDrive - WageningenUR/Data/Inge Krijger/final/GUD/Gglmero14")
Gglmero.14 <- readRDS("C:/MyData/OneDrive - WageningenUR/Data/Inge Krijger/final/GUD/Gglmero14")
anova(Gglmero, Gglmero.14)
```

```
## Data: G
## Models:
## Gglmero.14: cbind(kp, np - kp) ~ YS14 + YS14:treatment2:iv1 + YS14:treatment2:iv2 +
## Gglmero.14:     (1 | village:year) + (1 | village:year:intervalc) + (1 |
## Gglmero.14:     village:year:v.hh:date.nw) + (1 + iv1 + iv2 | year:v.hh)
## Gglmero: cbind(kp, np - kp) ~ -1 + year:season + year:season:treatment2:iv1 +
## Gglmero:     year:season:treatment2:iv2 + (1 | village:year) + (1 | village:year:intervalc) +
## Gglmero:     (1 | village:year:v.hh:date.nw) + (1 + iv1 + iv2 | year:v.hh)
##            Df    AIC    BIC  logLik deviance  Chisq Chi Df Pr(>Chisq)
## Gglmero.14 36 6804.1 7009.7 -3366.1   6732.1
## Gglmero    45 6651.1 6908.1 -3280.6   6561.1 171.01      9  < 2.2e-16
```

```
# Time trends in 2016-wet and 2017-dry are significantly different

# 2016-dry versus 2017-wet:
G$YS23 <- G$YS; levels(G$YS23) <- c(1,2,2,4)
#Gglmero.23 <- glmer(cbind(kp,np-kp) ~ YS23 + YS23:treatment2:iv1 + YS23:treatment2:iv2
#              + (1 | village:year) + (1 | village:year:intervalc)  + (1 | village:year:v.hh:date.nw)
#              + (1 + iv1 + iv2 | year:v.hh),
#              family=binomial(link=logit), data=G,
#              control = glmerControl(optimizer="nloptwrap", optCtrl = list(maxfun = 1000000)))
#saveRDS(Gglmero.23, "C:/MyData/OneDrive - WageningenUR/Data/Inge Krijger/final/GUD/Gglmero23")
Gglmero.23 <- readRDS("C:/MyData/OneDrive - WageningenUR/Data/Inge Krijger/final/GUD/Gglmero23")
anova(Gglmero, Gglmero.23)
```

```
## Data: G
## Models:
## Gglmero.23: cbind(kp, np - kp) ~ YS23 + YS23:treatment2:iv1 + YS23:treatment2:iv2 +
## Gglmero.23:     (1 | village:year) + (1 | village:year:intervalc) + (1 |
## Gglmero.23:     village:year:v.hh:date.nw) + (1 + iv1 + iv2 | year:v.hh)
## Gglmero: cbind(kp, np - kp) ~ -1 + year:season + year:season:treatment2:iv1 +
## Gglmero:     year:season:treatment2:iv2 + (1 | village:year) + (1 | village:year:intervalc) +
## Gglmero:     (1 | village:year:v.hh:date.nw) + (1 + iv1 + iv2 | year:v.hh)
##            Df    AIC    BIC  logLik deviance  Chisq Chi Df Pr(>Chisq)
## Gglmero.23 36 6771.5 6977.1 -3349.8   6699.5
## Gglmero    45 6651.1 6908.1 -3280.6   6561.1 138.43      9  < 2.2e-16
```

```
# Time trends in 2016-dry and 2017-wet are significantly different

# 2016-dry versus 2017-dry:
G$YS24 <- G$YS; levels(G$YS24) <- c(1,2,3,2)
#Gglmero.24 <- glmer(cbind(kp,np-kp) ~ YS24 + YS24:treatment2:iv1 + YS24:treatment2:iv2
#              + (1 | village:year) + (1 | village:year:intervalc)  + (1 | village:year:v.hh:date.nw)
#              + (1 + iv1 + iv2 | year:v.hh),
```

```
#              family=binomial(link=logit), data=G,
#              control = glmerControl(optimizer="nloptwrap", optCtrl = list(maxfun = 1000000)))
#saveRDS(Gglmero.24, "C:/MyData/OneDrive - WageningenUR/Data/Inge Krijger/final/GUD/Gglmero24")
Gglmero.24 <- readRDS("C:/MyData/OneDrive - WageningenUR/Data/Inge Krijger/final/GUD/Gglmero24")
anova(Gglmero, Gglmero.24)
```

```
## Data: G
## Models:
## Gglmero.24: cbind(kp, np - kp) ~ YS24 + YS24:treatment2:iv1 + YS24:treatment2:iv2 +
## Gglmero.24:     (1 | village:year) + (1 | village:year:intervalc) + (1 |
## Gglmero.24:     village:year:v.hh:date.nw) + (1 + iv1 + iv2 | year:v.hh)
## Gglmero: cbind(kp, np - kp) ~ -1 + year:season + year:season:treatment2:iv1 +
## Gglmero:     year:season:treatment2:iv2 + (1 | village:year) + (1 | village:year:intervalc) +
## Gglmero:     (1 | village:year:v.hh:date.nw) + (1 + iv1 + iv2 | year:v.hh)
##            Df    AIC    BIC  logLik deviance Chisq Chi Df Pr(>Chisq)
## Gglmero.24 36 6777.0 6982.6 -3352.5   6705.0
## Gglmero    45 6651.1 6908.1 -3280.6   6561.1 143.9      9  < 2.2e-16
```

```
# Time trends in 2016-dry and 2017-dry are significantly different

# 2017-wet versus 2017-dry:
G$YS34 <- G$YS; levels(G$YS34) <- c(1,2,3,3)
#Gglmero.34 <- glmer(cbind(kp,np-kp) ~ YS34 + YS34:treatment2:iv1 + YS34:treatment2:iv2
#              + (1 | village:year) + (1 | village:year:intervalc)  + (1 | village:year:v.hh:date.nw)
#              + (1 + iv1 + iv2 | year:v.hh),
#              family=binomial(link=logit), data=G,
#              control = glmerControl(optimizer="nloptwrap", optCtrl = list(maxfun = 1000000)))
#saveRDS(Gglmero.34, "C:/MyData/OneDrive - WageningenUR/Data/Inge Krijger/final/GUD/Gglmero34")
Gglmero.34 <- readRDS("C:/MyData/OneDrive - WageningenUR/Data/Inge Krijger/final/GUD/Gglmero34")
anova(Gglmero, Gglmero.34)
```

```
## Data: G
## Models:
## Gglmero.34: cbind(kp, np - kp) ~ YS34 + YS34:treatment2:iv1 + YS34:treatment2:iv2 +
## Gglmero.34:     (1 | village:year) + (1 | village:year:intervalc) + (1 |
## Gglmero.34:     village:year:v.hh:date.nw) + (1 + iv1 + iv2 | year:v.hh)
## Gglmero: cbind(kp, np - kp) ~ -1 + year:season + year:season:treatment2:iv1 +
## Gglmero:     year:season:treatment2:iv2 + (1 | village:year) + (1 | village:year:intervalc) +
## Gglmero:     (1 | village:year:v.hh:date.nw) + (1 + iv1 + iv2 | year:v.hh)
##            Df    AIC    BIC  logLik deviance  Chisq Chi Df Pr(>Chisq)
## Gglmero.34 36 6647.3 6852.9 -3287.7   6575.3
## Gglmero    45 6651.1 6908.1 -3280.6   6561.1 14.235      9     0.1142
```

```
# Time trends in 2017-wet and 2017-dry are not significantly different

# Compare time trends between treatments per year-season combination:
# Within year 2016-dry:
C1 <- rbind(c(0, 0,0,0,  1,0,0,0, -1,0,0,0, 0,0,0,0, 0,0,0,0,  0,0,0,0, 0,0,0,0, 0,0,0,0, 0,0,0,0),
            c(0, 0,0,0,  1,0,0,0, 0,0,0,0, -1,0,0,0, 0,0,0,0,  0,0,0,0, 0,0,0,0, 0,0,0,0, 0,0,0,0),
            c(0, 0,0,0,  1,0,0,0, 0,0,0,0, 0,0,0,0, -1,0,0,0,  0,0,0,0, 0,0,0,0, 0,0,0,0, 0,0,0,0),

            c(0, 0,0,0,  0,0,0,0, 0,0,0,0, 0,0,0,0, 0,0,0,0,  1,0,0,0, -1,0,0,0, 0,0,0,0, 0,0,0,0),
            c(0, 0,0,0,  0,0,0,0, 0,0,0,0, 0,0,0,0, 0,0,0,0,  1,0,0,0, 0,0,0,0, -1,0,0,0, 0,0,0,0),
            c(0, 0,0,0,  0,0,0,0, 0,0,0,0, 0,0,0,0, 0,0,0,0,  1,0,0,0, 0,0,0,0, 0,0,0,0, -1,0,0,0))

linearHypothesis(Gglmero, C1)
```

```
## Linear hypothesis test
##
## Hypothesis:
## year2016:seasondry:treatment2control:iv1 - year2016:seasondry:treatment2cats:iv1 = 0
```

```
## year2016:seasondry:treatment2control:iv1 - year2016:seasondry:treatment2rodenticide:iv1 = 0
## year2016:seasondry:treatment2control:iv1 - year2016:seasondry:treatment2traps:iv1 = 0
## year2016:seasondry:treatment2control:iv2 - year2016:seasondry:treatment2cats:iv2 = 0
## year2016:seasondry:treatment2control:iv2 - year2016:seasondry:treatment2rodenticide:iv2 = 0
## year2016:seasondry:treatment2control:iv2 - year2016:seasondry:treatment2traps:iv2 = 0
##
## Model 1: restricted model
## Model 2: cbind(kp, np - kp) ~ -1 + year:season + year:season:treatment2:iv1 +
##     year:season:treatment2:iv2 + (1 | village:year) + (1 | village:year:intervalc) +
##     (1 | village:year:v.hh:date.nw) + (1 + iv1 + iv2 | year:v.hh)
##
##   Df  Chisq Pr(>Chisq)
## 1
## 2  6 7.5622      0.272
```

```
# No time trend differences between treatments in 2016-dry

# Within year 2017-dry:
C2 <- rbind(c(0, 0,0,0,  0,1,0,0, 0,-1,0,0, 0,0,0,0, 0,0,0,0,  0,0,0,0, 0,0,0,0, 0,0,0,0, 0,0,0,0),
            c(0, 0,0,0,  0,1,0,0, 0,0,0,0, 0,-1,0,0, 0,0,0,0,  0,0,0,0, 0,0,0,0, 0,0,0,0, 0,0,0,0),
            c(0, 0,0,0,  0,1,0,0, 0,0,0,0, 0,0,0,0, 0,-1,0,0,  0,0,0,0, 0,0,0,0, 0,0,0,0, 0,0,0,0),

            c(0, 0,0,0,  0,0,0,0, 0,0,0,0, 0,0,0,0, 0,0,0,0,  0,1,0,0, 0,-1,0,0, 0,0,0,0, 0,0,0,0),
            c(0, 0,0,0,  0,0,0,0, 0,0,0,0, 0,0,0,0, 0,0,0,0,  0,1,0,0, 0,0,0,0, 0,-1,0,0, 0,0,0,0),
            c(0, 0,0,0,  0,0,0,0, 0,0,0,0, 0,0,0,0, 0,0,0,0,  0,1,0,0, 0,0,0,0, 0,0,0,0, 0,-1,0,0))

linearHypothesis(Gglmero, C2)
```

```
## Linear hypothesis test
##
## Hypothesis:
## year2017:seasondry:treatment2control:iv1 - year2017:seasondry:treatment2cats:iv1 = 0
## year2017:seasondry:treatment2control:iv1 - year2017:seasondry:treatment2rodenticide:iv1 = 0
## year2017:seasondry:treatment2control:iv1 - year2017:seasondry:treatment2traps:iv1 = 0
## year2017:seasondry:treatment2control:iv2 - year2017:seasondry:treatment2cats:iv2 = 0
## year2017:seasondry:treatment2control:iv2 - year2017:seasondry:treatment2rodenticide:iv2 = 0
## year2017:seasondry:treatment2control:iv2 - year2017:seasondry:treatment2traps:iv2 = 0
##
## Model 1: restricted model
## Model 2: cbind(kp, np - kp) ~ -1 + year:season + year:season:treatment2:iv1 +
##     year:season:treatment2:iv2 + (1 | village:year) + (1 | village:year:intervalc) +
##     (1 | village:year:v.hh:date.nw) + (1 + iv1 + iv2 | year:v.hh)
##
##   Df  Chisq Pr(>Chisq)
## 1
## 2  6 2.9281     0.8178
```

```
# No time trend differences between treatments in 2017-dry

# Within year 2016-wet:
C3 <- rbind(c(0, 0,0,0,  0,0,1,0, 0,0,-1,0, 0,0,0,0, 0,0,0,0,  0,0,0,0, 0,0,0,0, 0,0,0,0, 0,0,0,0),
            c(0, 0,0,0,  0,0,1,0, 0,0,0,0, 0,0,-1,0, 0,0,0,0,  0,0,0,0, 0,0,0,0, 0,0,0,0, 0,0,0,0),
            c(0, 0,0,0,  0,0,1,0, 0,0,0,0, 0,0,0,0, 0,0,-1,0,  0,0,0,0, 0,0,0,0, 0,0,0,0, 0,0,0,0),

            c(0, 0,0,0,  0,0,0,0, 0,0,0,0, 0,0,0,0, 0,0,0,0,  0,0,1,0, 0,0,-1,0, 0,0,0,0, 0,0,0,0),
            c(0, 0,0,0,  0,0,0,0, 0,0,0,0, 0,0,0,0, 0,0,0,0,  0,0,1,0, 0,0,0,0, 0,0,-1,0, 0,0,0,0),
            c(0, 0,0,0,  0,0,0,0, 0,0,0,0, 0,0,0,0, 0,0,0,0,  0,0,1,0, 0,0,0,0, 0,0,0,0, 0,0,-1,0))

linearHypothesis(Gglmero, C3)
```

```
## Linear hypothesis test
##
```

```
## Hypothesis:
## year2016:seasonwet:treatment2control:iv1 - year2016:seasonwet:treatment2cats:iv1 = 0
## year2016:seasonwet:treatment2control:iv1 - year2016:seasonwet:treatment2rodenticide:iv1 = 0
## year2016:seasonwet:treatment2control:iv1 - year2016:seasonwet:treatment2traps:iv1 = 0
## year2016:seasonwet:treatment2control:iv2 - year2016:seasonwet:treatment2cats:iv2 = 0
## year2016:seasonwet:treatment2control:iv2 - year2016:seasonwet:treatment2rodenticide:iv2 = 0
## year2016:seasonwet:treatment2control:iv2 - year2016:seasonwet:treatment2traps:iv2 = 0
##
## Model 1: restricted model
## Model 2: cbind(kp, np - kp) ~ -1 + year:season + year:season:treatment2:iv1 +
##     year:season:treatment2:iv2 + (1 | village:year) + (1 | village:year:intervalc) +
##     (1 | village:year:v.hh:date.nw) + (1 + iv1 + iv2 | year:v.hh)
##
##   Df  Chisq Pr(>Chisq)
## 1
## 2  6 16.505    0.01129
```

```
# Time trend differences between treatments in 2016-wet

# Within year 2017-wet:
C4 <- rbind(c(0, 0,0,0,  0,0,0,1, 0,0,0,-1, 0,0,0,0, 0,0,0,0,  0,0,0,0, 0,0,0,0, 0,0,0,0, 0,0,0,0),
            c(0, 0,0,0,  0,0,0,1, 0,0,0,0, 0,0,0,-1, 0,0,0,0,  0,0,0,0, 0,0,0,0, 0,0,0,0, 0,0,0,0),
            c(0, 0,0,0,  0,0,0,1, 0,0,0,0, 0,0,0,0, 0,0,0,-1,  0,0,0,0, 0,0,0,0, 0,0,0,0, 0,0,0,0),

            c(0, 0,0,0,  0,0,0,0, 0,0,0,0, 0,0,0,0, 0,0,0,0,  0,0,0,1, 0,0,0,-1, 0,0,0,0, 0,0,0,0),
            c(0, 0,0,0,  0,0,0,0, 0,0,0,0, 0,0,0,0, 0,0,0,0,  0,0,0,1, 0,0,0,0, 0,0,0,-1, 0,0,0,0),
            c(0, 0,0,0,  0,0,0,0, 0,0,0,0, 0,0,0,0, 0,0,0,0,  0,0,0,1, 0,0,0,0, 0,0,0,0, 0,0,0,-1))

linearHypothesis(Gglmero, C4)
```

```
## Linear hypothesis test
##
## Hypothesis:
## year2017:seasonwet:treatment2control:iv1 - year2017:seasonwet:treatment2cats:iv1 = 0
## year2017:seasonwet:treatment2control:iv1 - year2017:seasonwet:treatment2rodenticide:iv1 = 0
## year2017:seasonwet:treatment2control:iv1 - year2017:seasonwet:treatment2traps:iv1 = 0
## year2017:seasonwet:treatment2control:iv2 - year2017:seasonwet:treatment2cats:iv2 = 0
## year2017:seasonwet:treatment2control:iv2 - year2017:seasonwet:treatment2rodenticide:iv2 = 0
## year2017:seasonwet:treatment2control:iv2 - year2017:seasonwet:treatment2traps:iv2 = 0
##
## Model 1: restricted model
## Model 2: cbind(kp, np - kp) ~ -1 + year:season + year:season:treatment2:iv1 +
##     year:season:treatment2:iv2 + (1 | village:year) + (1 | village:year:intervalc) +
##     (1 | village:year:v.hh:date.nw) + (1 + iv1 + iv2 | year:v.hh)
##
##   Df  Chisq Pr(>Chisq)
## 1
## 2  6 5.7446     0.4524
```

```
# No time trend differences between treatments in 2017-wet
```

### Data on GUD: Testing pairwise treatment differences at different time points within 2016-wet

```
G1.emm <- emmeans(Gglmero,"treatment2", type="response",
                  at=list(year="2016", season="wet", iv1=14/sd.iv, iv2=(14/sd.iv)^2, iv3=(14/sd.iv)^3))
```

```
## NOTE: Results may be misleading due to involvement in interactions
```

```
pairs(G1.emm)
```

```
##  contrast              odds.ratio    SE  df z.ratio p.value
##  control / cats             1.420 0.380 Inf   1.310  0.5568
```

```
##  control / rodenticide      0.898 0.240 Inf -0.403  0.9778
##  control / traps            1.029 0.293 Inf  0.101  0.9996
##  cats / rodenticide         0.632 0.205 Inf -1.413  0.4910
##  cats / traps               0.725 0.243 Inf -0.961  0.7715
##  rodenticide / traps        1.146 0.385 Inf  0.406  0.9774
##
## P value adjustment: tukey method for comparing a family of 4 estimates
## Tests are performed on the log odds ratio scale
```

```
CLD(G1.emm, Letters=letters)
```

```
##  treatment2   prob     SE  df asymp.LCL asymp.UCL .group
##  cats        0.244 0.0436 Inf     0.169     0.339  a
##  traps       0.308 0.0534 Inf     0.214     0.421  a
##  control     0.314 0.0407 Inf     0.240     0.399  a
##  rodenticide 0.338 0.0529 Inf     0.243     0.448  a
##
## Confidence level used: 0.95
## Intervals are back-transformed from the logit scale
## P value adjustment: tukey method for comparing a family of 4 estimates
## Tests are performed on the log odds ratio scale
## significance level used: alpha = 0.05
```

```
G2.emm <- emmeans(Gglmero,"treatment2", type="response",
                  at=list(year="2016", season="wet", iv1=28/sd.iv, iv2=(28/sd.iv)^2, iv3=(28/sd.iv)^3))
```

```
## NOTE: Results may be misleading due to involvement in interactions
```

```
pairs(G2.emm)
```

```
##  contrast              odds.ratio    SE  df z.ratio p.value
##  control / cats             1.729 0.669 Inf  1.415  0.4901
##  control / rodenticide      0.998 0.382 Inf -0.006  1.0000
##  control / traps            1.445 0.580 Inf  0.918  0.7950
##  cats / rodenticide         0.577 0.263 Inf -1.205  0.6237
##  cats / traps               0.836 0.389 Inf -0.385  0.9805
##  rodenticide / traps        1.448 0.674 Inf  0.796  0.8561
##
## P value adjustment: tukey method for comparing a family of 4 estimates
## Tests are performed on the log odds ratio scale
```

```
CLD(G2.emm, Letters=letters)
```

```
##  treatment2   prob     SE  df asymp.LCL asymp.UCL .group
##  cats        0.154 0.0424 Inf    0.0877     0.256  a
##  traps       0.179 0.0493 Inf    0.1013     0.296  a
##  control     0.239 0.0372 Inf    0.1741     0.319  a
##  rodenticide 0.240 0.0587 Inf    0.1436     0.372  a
##
## Confidence level used: 0.95
## Intervals are back-transformed from the logit scale
## P value adjustment: tukey method for comparing a family of 4 estimates
## Tests are performed on the log odds ratio scale
## significance level used: alpha = 0.05
```

```
G3.emm <- emmeans(Gglmero,"treatment2", type="response",
                  at=list(year="2016", season="wet", iv1=42/sd.iv, iv2=(42/sd.iv)^2, iv3=(42/sd.iv)^3))
```

```
## NOTE: Results may be misleading due to involvement in interactions
```

```
pairs(G3.emm)
```

```
##  contrast              odds.ratio    SE  df z.ratio p.value
##  control / cats              1.81 0.729 Inf  1.464  0.4592
```

```
##  control / rodenticide       1.37 0.548 Inf  0.792  0.8579
##  control / traps             2.77 1.158 Inf  2.437  0.0703
##  cats / rodenticide          0.76 0.337 Inf -0.619  0.9261
##  cats / traps                1.53 0.694 Inf  0.946  0.7802
##  rodenticide / traps         2.02 0.917 Inf  1.545  0.4107
##
## P value adjustment: tukey method for comparing a family of 4 estimates
## Tests are performed on the log odds ratio scale
```

```r
CLD(G3.emm, Letters=letters)
```

```
##  treatment2    prob     SE  df asymp.LCL asymp.UCL .group
##  traps       0.0499 0.0158 Inf    0.0266    0.0916  a
##  cats        0.0745 0.0221 Inf    0.0412    0.1311  a
##  rodenticide 0.0958 0.0275 Inf    0.0538    0.1649  a
##  control     0.1269 0.0268 Inf    0.0829    0.1894  a
##
## Confidence level used: 0.95
## Intervals are back-transformed from the logit scale
## P value adjustment: tukey method for comparing a family of 4 estimates
## Tests are performed on the log odds ratio scale
## significance level used: alpha = 0.05
```

```r
G4.emm <- emmeans(Gglmero,"treatment2", type="response",
                  at=list(year="2016", season="wet", iv1=56/sd.iv, iv2=(56/sd.iv)^2, iv3=(56/sd.iv)^3))
```

```
## NOTE: Results may be misleading due to involvement in interactions
```

```r
pairs(G4.emm)
```

```
##  contrast              odds.ratio    SE  df z.ratio p.value
##  control / cats              1.62 0.754 Inf 1.031   0.7311
##  control / rodenticide       2.34 1.120 Inf 1.769   0.2883
##  control / traps             7.24 3.979 Inf 3.603   0.0018
##  cats / rodenticide          1.44 0.697 Inf 0.761   0.8719
##  cats / traps                4.48 2.416 Inf 2.779   0.0280
##  rodenticide / traps         3.10 1.736 Inf 2.021   0.1801
##
## P value adjustment: tukey method for comparing a family of 4 estimates
## Tests are performed on the log odds ratio scale
```

```r
CLD(G4.emm, Letters=letters)
```

```
##  treatment2     prob      SE  df asymp.LCL asymp.UCL .group
##  traps       0.00622 0.00275 Inf   0.00261    0.0147  a
##  rodenticide 0.01902 0.00679 Inf   0.00942    0.0380  ab
##  cats        0.02724 0.00905 Inf   0.01414    0.0518   b
##  control     0.04332 0.01412 Inf   0.02270    0.0811   b
##
## Confidence level used: 0.95
## Intervals are back-transformed from the logit scale
## P value adjustment: tukey method for comparing a family of 4 estimates
## Tests are performed on the log odds ratio scale
## significance level used: alpha = 0.05
```

```r
G5.emm <- emmeans(Gglmero,"treatment2", type="response",
                  at=list(year="2016", season="wet", iv1=67/sd.iv, iv2=(67/sd.iv)^2, iv3=(67/sd.iv)^3))
```

```
## NOTE: Results may be misleading due to involvement in interactions
```

```r
pairs(G5.emm)
```

```
##  contrast              odds.ratio     SE  df z.ratio p.value
##  control / cats              1.33  0.911 Inf 0.418   0.9754
```

```
##  control / rodenticide       4.12  2.968 Inf 1.964   0.2017
##  control / traps            19.16 16.570 Inf 3.415   0.0036
##  cats / rodenticide          3.09  2.337 Inf 1.494   0.4409
##  cats / traps               14.39 12.584 Inf 3.050   0.0123
##  rodenticide / traps         4.65  4.250 Inf 1.683   0.3326
##
## P value adjustment: tukey method for comparing a family of 4 estimates
## Tests are performed on the log odds ratio scale
```

```
CLD(G5.emm, Letters=letters)
```

```
##  treatment2      prob      SE  df asymp.LCL asymp.UCL .group
##  traps       0.000716 0.00053 Inf  0.000168   0.00305  a
##  rodenticide 0.003323 0.00191 Inf  0.001078   0.01020  ab
##  cats        0.010207 0.00529 Inf  0.003681   0.02798   b
##  control     0.013545 0.00626 Inf  0.005455   0.03323   b
##
## Confidence level used: 0.95
## Intervals are back-transformed from the logit scale
## P value adjustment: tukey method for comparing a family of 4 estimates
## Tests are performed on the log odds ratio scale
## significance level used: alpha = 0.05
```

### Data on GUD: 2016-wet season plot of data and fitted model

```
Ga2016W <- Ga[Ga$year==2016 & Ga$season=="wet",]

coefs <- fixef(Gglmero)

b0 <- coefs[1:4]

b1.co <- coefs[5:8]
b2.co <- coefs[21:24]

b1.ca <- coefs[ 9:12]
b2.ca <- coefs[25:28]

b1.ro <- coefs[13:16]
b2.ro <- coefs[29:32]

b1.tr <- coefs[17:20]
b2.tr <- coefs[33:36]



iv <- seq(-34,69,1)/sd.iv
iv2 <- seq(0,69,1)/sd.iv

eta.co <- b0[3] + b1.co[3]*iv  + b2.co[3]*iv^2
eta.ca <- b0[3] + b1.ca[3]*iv2 + b2.ca[3]*iv2^2
eta.ro <- b0[3] + b1.ro[3]*iv2 + b2.ro[3]*iv2^2
eta.tr <- b0[3] + b1.tr[3]*iv2 + b2.tr[3]*iv2^2

yhat.co <- 1/(1+exp(-eta.co))
yhat.ca <- 1/(1+exp(-eta.ca))
yhat.ro <- 1/(1+exp(-eta.ro))
yhat.tr <- 1/(1+exp(-eta.tr))

V <- vcov(Gglmero)

C.co <- matrix(rep(0,length(iv)*36), ncol=36)
```

```
C.co[,c(3,7,23)] <- cbind(1,iv,iv^2)

var.eta.co <- diag(C.co %*% V %*% t(C.co))
se.eta.co <- sqrt(var.eta.co)
lwr.co <- 1/(1+exp(-(eta.co-1.96*se.eta.co)))
upr.co <- 1/(1+exp(-(eta.co+1.96*se.eta.co)))

df.ctrl <- data.frame(iv=iv*sd.iv, yhat.co=yhat.co, lwr.co=lwr.co, upr.co=upr.co)

C.ca <- matrix(rep(0,length(iv2)*36), ncol=36)
C.ca[,c(3,11,27)] <- cbind(1,iv2,iv2^2)
var.eta.ca <- diag(C.ca %*% V %*% t(C.ca))
se.eta.ca <- sqrt(var.eta.ca)
lwr.ca <- 1/(1+exp(-(eta.ca-1.96*se.eta.ca)))
upr.ca <- 1/(1+exp(-(eta.ca+1.96*se.eta.ca)))

C.ro <- matrix(rep(0,length(iv2)*36), ncol=36)
C.ro[,c(3,15,31)] <- cbind(1,iv2,iv2^2)
var.eta.ro <- diag(C.ro %*% V %*% t(C.ro))
se.eta.ro <- sqrt(var.eta.ro)
lwr.ro <- 1/(1+exp(-(eta.ro-1.96*se.eta.ro)))
upr.ro <- 1/(1+exp(-(eta.ro+1.96*se.eta.ro)))

C.tr <- matrix(rep(0,length(iv2)*36), ncol=36)
C.tr[,c(3,19,35)] <- cbind(1,iv2,iv2^2)
var.eta.tr <- diag(C.tr %*% V %*% t(C.tr))
se.eta.tr <- sqrt(var.eta.tr)
lwr.tr <- 1/(1+exp(-(eta.tr-1.96*se.eta.tr)))
upr.tr <- 1/(1+exp(-(eta.tr+1.96*se.eta.tr)))

df.other <- data.frame(iv2=iv2*sd.iv, yhat.ca=yhat.ca, lwr.ca=lwr.ca, upr.ca=upr.ca,
                       yhat.ro=yhat.ro, lwr.ro=lwr.ro, upr.ro=upr.ro,
                       yhat.tr=yhat.tr, lwr.tr=lwr.tr, upr.tr=upr.tr)

# plot 95% confidence bands and points instead of lines
p <- ggplot(data=Ga2016W, aes(x=intervaldays, y=f)) +
  labs(x = "days since start treatment", y = "Giving up density") +
  theme_bw()+ theme(panel.grid.minor = element_blank()) +
  geom_point(aes(colour=treatment2, group=v.hh), size=1, position=jitter, size=0.75, alpha=0.75) +
  guides(colour = guide_legend(override.aes = list(alpha = 1), title="treatment")) +
  scale_colour_manual(values=man.col) +
  scale_x_continuous(breaks=c(-28,-14,0,14,28,42,56,70), labels=c("-28","-14","0","14","28","42","56","70")) +

  geom_ribbon(data=df.ctrl, mapping=aes(x=iv, y=yhat.co, ymin = lwr.co, ymax = upr.co, group=1),
              fill=man.col[1], alpha = .2) +
  geom_line(data=df.ctrl,  mapping=aes(x=iv, y=yhat.co, group=1),  size=1.1, colour=man.col[1]) +

  geom_ribbon(data=df.other, mapping=aes(x=iv2, y=yhat.ca, ymin = lwr.ca, ymax = upr.ca, group=1),
              fill=man.col[2], alpha = .2) +
  geom_line(data=df.other, mapping=aes(x=iv2, y=yhat.ca, group=1), size=1.1, colour=man.col[2]) +

  geom_ribbon(data=df.other, mapping=aes(x=iv2, y=yhat.ro, ymin = lwr.ro, ymax = upr.ro, group=1),
              fill=man.col[3], alpha = .2) +
  geom_line(data=df.other, mapping=aes(x=iv2, y=yhat.ro, group=1), size=1.1, colour=man.col[3]) +

  geom_ribbon(data=df.other, mapping=aes(x=iv2, y=yhat.tr, ymin = lwr.tr, ymax = upr.tr, group=1),
              fill=man.col[4], alpha = .2) +
  geom_line(data=df.other, mapping=aes(x=iv2, y=yhat.tr, group=1), size=1.1, colour=man.col[4])
p
```

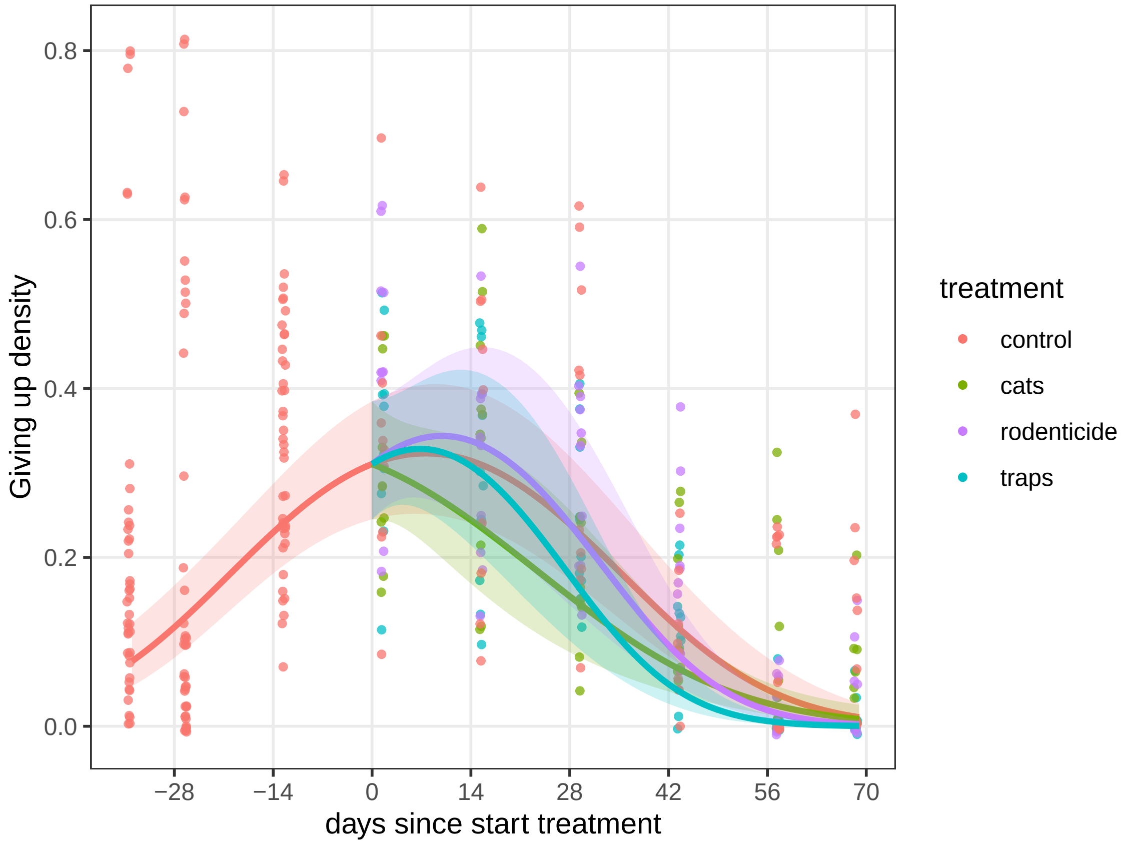


```
p <- ggplot(data=Ga2016W, aes(x=intervaldays, y=f)) +
  labs(x = "days since start treatment", y = "Giving up density") +
  theme_bw()+ theme(panel.grid.minor = element_blank()) +
  geom_point(aes(colour=treatment2, group=v.hh), size=1, position=jitter, size=0.75, alpha=0.75) +
  guides(colour = guide_legend(override.aes = list(alpha = 1), title="treatment")) +
  scale_colour_manual(values=man.col) +
  scale_x_continuous(breaks=c(-28,-14,0,14,28,42,56,70), labels=c("-28","-14","0","14","28","42","56","70")) +

  geom_ribbon(data=df.ctrl, mapping=aes(x=iv, y=yhat.co, ymin = lwr.co, ymax = upr.co, group=1),
              fill=man.col[1], alpha = .2, colour=man.col[1], size=0.2) +
  geom_line(data=df.ctrl,  mapping=aes(x=iv, y=yhat.co, group=1),  size=1.1, colour=man.col[1]) +

  geom_ribbon(data=df.other, mapping=aes(x=iv2, y=yhat.ca, ymin = lwr.ca, ymax = upr.ca, group=1),
              fill=man.col[2], alpha = .2, colour=man.col[2], size=0.2) +
  geom_line(data=df.other, mapping=aes(x=iv2, y=yhat.ca, group=1), size=1.1, colour=man.col[2]) +

  geom_ribbon(data=df.other, mapping=aes(x=iv2, y=yhat.ro, ymin = lwr.ro, ymax = upr.ro, group=1),
              fill=man.col[3], alpha = .2, colour=man.col[3], size=0.2) +
  geom_line(data=df.other, mapping=aes(x=iv2, y=yhat.ro, group=1), size=1.1, colour=man.col[3]) +

  geom_ribbon(data=df.other, mapping=aes(x=iv2, y=yhat.tr, ymin = lwr.tr, ymax = upr.tr, group=1),
              fill=man.col[4], alpha = .2, colour=man.col[4], size=0.2) +
  geom_line(data=df.other, mapping=aes(x=iv2, y=yhat.tr, group=1), size=1.1, colour=man.col[4])
p
```

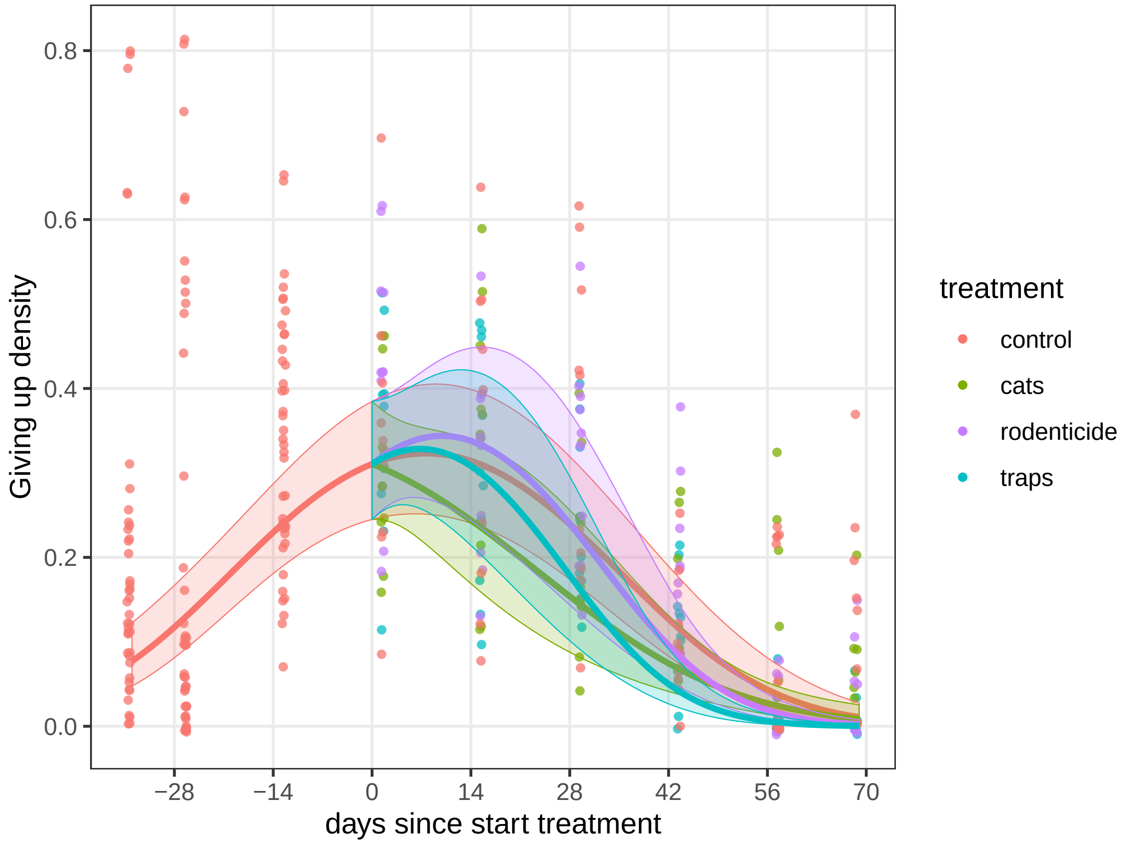
Giving up density
0.8
0.6
0.4
0.2
0.0
−28
−14
0
14
28
42
56
70
days since start treatment
treatment
control
cats
rodenticide
traps
